# Conformational Analysis Explores the Role of Electrostatic Nonclassical CF···HC Hydrogen Bonding Interactions in Selectively Halogenated Cyclohexanes

**DOI:** 10.1021/acs.joc.3c02868

**Published:** 2024-03-05

**Authors:** Mengfan He, Bruno A. Piscelli, Rodrigo A. Cormanich, David O’Hagan

**Affiliations:** † School of Chemistry, Biomedical Sciences Research Complex, University of St Andrews, North Haugh, St Andrews, Fife KY16 9ST, United Kingdom; ‡ Instituto de Química, Universidade Estadual de Campinas (UNICAMP), Monteiro Lobato Street, Campinas, Sao Paulo 13083-862, Brazil

## Abstract

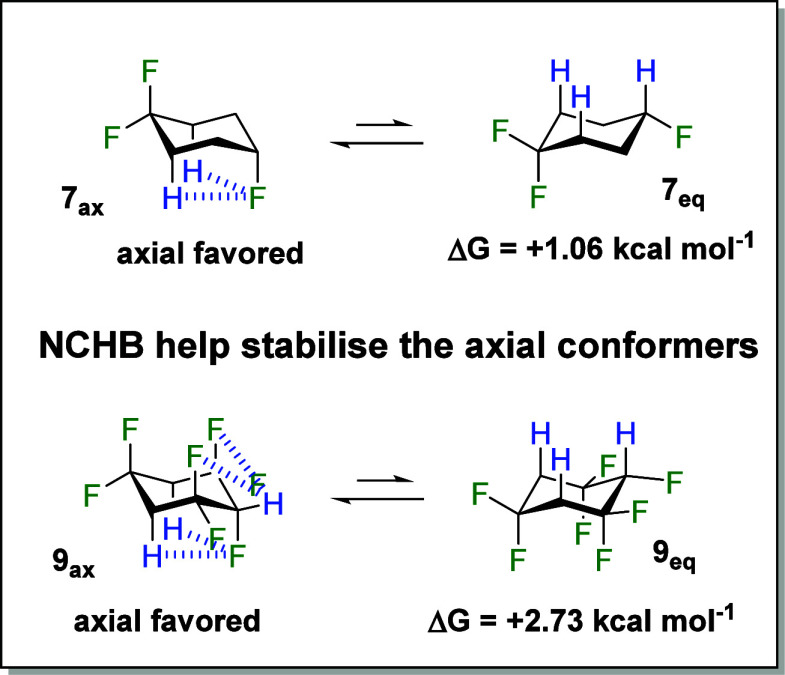

The conformational equilibria of selectively halogenated cyclohexanes are explored both experimentally (VT-NMR) for 1,1,4,-trifluorocyclohexane **7** and by computational analysis (M06-2X/aug-cc-pVTZ level), with the latter approach extending to a wider range of more highly fluorinated cyclohexanes. Perhaps unexpectedly, **7**_ax_ is preferred over the **7**_eq_ conformation by Δ*G* = 1.06 kcal mol^–1^, contradicting the accepted norm for substituents on cyclohexanes. The axial preference is stronger again in 1,1,3,3,4,5,5,-heptafluorocyclohexane **9** (Δ*G* = 2.73 kcal mol^–1^) as the CF_2_ groups further polarize the isolated CH_2_ hydrogens. Theoretical decomposition of electrostatic and hyperconjugative effects by natural bond orbital analysis indicated that nonclassical hydrogen bonding (NCHB) between the C-4 fluorine and the diaxial hydrogens at C-2 and C-6 in cyclohexane **7** and **9** largely accounts for the observed bias. The study extended to changing fluorine (F) for chlorine (Cl) and bromine (Br) at the pseudoanomeric position in the cyclohexanes. Although these halogens do not become involved in NCHBs, they polarize the geminal −CHX– hydrogen at the pseudoanomeric position to a greater extent than fluorine, and consequent electrostatic interactions influence conformer stabilities.

## 1 Introduction

**Figure 1:**
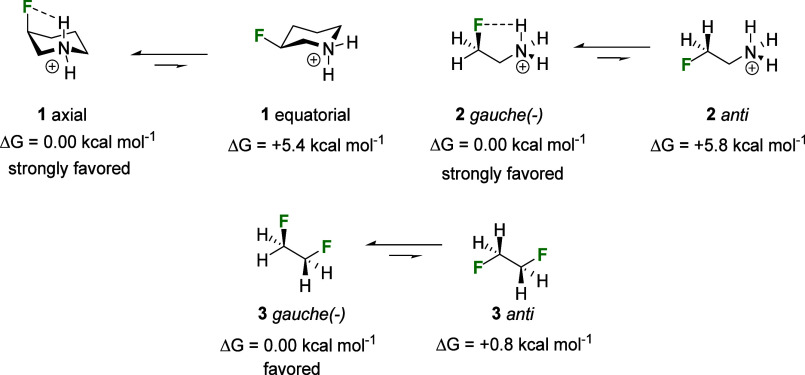
Conformational energy preferences favoring axial energy for **1** and *gauche* energy for **2** and **3**.^6,7^

The C–F bond is the most polar in organic chemistry and selective fluorination can impart an unusual polarity, particularly in aliphatic compounds.^1,2^ Such polarity can induce contraintuitive behavior in organic molecules such as is described in the *gauche* preference for 1,2-difluoroethane (*gauche* effect)^3^ and the strong axial preference in 2-fluorotetrahydropyran (anomeric effect).^4^ The high electronegativity of fluorine significantly reduces the ability of carbon bound fluorine to enter into classical hydrogen bonding relative to nitrogen and oxygen;^5^ however, polarized C–F bonds can make stabilizing contacts to hydrogens through dipolar electrostatic interactions. This is evinced most dramatically in the equilibrium conformations of the 3-fluoropyridinium ring **1** as illustrated in Figure 1.^6^ The conformer with the fluorine axial is 5.4 kcal mol^–1^ more stable than that when the fluorine is equatorial with the C–F and the H–N^+^ running parallel with compensating dipoles. The F···H–N^+^ bonding angle here is 90°, which is notably not at all optimal for a classical hydrogen bond; however, the dipolar interaction between the axial C–F and the axial N^(+)^-H bonds is strong, and this contributes significantly to the stabilization observed. An “electrostatic *gauche* effect” of a similar magnitude is also observed in 2-fluoroethylammonium **2**, where the **2**-*gauche* conformer is significantly more stable than the **2**-*anti* conformer by 5.8 kcal mol^–1^.^7^ For reference, these effects are much stronger than that observed in the classical *gauche* effect, which recognizes that the **3**-*gauche* conformer of 1,2-difluoroethane is more stable that the **3**-*anti* conformer by up to 0.8 kcal mol^–1^.^3,8^ In that case, the origin of the *gauche* effect in 1,2-difluoroethane and related neutral systems, as determined principally by electrostatics or hyperconjugation, remains an open discussion as both effects can make a significant contribution to the relatively small energy differences between the *gauche* and *anti* conformers.^8,9^

**Figure 2:**
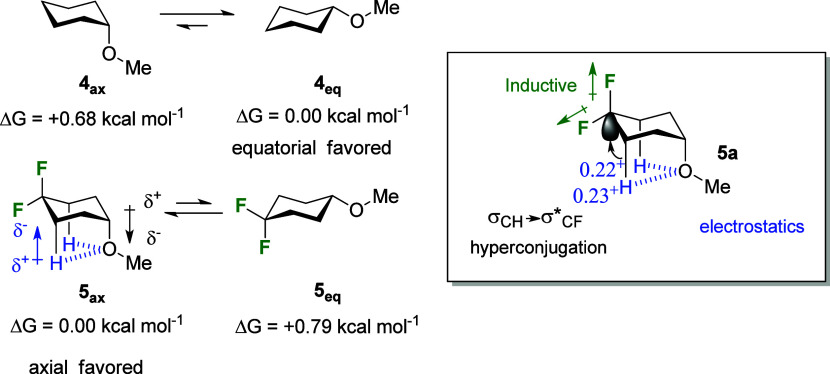
Conformational preferences for methoxycyclohexane **4** and difluorinated analogue **5** (values are calculated in gas phase calculations). The axial preference for **5** can be rationalized by a combination of NCHB (electrostatics), inductive effects, and hyperconjugation (see inset summary).^10^

In a tangential study, we recently reported that the introduction of fluorines into the 4-position of methoxycyclohexane **4** to generate **5** results in switching the relative energies of the axial and equatorial conformers as illustrated in Figure 2.^10^ Methoxycyclohexane **4** has an equatorial preference consistent with well-known effects of cyclohexanes, whereas difluoro derivative **5** has an axial preference. We attributed this largely to electrostatic effects. The outcomes were supported by a combination of experimental (VT-NMR) and DFT calculations in the NBO framework. The preference for the axial methoxyl in **5** is assigned to nonclassical hydrogen bonding (NCHB) stabilization between the methoxyl oxygen and the electropositive diaxial hydrogens at C-2 and C-5 of the cyclohexane ring as illustrated in the inset in Figure 2. These hydrogens are polarized by the vicinal fluorines, and a stabilizing 1,3-diaxial electrostatic interaction occurs when the OMe substituent is axial. Such transannular interactions are increasingly being discussed in the context of the anomeric effect.^11^ These interactions come into the category of nonclassical hydrogen bonds (NCHBs), with weak C–H hydrogen bonding donors and H···OR angles of 90°, a geometry not optimal for lone pair-antibonding orbital interactions in classical hydrogen bonding.^12^

In this paper, these effects are further explored by replacing the methoxyl group with fluorine to consider 1,1,4-trifluorocyclohexane **7**. It is generally accepted that fluorine is a poorer hydrogen bonding acceptor than the oxygen in a methoxyl (-OMe) group; thus, the transannular interaction may be expected to weaken. However, this study reveals that there is a greater axial over equatorial preference observed for **7** than is found in **5**. In the preparation of this manuscript, we located an abstract of the American Chemical Society (ACS) National Meeting in 1990, from Stolow and Kao, which indicated that cyclohexane **7** had been prepared and found to have an axial (10_ax_:1_eq_) preference in solution (FCCl_3_) at 170 K (Δ*G*^0^ = −0.77 ± 0.02 kcal mol^–1^).^13^ We cannot identify a paper disclosing further details; however, our conclusions certainly support the observation disclosed in the ACS Abstract.

## 2 Results and Discussion

**Scheme 1:**
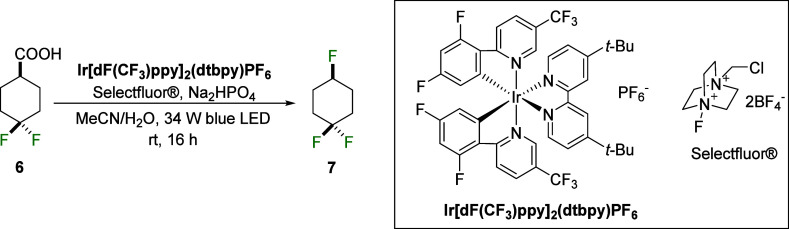
Synthesis of **7***via* Decarboxylative Fluorination of **6**(14)

The study aimed to combine and compare the outcomes from both computational calculations and VT-NMR experiments to explore the conformational preference of cyclohexane **7**. In order to carry out VT-NMR analysis, a sample of 1,1,4-trifluorocyclohexane **7** was required. A direct approach by treatment of 1,1-difluorocyclohexanol with diethylaminosulfur trifluoride (DAST) failed giving only a complex mixture including elimination products; however, success was achieved by a highly efficient decarboxylative fluorination of carboxylic acid **6** following a photocatalytic protocol of MacMillan et al.^14^ as illustrated in Scheme 1.

Carboxylic acid **6** was completely consumed in the reaction, and the volatile cyclohexane **7** was extracted into diethyl ether, and then the solvent was carefully removed under vacuum. This preparation of **7** was used for subsequent variable-temperature nuclear magnetic resonance (VT-NMR) analysis.

### 2.1 VT-NMR Analysis

**Figure 3:**
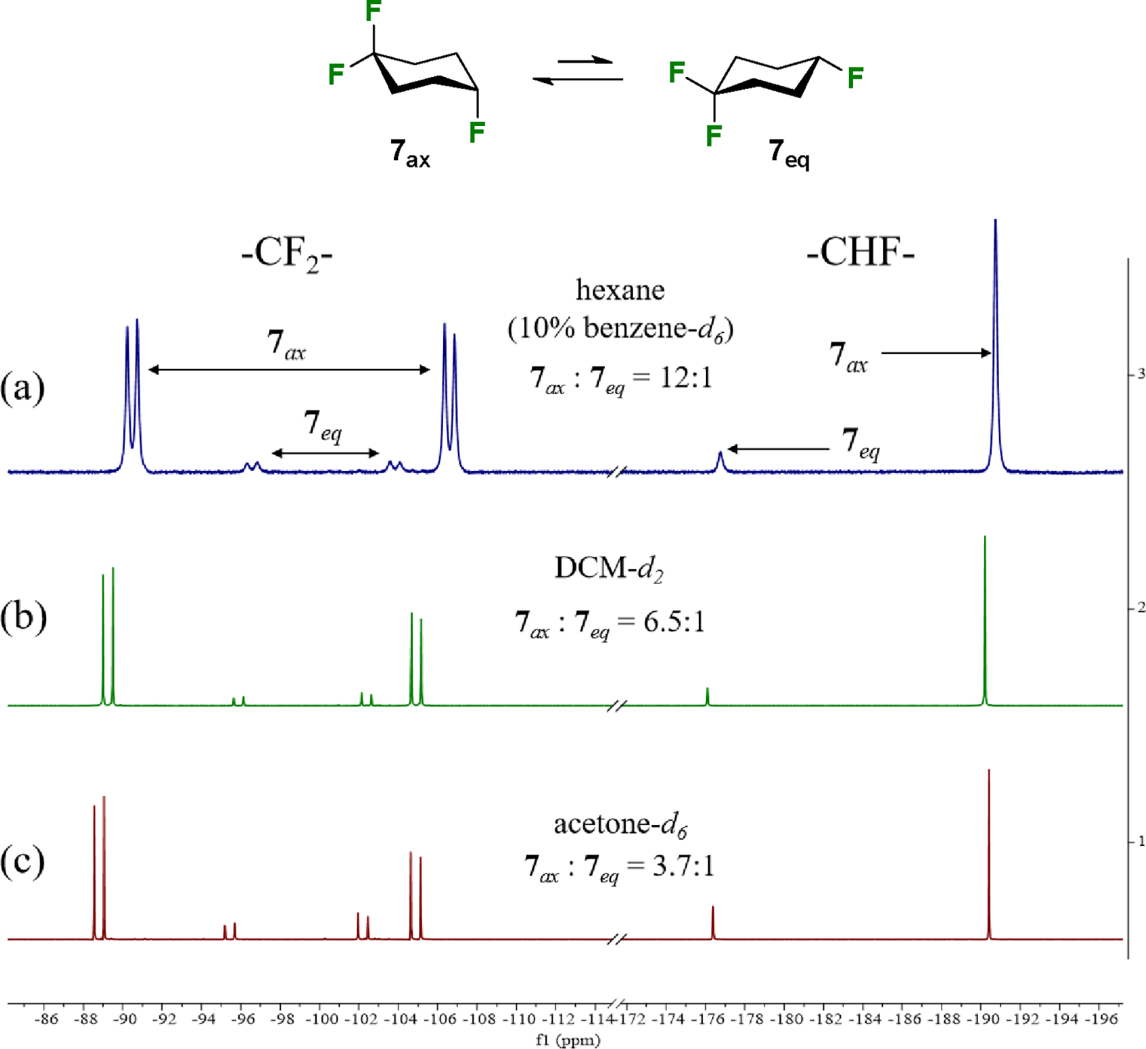
Low temperature (195 K (−78 °C)) ^19^F{^1^H}-NMR spectra of **7**, showing the signals for conformers **7**_ax_ (major) and **7**_eq_ (minor) in different solvents; (a) hexane (benzene-*d*_6_ 10%); (b) dichloromethane-*d*_2_; (c) acetone-*d*_6_.

^19^F{^1^H}-NMR analysis was used to explore the conformational equilibria of **7**. Ring inversion is too rapid at room temperature to resolve individual signals for **7**_ax_ and **7**_eq_; however, these were readily resolved at lower temperature. Accordingly, ^19^F{^1^H}-NMR analysis of **7** was conducted at 195 K (−78 °C) in three different solvents (hexane (10% benzene-*d*_6_), dichloromethane-*d*_2_, and acetone-*d*_6_) and the outcomes are illustrated in Figure 3.

The assignments for each conformer were determined by applying proton coupling and recording ^19^F-NMR spectra.^15^ Each conformer has a signal with a large geminal ^2^*J*_HF_ coupling (∼46 Hz) associated with the fluorine at C-4; however, **7**_ax_ also has a large ^3^*J*_HF_ antiperiplanar coupling (∼46 Hz), whereas this is much reduced in **7**_eq_ (see Figure S4). In the ^19^F{^1^H}-NMR, the diastereotopic geminal CF_2_ group at C-1 constitutes an AB system and appears as a doublet of doublets (**7**_ax_, ^2^*J*_FF_ = 234 Hz; **7**_eq,_^2^*J*_FF_ = 236 Hz), with the fluoromethylene (CHF) signal resonating upfield as a singlet. The major **7**_ax_ and minor **7**_eq_ conformers are clearly resolved as shown in Figure 3.

The **7**_ax_ conformer dominates over the **7**_eq_ in all cases and most significantly in hexane (12:1), although the ratios decrease as the polarity of the solvent increases, consistent with an electrostatic screening effect. In the case of hexane, it proved necessary to add-mix a deuterated solvent to provide an NMR “lock”. Thus, the hexane experiments were conducted with 10% benzene-*d*_6_ added, which will have a modest effect on the dielectric constant of the medium.

### 2.2 Theory Analysis

#### 2.2.1 Cyclohexane **7**

**Figure 4:**
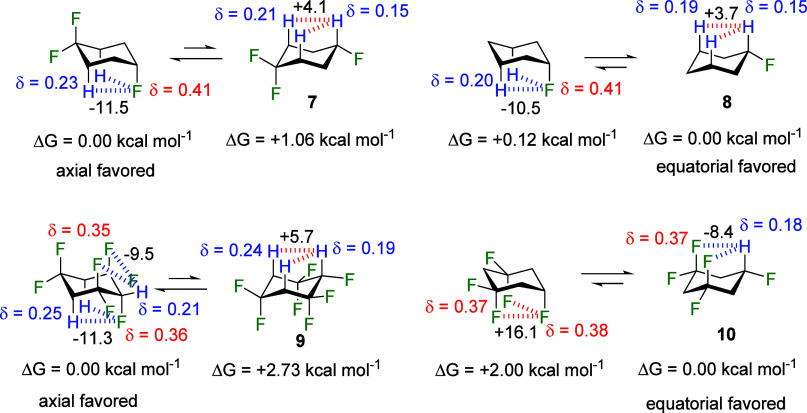
Conformational equilibria of fluorocyclohexanes **7** and **8** and analogues **9** and **10**. Electrostatic interactions, equilibria energies (kcal mol^–1^) and atomic charges calculated in the gas-phase at the M06-2X/aug-cc-pVTZ theoretical level. Hydrogens in 1,3-relationships are chemically equivalent.

Theoretical calculations were carried out to complement the experimental observations, and they were extended to the additionally fluorinated cyclohexanes **9** and **10**, as well as fluorocyclohexane **8**, which was used as a reference compound. Calculations were run in the gas phase and using the IEFPCM implicit solvent model^16^ at the M06-2X/aug-cc-pVTZ level. Outcomes for **8** were close to that of a number of previous assessments which also marginally favor the **8**_eq_ conformer.^17^ The outcomes here for cyclohexanes **7**–**9** are illustrated in Figure 4.

At the outset, the relative energies of **7**_ax_ and **7**_eq_ were explored. A significant energy difference of −1.06 kcal mol^–1^ was observed between **7**_ax_ and **7**_eq_, favoring the axial conformer, in agreement with experiment. Notably, the difference in the axial preference for cyclohexane **7** (1.06 kcal mol^–1^) was larger when compared to methoxy-cyclohexane **5** (0.79 kcal mol^–1^). Natural bond orbital (NBO)^17^ analysis was used to deconvolute the total energy contributions from hyperconjugation, electrostatic interactions, and steric effects on the electronic energy between the axial and equatorial conformers of **7** (Table 1). This analysis revealed that electrostatic interactions (−ve values) govern the observed axial preference, whereas both hyperconjugative and steric effects (+ve values) drive the equilibrium toward the equatorial conformer.

**Table 1:** Gas-Phase Calculated Gibbs Free Energy (Δ*G*), Total Electronic Energy (Δ*E*) and NBO Analysis for Hyperconjugative (Δ*E*_*NL*_), Electrostatic (Δ*E*_*NCE*_) and Steric (Δ*E*_*NSA*_) Contributions to the Total Electronic Energies of Compounds **5**, **7**, and **9**–**16** at the M06-2X/aug-cc-pVTZ Theoretical Level, in kcal mol^–1^^b^

**Compound**	**Δ***G*	**Δ***E*	(hyperconjugation) **Δ***E*_*NL*_	(electrostatics) **Δ***E*_NCE_	(sterics) **Δ***E*_*NSA*_
**5**^a^	–0.79	–1.08	+2.65	–3.50	+3.38
**7**	–1.06	–1.10	+1.41	–3.24	+1.93
**8**	+0.12	+0.08	+1.61	+3.76	+2.00
**9**	–2.73	–2.81	+8.39	–20.29	+1.01
**10**	+2.00	+2.16	–3.67	+5.16	–0.75
**11**	–0.70	–0.93	+0.18	–5.81	+2.58
**12**	–0.67	–0.94	–0.56	–6.14	+2.69
**13**	–1.41	–1.66	+2.66	–10.38	+2.18
**14**	–0.92	–1.23	+1.02	–8.00	+3.28
**15**	+3.70	+3.64	–1.40	+3.21	+0.28
**16**	+4.04	+3.87	–0.78	+2.94	+0.73

a Values obtained from ref (10b).

b The Δ energies are considered as (*ax*–*eq*), thus negative energy values represent axial preference, and the positive ones represent equatorial preference

Interestingly for cyclohexane **7**, a significant axial stabilization arises from the σ_CH_ → σ*_CF_ hyperconjugative interactions, with a notable interaction energy of 5.81 kcal mol^–1^ in **7**_ax_ compared to 1.02 kcal mol^–1^ in **7**_eq_. This finding aligns with the well-established hyperconjugative model used to elucidate the anomeric effect in different systems,^19^ where an antiperiplanar arrangement between an *endo*-cyclic donor group (σ_CH_ orbitals or 2p-type lone pair from oxygen, for example) and the antibonding orbital of an *exo*-cyclic C-X bond (*n*_O_ → σ*_CX_ or σ_CH_ → σ*_CX_ interactions) is a driving force for the axial preference. Nevertheless, the cumulative effect of all hyperconjugative interactions in **7** still leads to favoring equatorial stabilization, but it is the electrostatic factor (Δ*E*_NCE_), which tips the overall balance in favor of **7**_ax_ (detailed interactions addressed in the ESI).

**Figure 5:**
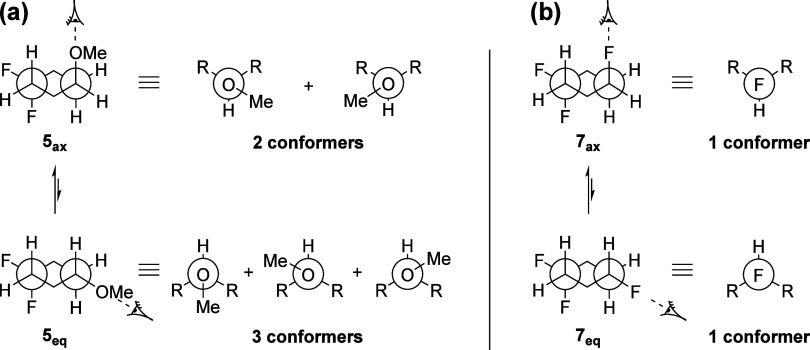
(a) Rational for entropic destabilization of **5**_ax_ (OMe) relative to (b); the **7**_ax_ (F) conformer due to the additional conformers associated with the −OMe relative to the −F substituent.

Furthermore, the driving force behind **7**_ax_ stabilization is identified as the formation of 1,3-diaxial CH···FC nonconventional hydrogen bonds (NCHBs), each providing ∼11.5 kcal mol^–1^ of electrostatic stabilization to **7**_ax_ as delineated in Table 2. The critical role of NCHBs in stabilizing the axial conformation of fluorocyclohexanes is underscored by fluorocyclohexane **8**. In this case, the absence of CF_2_ groups results in less polarization of axial hydrogens, thus weakening the NCHBs (−10.5 kcal mol^–1^ each). As a result, electrostatic interactions, in addition to hyperconjugative and steric effects, become equatorial stabilizing, leading to a slight shift of the equilibrium favoring the equatorial conformer. Interestingly, each NCHB in this context was found to be weaker than the corresponding interactions in methoxycyclohexane **5**_ax_, which rendered ∼16.8 kcal mol^–1^ of electrostatic stabilization for each CH···OC. This aligns with the general expectation that fluorine is a less effective hydrogen bond acceptor than oxygen.^5^ Importantly, the stronger axial preference in cyclohexane **7**, compared to that of **5**, cannot be assigned to stronger NCHBs. Instead, it is tied to an entropic penalty associated with the rotation of the C-OMe bond in the axial conformer of **5**, similar to observations well-known in alkyl-substituted cyclohexanes.^20^ As illustrated in Figure 5a, there are two accessible conformers for **5**_ax_, where the methoxyl group extends outside the ring, while in **5**_eq_, due to the equatorial orientation of the methoxyl group, there are three possible conformers. Consequently, the axial conformer (**5**_ax_) in this case has less degrees of freedom, resulting in a relative entropic penalty (*T*Δ*S* = 0.21 kcal mol^–1^), which marginally shifts the equilibrium toward the equatorial conformer (**5**_eq_). By contrast, compound **7** lacks such an entropic effect since rotation around the C–F bond does not induce any conformational change (Figure 5b). Consequently, there is no relative conformational entropy associated with the **7**_ax_/**7**_eq_ conformer equilibrium (*T*Δ*S* = 0.02 kcal mol^–1^).

**Table 2:**
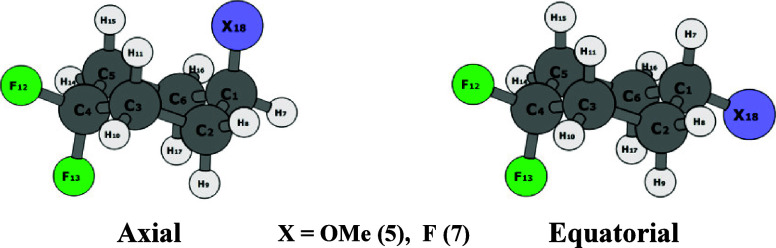
Individual Interaction Energies in **5**_ax_/**5**_eq_ and **7**_ax/_**7**_eq_ Contributing to the Global Δ*E*_NL_, Δ*E*_NCE_, and Δ*E*_NSA_ Obtained at the M06-2X/aug-cc-pVTZ Theoretical Level, in kcal mol^–1^

	**Δ***E*_NL_ (hyperconjugation)	**Δ***E*_NCE_ (electrostatics)	**Δ***E*_NSA_ (sterics)
**5**_ax_	σ_C2H9_ → σ*_C1O_ = 5.53	CH···OC NCHB – 16.8	σ_C1O18_/σ_C2H8_ = 0.57
	σ_C2H9_ → σ*_C1H_ = <0.5		σ_C1O18_/σ_C2H9_ = 4.29
**5**_eq_	σ_C2H9_ → σ*_C1O_ = 0.82	C1H7···H11C3 + 4.1	σ_C1O18_/σ_C2H8_ = 0.61
	σ_C2H9_ → σ*_C1H_ = 3.63		σ_C1O18_/σ_C2H9_ = 0.80
**7**_ax_	σ_C2H9_ → σ*_C1F_ = 5.81	CH···FC NCHB – 11.5	σ_C1F18_/σ_C2H8_ = 0.77
	σ_C2H9_ → σ*_C1H_ = <0.5		σ_C1F18_/σ_C2H9_ = 4.03
**7**_eq_	σ_C2H9_ → σ*_C1F_ = 1.02	C1H7···H11C3 + 4.1	σ_C1F18_/σ_C2H8_ = 0.66
	σ_C2H9_ → σ*_C1H_ = 3.30		σ_C1F18_/σ_C2H9_ = 0.79

Implicit solvation effects with increasing dielectric constants of the three solvents used experimentally (hexane, dichloromethane, and acetone) were then considered. In each case, this reduced the energy between the **7**_ax_ and **7**_eq_ conformers, consistent with electrostatic screening, and the consequent change in ratios (increase in **7**_eq_ population) observed by VT-NMR (Table 3).

**Table 3:** Comparison of the Experimentally Determined (VT-NMR) and Computationally Derived Conformer Ratios for **7** in Different Solvents

	**7**_ax_: **7**_eq_ ratios	
**NMR solvent**	**experiment**	**theory**
hexane (10% benzene-*d*_6_)	12:1	10.5:1
DCM-*d*_2_	6.5:1	5.5:1
acetone-*d*_6_	3.7:1	5.2:1

However, in all cases, the axial conformer **7**_ax_ was dominant, consistent with experiment. Other twist boat conformers were considered, but they were >5 kcal mol^–1^ higher than the axial conformers and were judged not to have any significant contribution to the conformer population in solution (Figure S6 in the SI).

**Figure 6:**
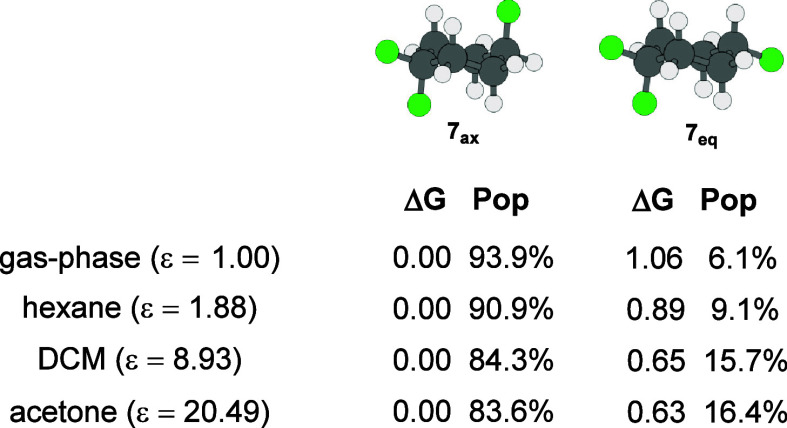
Calculated relative Gibbs free energies (Δ*G*) in kcal mol^–1^ and populations (Pop) in percentage for **7**_ax_ and **7**_eq_ in gas-phase and implicit solvents (hexane, dichloromethane, and acetone), at the M06-2X/aug-cc-pVTZ/IEFPCM level of theory.

#### 2.2.2 Cyclohexanes **9** and **10**

In order to explore these phenomena further in a theoretical context, the introduction of three CF_2_ groups into the ring was explored for cyclohexane **9**. This resulted in a nearly identical NCHB interaction energy compared to **7** (−11.5 kcal mol^–1^). This similarity arises despite the higher polarization of the 2,6-diaxial hydrogens, presumably as the CF_2_ groups also withdraw electron density from the fluorine at position 4 of the ring, reducing its electron density. Nonetheless, the axial preference over the equatorial preference in cyclohexane **9** significantly increases, favoring **9**_ax_ by 2.73 kcal mol^–1^.

In the context of the fluorine-substituted compounds, where CH···FC NCHBs are weaker compared with their CH···OMe analogues, other electrostatic interactions also play a significant role in determining the conformational equilibria in **9**. For example, in addition to stabilizing NCHBs, 1,3-F_ax_···H_eq_ interactions greatly stabilize **9**_ax_ by −9.5 kcal mol^–1^_._ Meanwhile, 1,3-H_ax_···H_ax_ electrostatic contacts notably destabilize the equatorial conformer **9**_eq_ (+5.7 kcal mol^–1^), substantially enhancing the observed axial preference. These electrostatic interactions are also reflected in the observed ring strains for compounds **9**_**ax**_ and **9**_**eq**_. Specifically, when examining all C–C–C–C dihedral angles involving adjacent carbon atoms in the ring, as well as those encompassing the hydrogen and fluorine atoms at the monofluorinated carbon, the average calculated dihedral angle is −40.7 degrees for **9**_**ax**_ and −39.7 degrees for **9**_**eq**_ (Table 4). In the latter case, this represents a 2° difference in comparison to the unsubstituted cyclohexane’s dihedral angle of −41.7 degrees. The tighter average dihedral angle in **9**_**eq**_ indicates an increase in ring strain in the equatorial conformer, consequently resulting in its destabilization. This effect is not as pronounced in compounds **7** and **8** due to the weaker electrostatic interactions in these cases. In addition, the geminal F–C–H angle in the monofluorinated carbon is slightly wider in **9**_**ax**_ (109.9°) compared to that in **9**_**eq**_ (109.0°), highlighting the shorter CH_ax_···F_ax_C contact in the former and longer 1,3-H_ax_···H_ax_ contact in the latter.

**Table 4:**
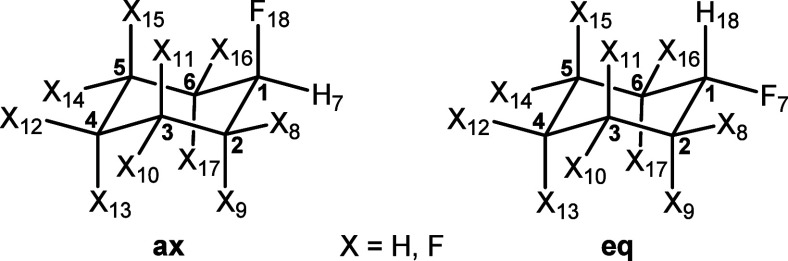
Selected 3-Atom Angles and 4-Atom Dihedrals (Degrees) from the Gas-Phase Optimized Structures of Unsubstituted Cyclohexane and Compounds **7**–**10**, Obtained at the M06-2X/aug-cc-pVTZ Theoretical Level

**dihedral (ω)**	**cyclohexane**	**7**_**ax**_	**7**_**eq**_	**8**_**ax**_	**8**_**eq**_	**9**_**ax**_	**9**_**eq**_	**10**_**ax**_	**10**_**eq**_
1–2–3–4	55.7	53.5	54.5	54.8	55.9	51.4	51.4	50.3	53.3
2–3–4–5	–55.7	–55.0	–53.8	–56.8	–55.6	–50.3	–50.5	–49.8	–48.9
3–4–5–6	55.7	55.0	53.8	56.7	55.7	50.2	50.5	49.8	48.9
7–1–2–3	–178.5	–178.9	–178.6	–177.9	–178	–175.7	–174.6	–171.5	–176.4
7–1–2–9	–58.3	–58.0	–58.1	–57.2	–57.8	–52.5	–51.1	–52.0	–57.1
18–1–2–3	64.4	64.9	64.7	66.3	65.5	64.4	66.4	72.1	66.1
18–1–2–9	–175.4	–174.2	–174.9	–173	–174.3	–172.3	–170.1	–168.4	–174.5
average	–41.7	–41.8	–41.8	–41.0	–41.2	–40.7	–39.7	–38.5	–41.2

**angle (∠)**	**cyclohexane**	**7**_**ax**_	**7**_**eq**_	**8**_**ax**_	**8**_**eq**_	**9**_**ax**_	**9**_**eq**_	**10**_**ax**_	**10**_**eq**_
1–2–3	111.1	111.3	110.3	111.5	110.3	113.0	112.7	112.9	110.3
2–1–6	111.1	112.5	111.7	112.6	112.0	112.0	112.2	112.8	111.4
2–1–7	110.4	110.7	109.0	110.8	109.1	111.3	111.7	114.0	114.1
2–1–18	109.0	108.2	110.1	108.1	110.0	110.4	109.8	109.3	108.5
2–3–4	111.1	110.4	110.5	111.0	111.1	107.3	108.2	109.3	110.5
3–4–5	111.1	113.8	113.9	110.7	111.0	114.2	113.8	111.9	111.7
7–1–18	106.9	106.4	106.7	106.1	106.5	109.9	109.0	106.7	107.4
average	110.1	110.5	110.3	110.1	110.0	111.2	111.1	111.0	110.6

It is worth noting that the primary factor for axial stabilization in **9**_ax_ remains the 1,3-diaxial F_ax_···H_ax_ NCHBs. This is evident when the stabilizing CH_ax_···F_ax_ interactions are replaced by destabilizing CF_ax_···F_ax_ contacts, as illustrated in pentafluorocyclohexane **10**. In this case, the emergence of F···F repulsions (+16.1 kcal mol^–1^ each) in **10**_ax_ leads to a preference for the **10**_eq_ conformer by 2.00 kcal mol^–1^ and results in an elevated torsional strain in **10**_**ax**_, illustrated by its considerably tighter average dihedral angle (−38.5°), 3.2° higher than the average dihedral angle of an unsubstituted cyclohexane (−41.7°). Furthermore, the stability of **10**_**eq**_ is reinforced by electrostatic transannular NCHBs, each contributing −8.4 kcal mol^–1^ in further stability to **10**_eq_. Similar to **9**, the F–C–H angle in the monofluorinated carbon is slightly wider in **10**_**eq**_ (107.4°) in order to favor NCHB formation compared to **10**_**ax**_ (106.7°). Again, the increase in solvent polarity attenuates the electrostatic NCHB interactions, resulting in compounds **8**_**ax**_, **9_ax_**, and **10**_**eq**_ becoming progressively less stable going from hexane to dichloromethane and acetone (see Table S3), consistent with an electrostatic screening.

#### 2.2.3 Comparison with Other Halogens in Cyclohexanes **11**–**16**

**Figure 7:**
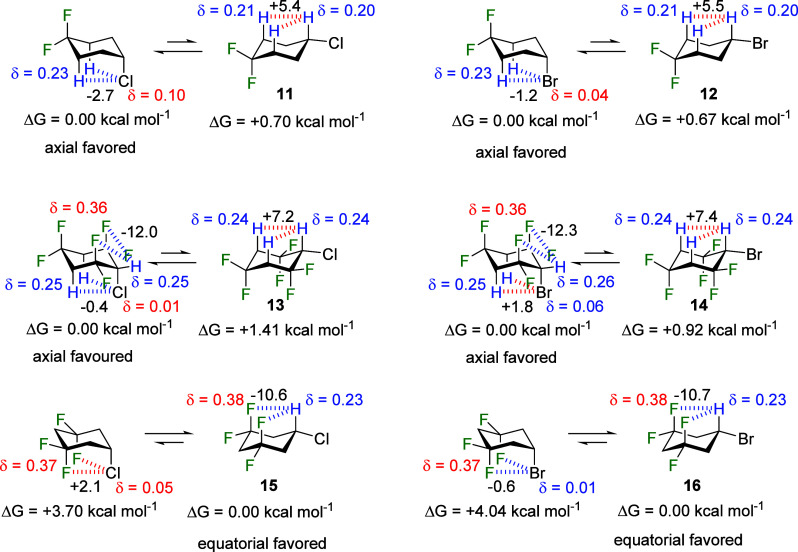
Conformational equilibria of compounds **11**–**16**. Electrostatic interaction energies (kcal mol^–1^) and atomic charges were calculated in Gas-Phase at the M06-2X/aug-cc-pVTZ theoretical level. Hydrogens in 1,3-relationships are chemically equivalent.

**Figure 8:**
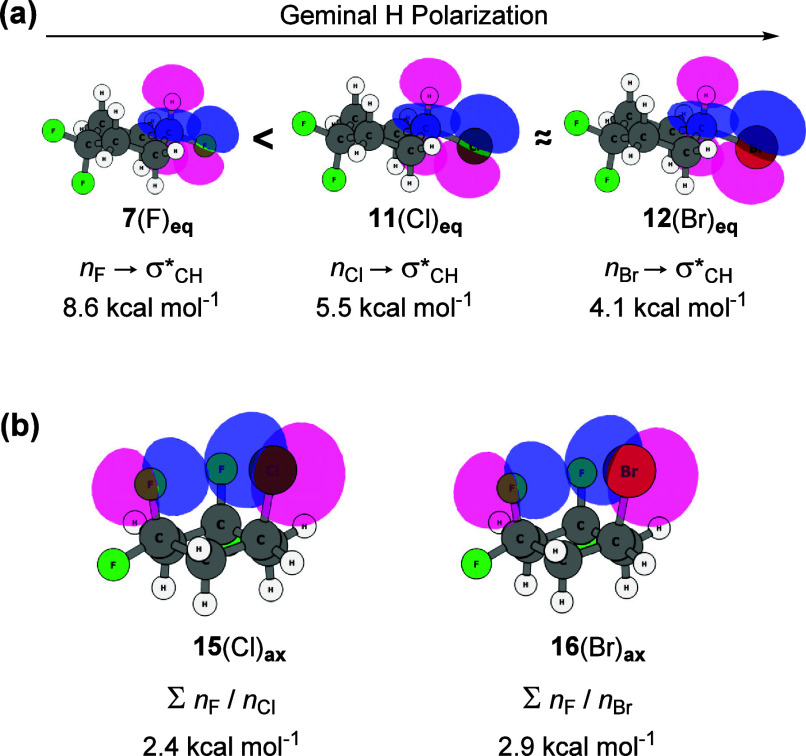
Outcomes at the M06-2X/aug-cc-pVTZ theory level: (a) *n*_X_ → σ*_CH_ hyperconjugative interaction that decreases the atomic charge of the geminal H in compounds **7**_**eq**_, **11**_eq_, and **12**_eq_. (b) Sum of *n*_F_/*n*_X_ steric repulsion energies that destabilize the **15**_ax_ and **16**_ax_ conformers.

Given the significant impact of electrostatic interactions, particularly NCHBs, on the axial stabilization observed for fluorine, this study was extended also to chlorinated and brominated cyclohexane analogues to explore how these interactions evolve with different halogens. These halogens do not make strong hydrogen bond acceptors when bound to carbon.^21^ The relative energies and contributions to stability were considered for cyclohexanes **11**-**16**. Interestingly, electrostatic interactions continue to dictate the conformational equilibria in these cases; however, owing to the lower electronegativity of Cl and Br, electrostatic interactions other than NCHBs emerge as the predominant factors. For instance, in compounds **11** and **12**, the 1,3-diaxial NCHBs to chlorine and bromine exhibit only a weak axial stabilization (−2.7 and −1.2 kcal mol^–1^, respectively), as shown in Figure 7. Instead, the axial preference is primarily influenced by destabilizing 1,3-^δ+^H_ax_···^δ+^H_ax_ interactions in the equatorial conformer, with each contributing approximately 5.5 kcal mol^–1^ in destabilizing energy. It is noteworthy that the hydrogen atom geminal to the halogen substituent in **11**(Cl) and **12**(Br) is more positively charged compared to that of the fluorinated analogue **7**, despite the higher electronegativity of fluorine. This counterintuitive observation arises from the better superposition between *n*_F_ lone pairs and σ*_CH_ antibonding orbitals, resulting in a more effective charge transfer back to the hydrogen atom through hyperconjugation, compared to the same effect for **11**(Cl) and **12**(Br) (Figure 8a). Consequently, the axial preferences diminish in comparison to **7** (F), reducing to 0.70 kcal mol^–1^ in **11** (Cl) and 0.67 kcal mol^–1^ in **12** (Br).

Calculations extended to cyclohexanes **13** (Cl) and **14** (Br), which have more CF_2_ groups in the ring. This further polarizes the hydrogen atoms, and the halogen substituents shift toward virtually neutral (−0.01 au in Cl) or positive (+0.06 au in Br) atomic charges. Consequently, 1,3-H_ax_···X_ax_ electrostatic interactions become very weak (−0.4 kcal mol^–1^) in **13** (Cl) or repulsive (+1.8 kcal mol^–1^) in **14** (Br). In both cases, the axial preference is once again assigned to stabilizing 1,3-^δ-^F_ax_···^δ+^H_eq_ interactions in the axial conformers (∼12 kcal mol^–1^ each) and destabilizing 1,3-^δ+^H_ax_···^δ+^H_ax_ electrostatic contacts (∼7 kcal mol^–1^ each) in the equatorial counterparts. Notably, the higher axial preference in chlorinated analogue **13** (1.41 kcal mol^–1^) compared to brominated compound **14** (0.92 kcal mol^–1^) is attributed, among other smaller electrostatic interactions, to the modestly stabilizing 1,3-^δ+^H_ax_···^δ-^Cl_ax_ interactions as opposed to similarly destabilizing 1,3-^δ+^H_ax_···^δ+^Br_ax_ interactions.

Despite the absence of nonconventional hydrogen bonds involving chlorine and bromine in the cyclohexanes studied, the introduction of CF_2_ groups at positions 3 and 5 of the ring, as observed in compounds **15** (Cl) and **16** (Br), induces a substantial shift in the equilibria toward the equatorial conformers by 3.70 and 4.04 kcal mol^–1^, respectively. This is assisted by the formation of strong 1,3-F_ax_···H_ax_ NCHBs in the equatorial conformers, contributing −10.6 kcal mol^–1^ in **15** (Cl) and −10.7 kcal mol^–1^ in **16** (Br) in stabilizing electrostatic energy, respectively. Notably, despite the comparable values for these NCHBs in both cases, the global difference in the magnitude of the equatorial preference can be attributed to higher steric hindrance in **16**_ax_ (Br) resulting from more repulsive *n*_Br_/*n*_F_ orbital interactions, compared to similar *n*_Cl_/*n*_F_ interactions in **15** (Cl) (Figure 8b).

## 3 Conclusions

In summary, a clear role for electrostatic nonclassical CF···HC hydrogen bonding interactions is demonstrated in influencing the conformational equilibrium of selectively halogenated cyclohexanes. Notably, a pseudoanomeric effect is observed for 1,1,4-trifluorocyclohexane **7** perhaps unexpectedly showing a bias for the **7**_ax_ over the **7**_eq_ conformer. A comparison is made between the experimental data in solution and in different solvents and that established by theory in the gas phase. Conformer populations established by experiment and theory for **7** are relatively close as summarized in Table 3. The theory approach was able to deconvolute electrostatic from hyperconjugative contributions to the relative stabilization of the halogenated cyclohexanes. Electrostatic contributions dominate the preference for **7**_ax_ over **7**_eq_. Notably too, the energy difference between the **7**_ax_/**7**_eq_ conformer is greater than that found for the methoxyl derivative **5**. Stabilizing nonclassical hydrogen bonding (NCHB) CO···HC interactions makes a greater contribution in **5** than those involving fluorine in **7**, but attaining the **5**_ax_ conformer requires a significantly larger entropy penalty than for **7**_ax_, and thus globally **7** shows the higher axial preference. Additional CF_2_ groups were introduced around the cyclohexane ring and depending on their placement the cyclohexanes adopt preferred axial (*eg***9**) or equatorial (*eg***10**) conformers, influenced largely by electrostatic (NCHB) CF···HC interactions.

Theory comparisons extended to exploring the analogous chloro- and bromocyclohexanes **11**–**16** with CF_2_’s similarly placed in the cyclohexane rings. In these cases, NCHBs between CCl···HC and CBr···HC did not offer significant stabilizing interactions. Instead, it was found that the geminal hydrogens in CHX (X = Cl, Br) were more electropositive than that found for CHF, and (NCHB) CF···HCX electrostatic interactions involving fluorine to these geminal hydrogens contributed significantly to the conformer populations of **11**–**16**.

## 4 Experimental Section

### 4.1 Computational Methods

The optimization of axial and equatorial conformers for compounds **7**–**10** and **11**–**16** employed Truhlar’s hybrid meta-GGA functional M06-2X,^22^ coupled with Dunning’s correlation consistent triple-ζ basis set augmented with diffuse functions aug-cc-pVTZ.^23^ The choice of the M06-2X/aug-cc-pVTZ theoretical level was based on its proven accuracy in previous studies involving analogous fluorocyclohexanes.^10^ Harmonic frequency calculations at the same theoretical level were carried out in order to identify each geometry as a true energy minimum, showing no imaginary frequency. Thermal corrections to the electronic energy within the ideal gas-rigid rotor-harmonic oscillator model were derived from these frequency calculations, providing the ring interconversion Δ*G* energy for the equilibria of compounds **7**–**10** and **11**–**16**. NBO calculations were performed using the NBO7.0 program^18^ at the M06-2X/aug-cc-pVTZ level of theory including the NBO energetic analysis, natural Coulomb electrostatics,^24^ and natural steric analysis^25^ (LEWIS, NCE, and STERIC keywords, respectively). The Gibbs free energies in solution were determined using the M06-2X/aug-cc-pVTZ theoretical level employing the integral equation formalism variant of the polarazible continuum model (IEFPCM).^16^ All calculations were performed using Gaussian 16 Rev C.01 program.^26^

## Data Availability

The data underlying this study are available in the published article and its Supporting Information.

## Supporting Information Available

Experimental protocols for the synthesis of **7** and outlined computational methods and associated data (PDF)
