# Supplementary material for: Conformational Analysis Explores the Role of Electrostatic Nonclassical CF···HC Hydrogen Bonding Interactions in Selectively Halogenated Cyclohexanes
Source: J Org Chem. 2024 Mar 5;89(6):4009–18. doi: 10.1021/acs.joc.3c02868 (PMC10949234; doi:10.1021/acs.joc.3c02868)
Supplement: Supplementary file 1 — jo3c02868_si_001.pdf [file jo3c02868_si_001.pdf]

SUPPLEMENTARY INFORMATION

Conformational analysis explores the role of electrostatic non-classical CF···HC hydrogen bonding interactions in selectively halogenated cyclohexanes

Mengfan He,[a] Bruno A. Piscelli,[b] Rodrigo A. Cormanich, [b]* and David O'Hagan [a]*

[a] *School of Chemistry, Biomedical Sciences Research Complex, University of St Andrews, North Haugh, St Andrews, Fife KY16 9ST, United Kingdom*
[b] *Universidade Estadual de Campinas (UNICAMP), Instituto de Química, Monteiro Lobato Street, Campinas, Sao Paulo 13083-862, Brazil*

* *E-mail: do1@st-andrews.ac.uk; cormanich@unicamp.br*

Table of Contents

General 1

Synthesis protocols and NMR data 2

NMR Spectra 3

Computational details 10

References 53

General

NMR Spectra were recorded on Bruker AVIII 500, AVIII-HD 500 or AVIII-HD 700 spectrometer. NMR analyses were carried out at room temperature in indicated deuterated solvents unless otherwise noted. Chemical shift data are reported as *δ* in units of ppm relative to respective deuterated NMR solvent. Coupling constant *J* was reported in Hz. ^{1}H, ^{13}C, ^{19}F NMR spectra were recorded at 470 MHz with and without ^{1}H decoupling, relative to CCl_3F (δF = 0.00 ppm). Structural assignments were made with additional information from gCOSY, gHSQC, and gHMBC experiments in conjunction with ^{1}H, ^{13}C, and ^{19}F NMR data. Multiplicities are indicated by: s for singlet, d for doublet, t for triplet, q for quartet, p for septet and m for multiplet and br. for the broad brand. High resolution mass spectra were recorded on a Thermo Scientific Exactive Orbitrap mass spectrometer.

All reactions were carried out under an argon atmosphere with standard Schlenk techniques unless otherwise specified. The reaction glassware was flame dried or oven dried and cooled under vacuum. Commercially available chemicals were purchased from Acros, Alfa Aeser, Fisher Scientific, Fluorochem, Sigma Aldrich, Strem Chemicals, and TCI (UK) and used as received unless otherwise stated.

DCM and THF were dried and deoxygenated using an MBraun SPS-800 solvent system. Room temperature refers to the temperature range 15-25 ^{o} C. *In vacuo* refer to the use of rotary evaporator with membrane pump at 30-50 mbar Analytical thin-layer chromatography was carried out on aluminium backed Merck TLC silica gel 60 F254 plates. The instrument for photocatalysis is HCK1012-01-002 (EVOLUCHEM LED 450PF) with non-filters, PF Series light 18W 450 nm 25 degrees 100-240VAC. Reaction performed vessel is Schlenk borosilicate tube. Distance from the light source to the irradiation schlenk tube: ~ 40 mm. These plates were visualised using UV light at 254 nm wavelength, dyed by potassium permanganate or phosphomolybdic acid followed by air dryer heating. Flash column chromatography was performed with Sigma-Aldrich silica gel, 60 Å pore size and 230-400 mesh, 40-63 µm particle size under 5 psi compressed air.

Synthesis protocols and NMR data

1,1,4-trifluorocyclohexane (7)

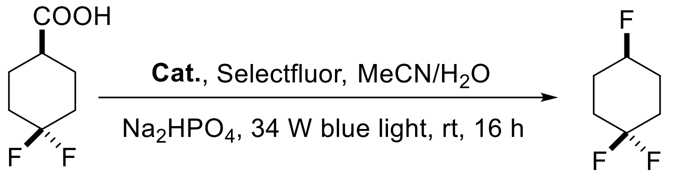

6 **7**

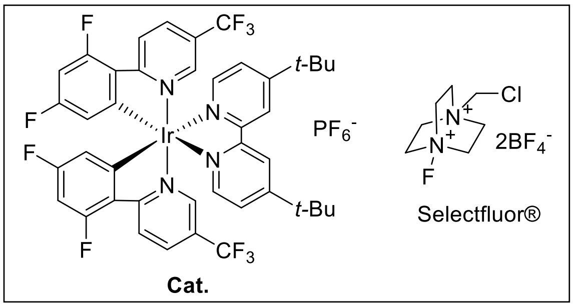

A solution of Ir[dF(CF$_3$)ppy]$_2$(dtbpy)PF$_6$ (**cat.**, 33.7 mg, 0.03 mmol, 1 mol%), 4,4-difluorocyclohexane-1-carboxylic acid **6** (492.5 mg, 3 mmol), Na_2HPO_4 (851.8 mg, 6 mmol), and Selectfluor® (3.2g, 9 mmol) in a mixture of acetonitrile/water (10 mL, 1:1 v/v) was degassed by sparging argon for 10 min, and then was irradiated with two 34 W blue LEDs (at approximately 4 cm from the light source) at room temperature overnight. After the reaction completion, the crude reaction mixture was extracted with diethyl ether (3 x 10 mL), the combined organic extracts were dried over anhydrous Na_2SO_4, and concentrated under reduced pressure to give the product as a brown oil **7** (398.2 mg, 96%). ^{1}H NMR (400 MHz, Chloroform-*d*) δ 4.89 – 4.68 (m, 1H), 2.13 – 1.70 (m, 8H). ^{13}C{^{1}H} NMR (126 MHz, Chloroform-*d*) δ 122.7 (dd, *J* = 239.3, 242.7 Hz), 87.2 (d, *J* = 170.0 Hz), 28.9 (td, *J* = 4.2, 24.9 Hz), 27.9 (ddd, *J* = 2.3, 7.6, 21.3 Hz). ^{19}F{^{1}H}NMR (470 MHz, Acetone-*d*$_6$) δ -92.7 (d, *J* = 239.8 Hz), -103.9 (d, *J* = 240.1 Hz), -187.4. HRMS (ESI) *m/z*: [M]$^+$ Calculated for $C_6H_9F_3$ 138.0651. Found 138.0651.

See Reference Ventre, S.; Petronijevic, F. R.; MacMillan, D. W. Decarboxylative Fluorination of Aliphatic Carboxylic Acids *via* Photoredox Catalysis. *J. Am. Chem. Soc.* **2015**,*137*, 5654-5657.

NMR Spectra

1,1,4-trifluorocyclohexane (7)

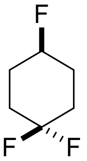

^1H NMR (400 MHz, Chloroform-*d*)

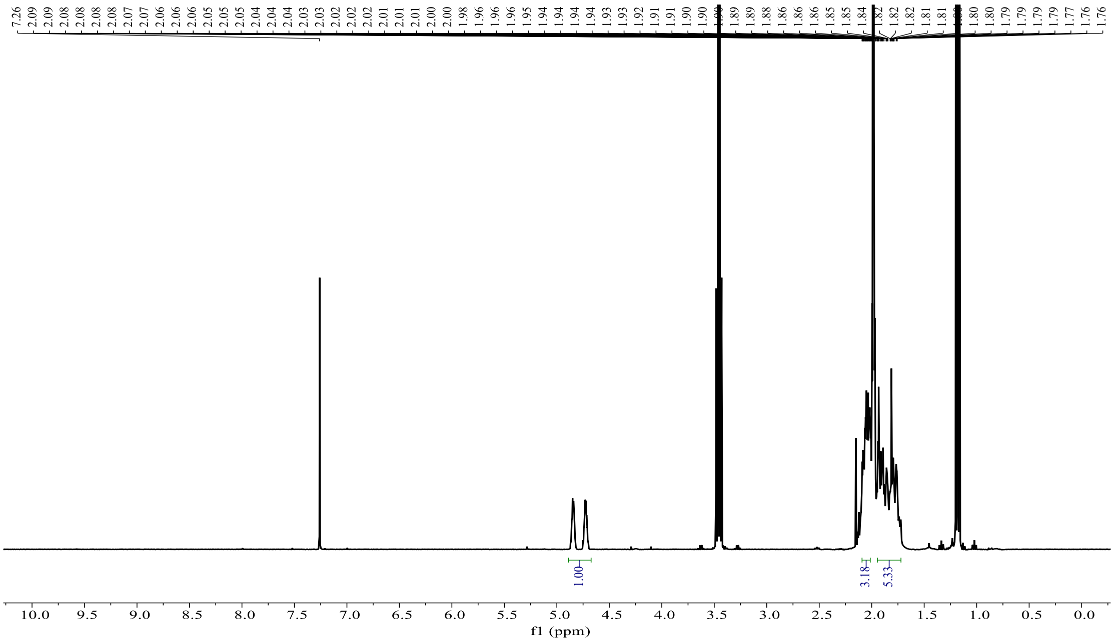

$^{13}C\{^1H\}$ NMR (126 MHz, Chloroform-*d*)

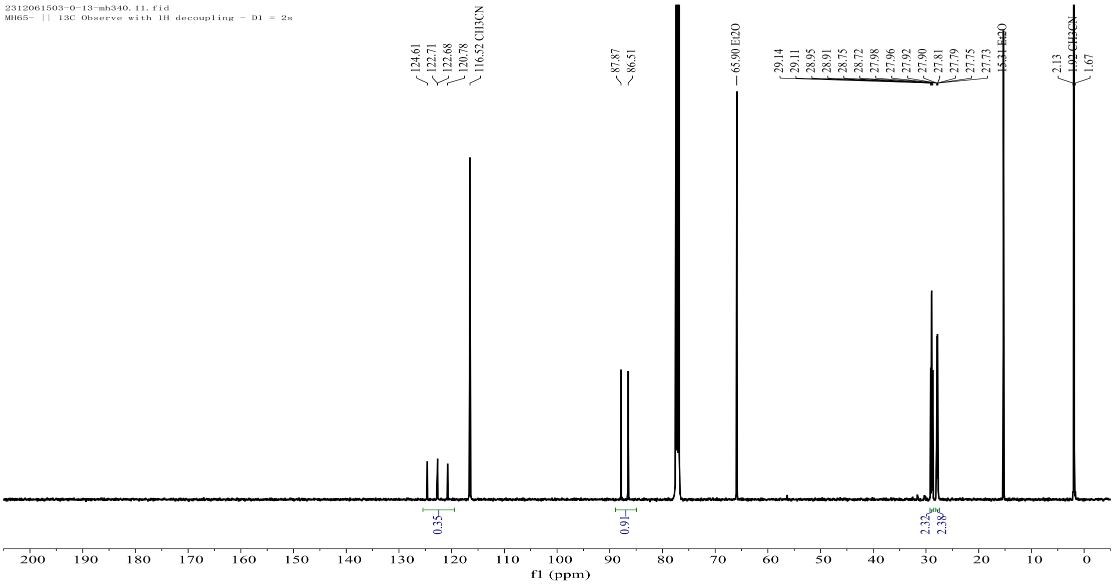

$^{19}F\{^{1}H\}$ NMR (470 MHz, Acetone-d_6)

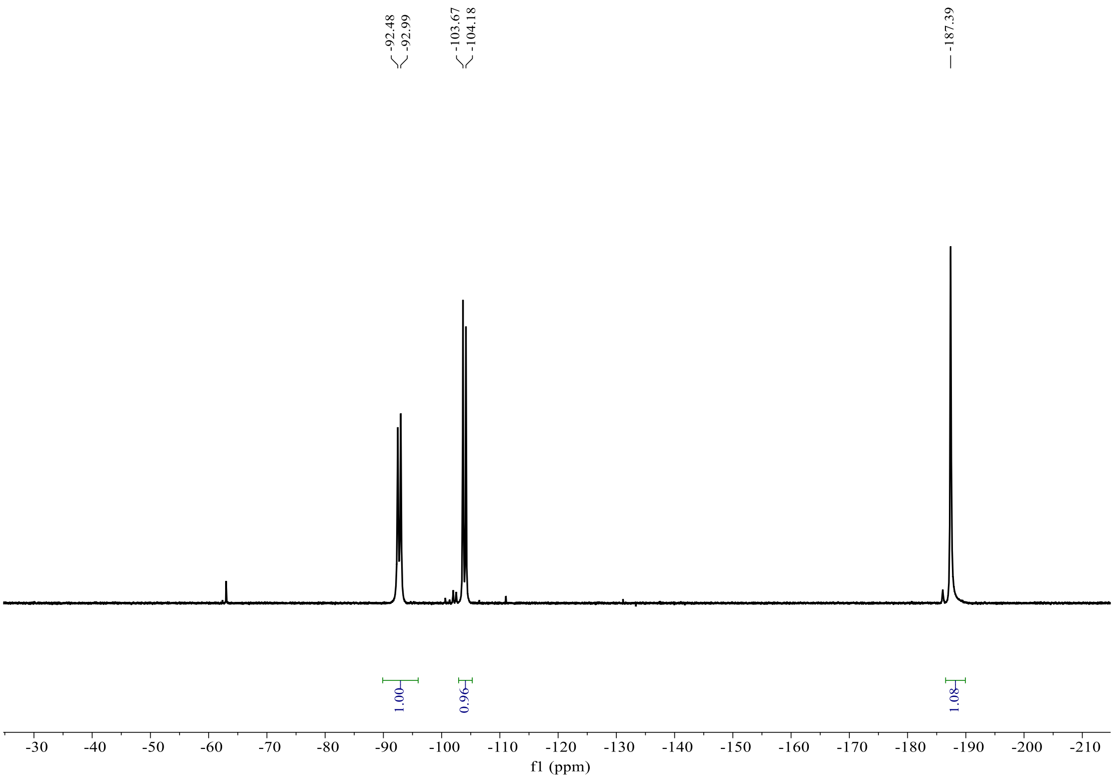

^{1}H-^{1}H-COSY (500 MHz, Chloroform-d)

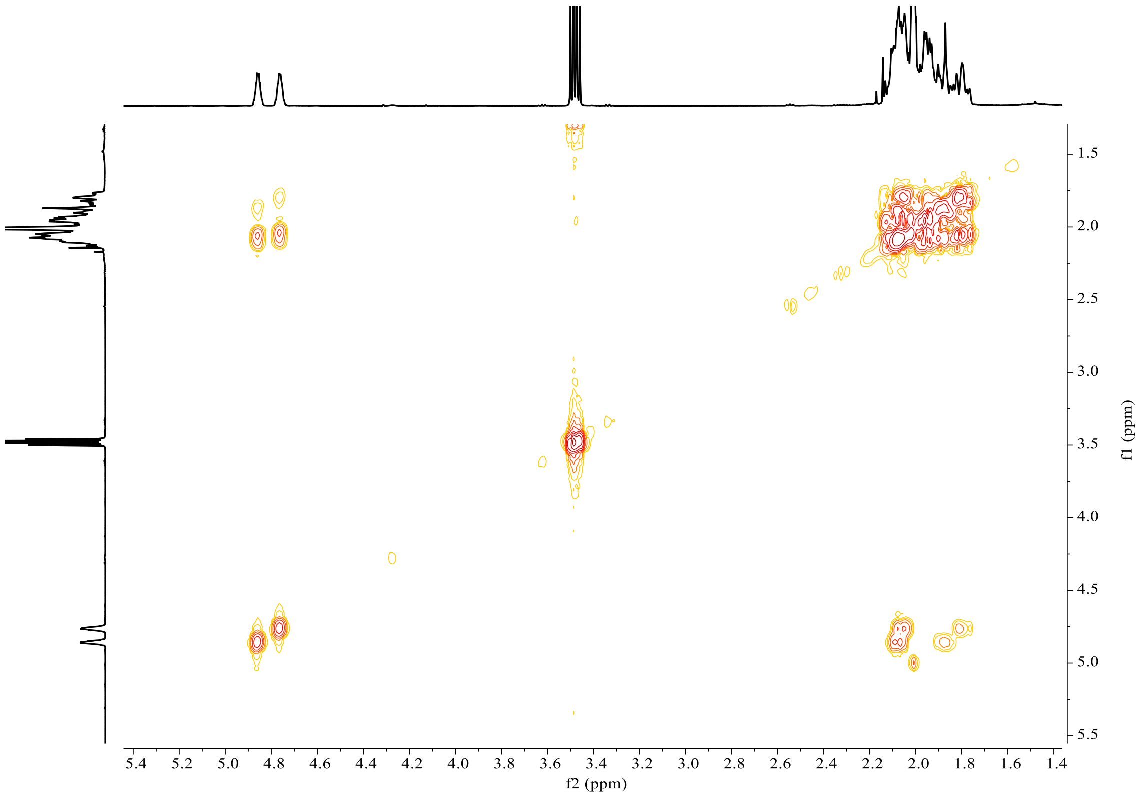

^{1}H-^{13}C-HSQC (500 MHz and 126 MHz, Chloroform-*d*)

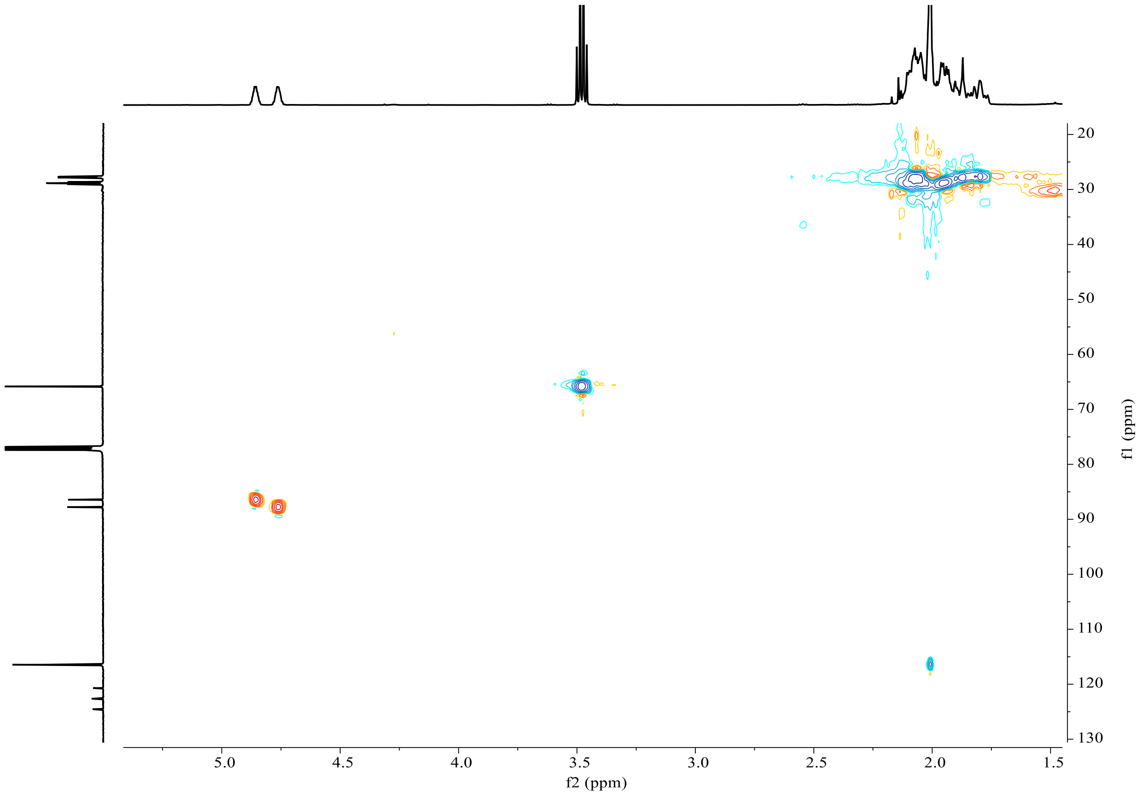

^{1}H-^{13}C-HMBC (500 MHz and 126 MHz, Chloroform-*d*)

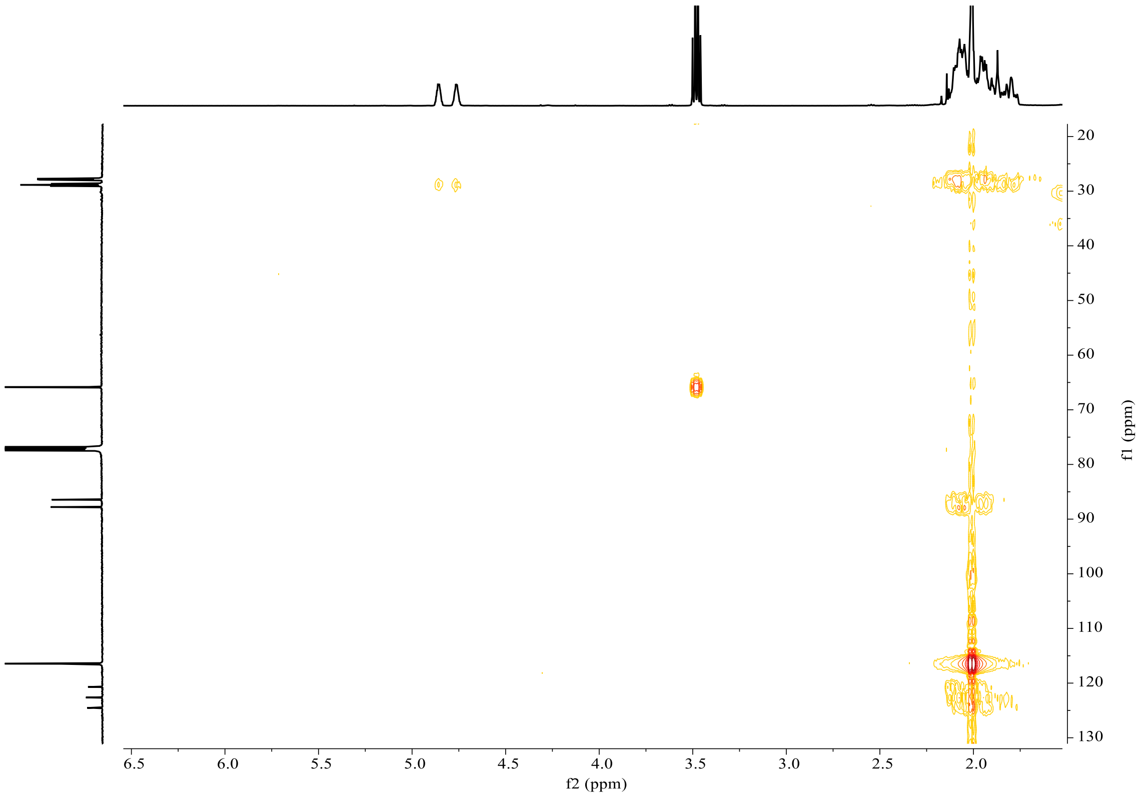

Low temperature NMR spectra

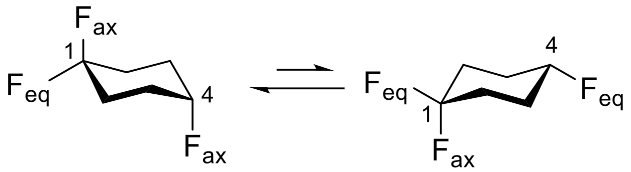

7$_{ax}$ **7**$_{eq}$

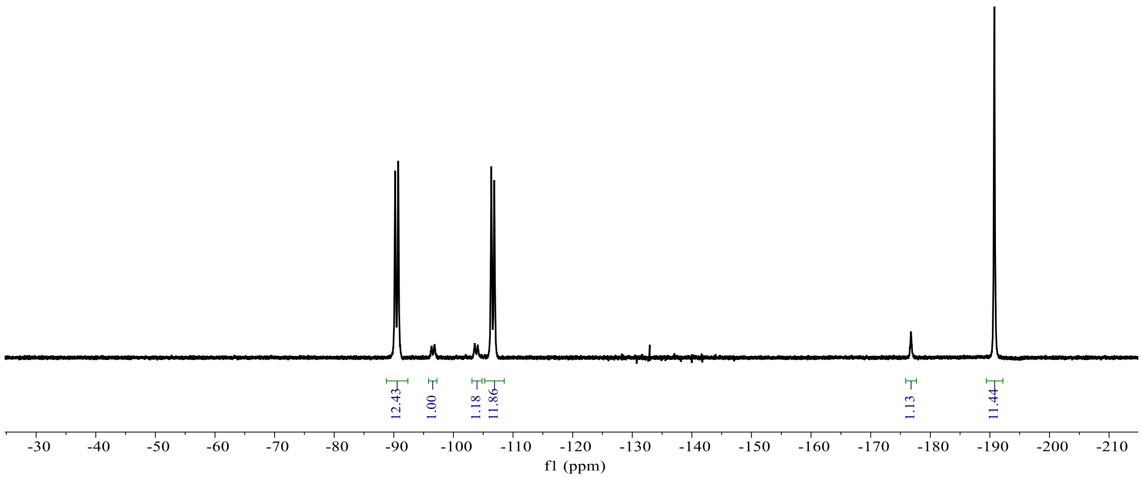

Figure S1: **7**$_{ax}$: ^{19}F{^{1}H} NMR (470 MHz, at -78 ℃, **Hexane with 10% Benzene-*d*6**) δ -90.5 (d, *J* = 239.8 Hz, 1F$_{eq}$), -106.6 (d, *J* = 240.4 Hz, 1F$_{ax}$), -190.7 (4F$_{ax}$).

7$_{eq}$: ^{19}F NMR (470 MHz, at -78 ℃, **Hexane with 10% Benzene-*d*6**) δ -96.6 (d, *J* = 241.2 Hz, 1F$_{eq}$), -103.8 (d, *J* = 241.0 Hz, 1F$_{ax}$), -176.8 (4F$_{eq}$).

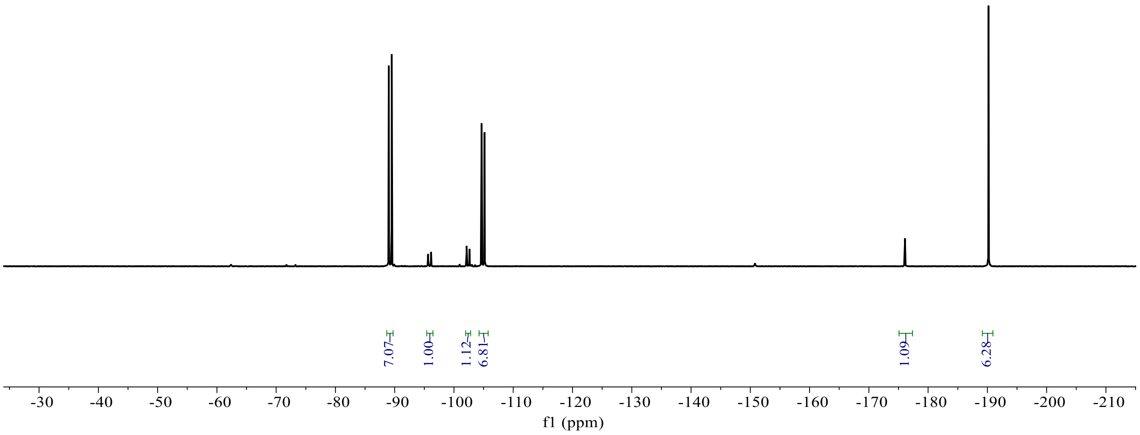

Figure S2: **7**$_{ax}$: ^{19}F{^{1}H} NMR (470 MHz, at -78℃, **Dichloromethane-*d*2**) δ -89.2 (d, *J* = 233.2 Hz, 1F$_{eq}$), -104.9 (dd, *J* = 6.0, 233.0 Hz, 1F$_{ax}$), -190.2 (4F$_{ax}$).

7$_{eq}$: ^{19}F NMR (470 MHz, at -78℃, **Dichloromethane-*d*2**) δ-95.9 (dd, *J* = 8.7, 235.5 Hz, 1F$_{eq}$), -102.4 (d, *J* = 235.6 Hz, 1F$_{ax}$), -176.1 (4F$_{eq}$).

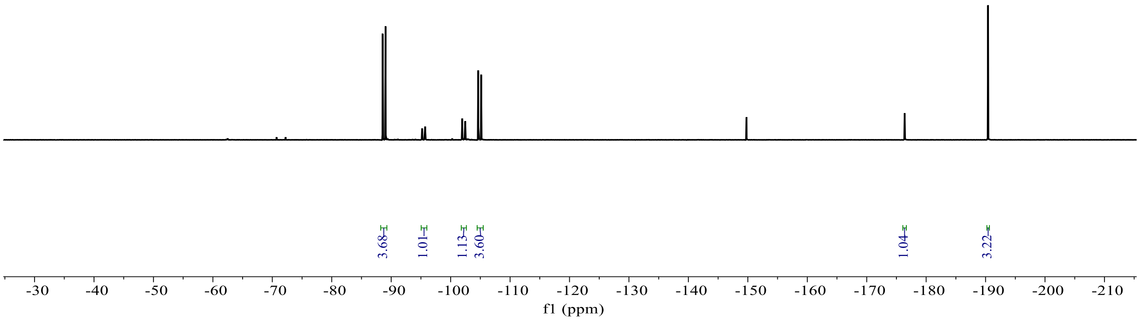

Figure S3: **7**$_{ax}$: $^{19}F\{^1H\}$ NMR (470 MHz, at -78 ℃, **Acetone-*d*$_6$**) δ -88.8 (d, *J* = 234.6 Hz, 1F_{eq}), -104.9 (dd, *J* = 5.9, 234.6 Hz, 1F_{ax}), -190.4 (4F_{ax}).

7$_{eq}$: ^{19}F NMR (470 MHz, at -78 ℃, **Acetone-*d*$_6$**) δ -95.4 (dd, *J* = 8.9, 236.0 Hz, 1F_{eq}), -102.2 (d, *J* = 236.0 Hz, 1F_{ax}), -176.4 (4F_{eq}).

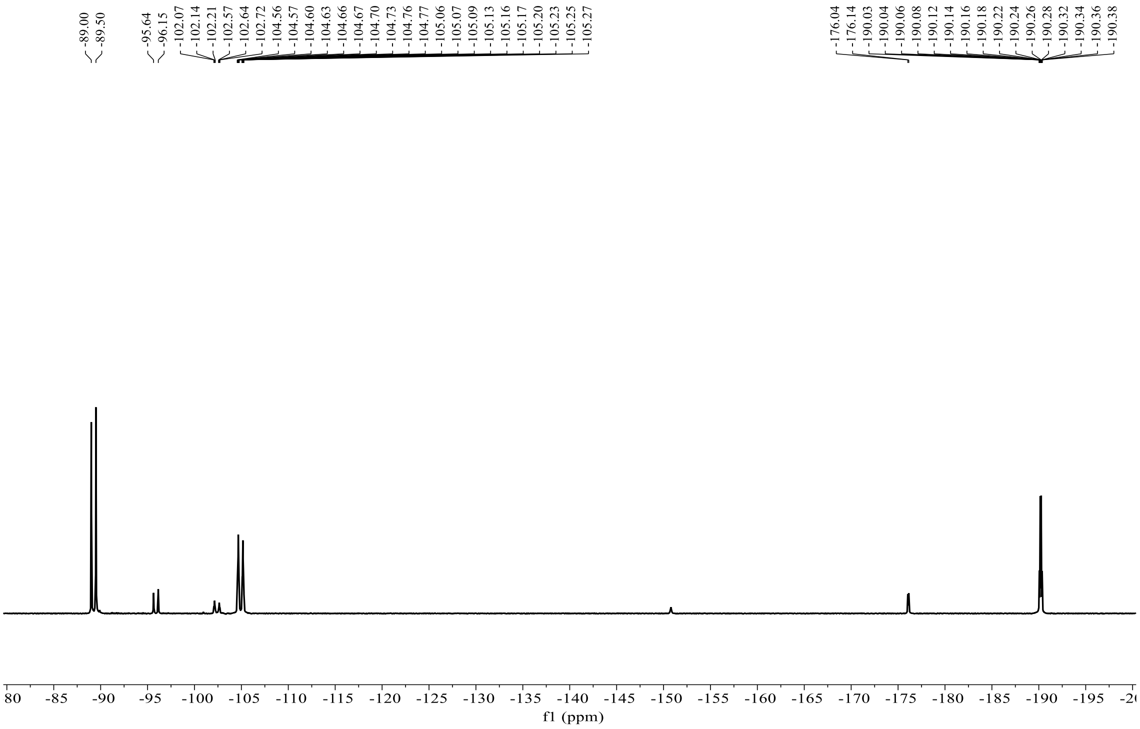

Figure S4: **7**$_{ax}$: $^{19}F\{^{1}H\}$ NMR (470 MHz, at -78 ℃, **Dichloromethane-*d*$_2$**, <u>with ^{1}H coupling</u>) δ -89.2 (d, *J* = 233.4 Hz, 1F$_{eq}$), -104.3 – -105.8 (m, 1F$_{ax}$), -190.2 (qq, *J* = 8.2, 46.6 Hz, 4F$_{ax}$).

7$_{eq}$: ^{19}F NMR (470 MHz, at -78℃, **Dichloromethane-*d*$_2$**, <u>with ^{1}H coupling</u>) δ-95.9 (d, *J* = 237.4 Hz, 1F$_{eq}$), -102.4 (dt, *J* = 34.9, 236.1 Hz, 1F$_{ax}$), -176.1 (d, *J* = 47.9 Hz, 4F$_{eq}$).

Computational details

The optimization of axial and equatorial conformers for compounds **7-10** and **11-16** employed Truhlar's hybrid meta-GGA functional M06-2X[1], coupled with Dunning's correlation consistent triple-ζ basis set augmented with diffuse functions aug-cc-pVTZ[2]. The choice of the M06-2X/aug-cc-pVTZ theoretical level was based on its proven accuracy in previous studies involving analogous fluorocyclohexanes.[3] Harmonic frequency calculations at the same theoretical level were carried out in order to identify each geometry as true energy minimum, showing no imaginary frequency. Thermal corrections to the electronic energy within the ideal gas-rigid rotor-harmonic oscillator model were derived from these frequency calculations, providing the ring interconversion ΔG energy for the equilibria of compounds **7-10** and **11-16**. Thermochemical values for compound **7** were also computed considering the VT-NMR spectra acquisition temperature of 195K. NBO calculations were performed using the NBO7.0 program[4] at the M06-2X/aug-cc-pVTZ level of theory including the NBO Energetic Analysis, Natural Coulomb Electrostatics[5] and Natural Steric Analysis[6] (LEWIS, NCE and STERIC keywords, respectively). The Gibbs free energies in solution were determined using the M06-2X/aug-cc-pVTZ theoretical level employing the integral equation formalism variant of the polarazible continuum model (IEFPCM)[7]. All calculations were performed using Gaussian 16 Rev C.01 program[8].

Table S1. Gas phase calculated total relative energy (ΔE), total relative enthalpy energy (ΔH) and total relative Gibbs free energy (ΔG) obtained at the M06-2X/aug-cc-pVTZ theoretical level for compounds **5** and **7-16**, in kcal mol^{-1}. The Δ energies are considered as (*ax-eq*), thus negative energy values represent axial preference, and the positive ones equatorial preference.

Compound	Δ*E*	Δ*H*	Δ*G*
**5**[a]	-1.08	-1.00	-0.74
**7**	-1.10	-1.09	-1.06
**8**	+0.08	+0.10	+0.12
**9**	-2.81	-2.89	-2.73
**10**	+2.16	+2.09	+2.00
**11**	-0.93	-0.90	-0.70
**12**	-0.94	-0.91	-0.67
**13**	-1.66	-1.71	-1.41
**14**	-1.23	-1.29	-0.92
**15**	+3.64	+3.56	+3.70
**16**	+3.87	+3.80	+4.04

[a]*Values obtained from ref.*[3b]

Table S2. NBO analysis relative energies (in kcal mol^{-1}) obtained at M06-2X/aug-cc-pVTZ theoretical level for compounds **5** and **7-16**, where ΔE is the electronic, ΔE_L the Lewis, ΔE_{NL} the non-Lewis, ΔE_{NCE} the electrostatic and ΔE_{NSA} the steric energies. The Δ energies are considered as (*ax-eq*), thus negative energy values represent axial preference, and the positive ones equatorial preference.

**Compound**	ΔE	ΔE_L	(hyperconjugation) ΔE_{NL}	(electrostatics) ΔE_{NCE}	(sterics) ΔE_{NSA}
**5**[a]	-1.08	-3.73	+2.65	-3.50	+3.38
**7**	-1.10	-2.51	+1.41	-3.24	+1.93
**8**	+0.08	-1.53	+1.61	+3.76	+2.00
**9**	-2.81	-11.21	+8.39	-20.29	+1.01
**10**	+2.16	+5.82	-3.67	+5.16	-0.75
**11**	-0.93	-1.11	+0.18	-5.81	+2.58
**12**	-0.94	-0.39	-0.56	-6.14	+2.69
**13**	-1.66	-4.33	+2.66	-10.38	+2.18
**14**	-1.23	-2.25	+1.02	-8.00	+3.28
**15**	+3.64	+5.05	-1.40	+3.21	+0.28
**16**	+3.87	+4.65	-0.78	+2.94	+0.73

[a]*Values obtained from ref.*[3b]

Table S3. Total relative Gibbs free energy (Δ*G*) obtained at the M06-2X/aug-cc-pVTZ theoretical level for compounds **7-16**, in kcal mol^{-1}, considering implicit solvation. The Δ energies are considered as (*ax-eq*), thus negative energy values represent axial preference, and the positive ones equatorial preference.

Compound	Gas-Phase	Hexane	DCM	Acetone
**7**	-1.06	-0.90	-0.65	-0.63
**8**	+0.12	+0.22	+0.33	+0.35
**9**	-2.73	-2.08	-0.94	-0.67
**10**	+2.00	+1.61	+0.34	+0.03
**11**	-0.70	-0.54	-0.26	-0.25
**12**	-0.67	-0.48	-0.22	-0.22
**13**	-1.41	-0.81	0.28	0.55
**14**	-0.92	-0.25	0.67	0.99
**15**	+3.70	+3.70	+2.21	+1.84
**16**	+4.04	+3.67	+2.68	+2.30

Table S4. Individual interaction energies in **5** and **7-16** contributing to the global ΔE_{NL}, ΔE_{NCE} and ΔE_{NSA} obtained at the M06-2X/aug-cc-pVTZ theoretical level, in kcal mol^{-1}.

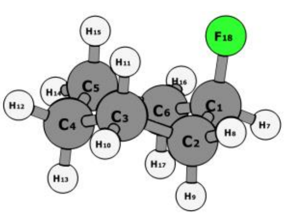

Axial

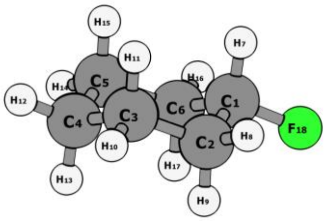

Equatorial

	ΔE_{NL} (hyperconjugation)	ΔE_{NCE} (electrostatics)	ΔE_{NSA} (sterics)
**8ax**	$\sigma_{C2H9} \rightarrow \sigma^*_{C1F}$ = 5.88<br>$\sigma_{C2H9} \rightarrow \sigma^*_{C1H}$ = <0.5	CH···OC NCHB<br>-10.5	σ_{C1F18} / σ_{C2H8} = 0.83<br>σ_{C1F18} / σ_{C2H9} = 3.93
**8eq**	$\sigma_{C2H9} \rightarrow \sigma^*_{C1F}$ = 1.02<br>$\sigma_{C2H9} \rightarrow \sigma^*_{C1H}$ = 3.38	$C_1H_7 \cdots H_{11}C_3$<br>+3.7	σ_{C1F18} / σ_{C2H8} = 0.69<br>σ_{C1F18} / σ_{C2H9} = 0.83

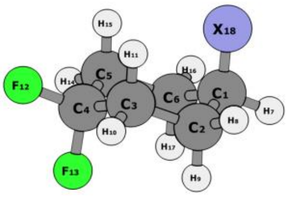

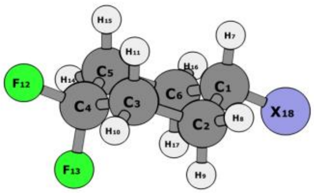

Axial **X = OMe(5), F(7), Cl(11), Br(12)** **Equatorial**

	ΔE_{NL} (hyperconjugation)	ΔE_{NCE} (electrostatics)	ΔE_{NSA} (sterics)
**5ax**	$\sigma_{C2H9} \rightarrow \sigma^*_{C1O}$ = 5.53<br>$\sigma_{C2H9} \rightarrow \sigma^*_{C1H}$ = <0.5	CH···OC NCHB<br>-16.8	σ_{C1O18} / σ_{C2H8} = 0.57<br>σ_{C1O18} / σ_{C2H9} = 4.29
**5eq**	$\sigma_{C2H9} \rightarrow \sigma^*_{C1O}$ = 0.82<br>$\sigma_{C2H9} \rightarrow \sigma^*_{C1H}$ = 3.63	$C_1H_7 \cdots H_{11}C_3$<br>+4.1	σ_{C1O18} / σ_{C2H8} = 0.61<br>σ_{C1O18} / σ_{C2H9} = 0.80
**7ax**	$\sigma_{C2H9} \rightarrow \sigma^*_{C1F}$ = 5.81<br>$\sigma_{C2H9} \rightarrow \sigma^*_{C1H}$ = <0.5	CH···FC NCHB<br>-11.5	σ_{C1F18} / σ_{C2H8} = 0.77<br>σ_{C1F18} / σ_{C2H9} = 4.03
**7eq**	$\sigma_{C2H9} \rightarrow \sigma^*_{C1F}$ = 1.02<br>$\sigma_{C2H9} \rightarrow \sigma^*_{C1H}$ = 3.30	$C_1H_7 \cdots H_{11}C_3$<br>+4.1	σ_{C1F18} / σ_{C2H8} = 0.66<br>σ_{C1F18} / σ_{C2H9} = 0.79
**11ax**	$\sigma_{C2H9} \rightarrow \sigma^*_{C1Cl}$ = 7.16<br>$\sigma_{C2H9} \rightarrow \sigma^*_{C1H}$ = <0.5	$C_3H_{11} \cdots Cl_{18}C_1$<br>-2.7	σ_{C1Cl18} / σ_{C2H8} = 1.43<br>σ_{C1Cl18} / σ_{C2H9} = 5.44
**11eq**	$\sigma_{C2H9} \rightarrow \sigma^*_{C1Cl}$ = <0.5<br>$\sigma_{C2H9} \rightarrow \sigma^*_{C1H}$ = 3.79	$C_1H_7 \cdots H_{11}C_3$<br>+5.4	σ_{C1Cl18} / σ_{C2H8} = 1.27<br>σ_{C1Cl18} / σ_{C2H9} = 1.25
**12ax**	$\sigma_{C2H9} \rightarrow \sigma^*_{C1Br}$ = 8.01<br>$\sigma_{C2H9} \rightarrow \sigma^*_{C1H}$ = <0.5	$C_3H_{11} \cdots Br_{18}C_1$<br>-1.2	σ_{C1Br18} / σ_{C2H8} = 1.69<br>σ_{C1Br18} / σ_{C2H9} = 5.83
**12eq**	$\sigma_{C2H9} \rightarrow \sigma^*_{C1Br}$ = <0.5<br>$\sigma_{C2H9} \rightarrow \sigma^*_{C1H}$ = 3.91	$C_1H_7 \cdots H_{11}C_3$<br>+5.5	σ_{C1Br18} / σ_{C2H8} = 1.48<br>σ_{C1Br18} / σ_{C2H9} = 1.45

Table S4. Continuation.

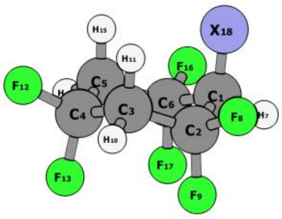

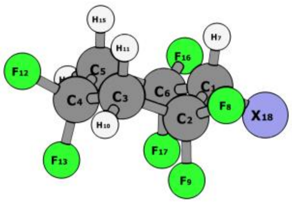

Axial **X = F(9), Cl(13), Br(14)** **Equatorial**

	ΔE_{NL} (hyperconjugation)	ΔE_{NCE} (electrostatics)	ΔE_{NSA} (sterics)
**9ax**	$\sigma_{C2F9} \rightarrow \sigma^*_{C1F}$ = 1.14<br>$\sigma_{C2F9} \rightarrow \sigma^*_{C1H}$ = <0.5	CH···FC NCHB<br>-11.3	σ_{C1F18} / σ_{C2F8} = <0.5<br>σ_{C1F18} / σ_{C2F9} = 2.17
**9eq**	$\sigma_{C2F9} \rightarrow \sigma^*_{C1F}$ = <0.5<br>$\sigma_{C2F9} \rightarrow \sigma^*_{C1H}$ = 0.76	$C_1H_7 \cdots H_{11}C_3$<br>+5.7	σ_{C1F18} / σ_{C2F8} = <0.5<br>σ_{C1F18} / σ_{C2F9} = <0.5
**13ax**	$\sigma_{C2F9} \rightarrow \sigma^*_{C1Cl}$ = 1.75<br>$\sigma_{C2F9} \rightarrow \sigma^*_{C1H}$ = <0.5	$C_3H_{11} \cdots Cl_{18}C_1$<br>-0.4	σ_{C1Cl18} / σ_{C2F8} = 0.70<br>σ_{C1Cl18} / σ_{C2F9} = 3.41
**13eq**	$\sigma_{C2F9} \rightarrow \sigma^*_{C1Cl}$ = <0.5<br>$\sigma_{C2F9} \rightarrow \sigma^*_{C1H}$ = 0.88	$C_1H_7 \cdots H_{11}C_3$<br>+7.2	σ_{C1Cl18} / σ_{C2F8} = <0.5<br>σ_{C1Cl18} / σ_{C2F9} = 0.67
**14ax**	$\sigma_{C2F9} \rightarrow \sigma^*_{C1Br}$ = 2.06<br>$\sigma_{C2F9} \rightarrow \sigma^*_{C1H}$ = <0.5	$C_3H_{11} \cdots Br_{18}C_1$<br>+1.8	σ_{C1Br18} / σ_{C2F8} = 0.83<br>σ_{C1Br18} / σ_{C2F9} = 4.02
**14eq**	$\sigma_{C2F9} \rightarrow \sigma^*_{C1Br}$ = <0.5<br>$\sigma_{C2F9} \rightarrow \sigma^*_{C1H}$ = 0.90	$C_1H_7 \cdots H_{11}C_3$<br>+7.4	σ_{C1Br18} / σ_{C2F8} = 0.60<br>σ_{C1Br18} / σ_{C2F9} = 0.80

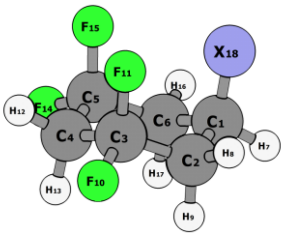

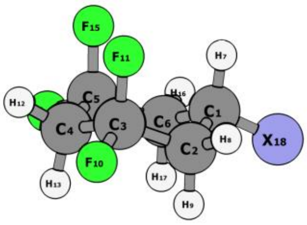

Axial **X = F(10), Cl(15), Br(16)** **Equatorial**

	ΔE_{NL} (hyperconjugation)	ΔE_{NCE} (electrostatics)	ΔE_{NSA} (sterics)
**10ax**	$\sigma_{C2H9} \rightarrow \sigma^*_{C1F}$ = 5.31<br>$\sigma_{C2H9} \rightarrow \sigma^*_{C1H}$ = <0.5	$C_3F_{11} \cdots F_{18}C_1$<br>+16.1	σ_{C1F18} / σ_{C2H8} = 1.07<br>σ_{C1F18} / σ_{C2H9} = 3.61
**10eq**	$\sigma_{C2H9} \rightarrow \sigma^*_{C1F}$ = 1.11<br>$\sigma_{C2H9} \rightarrow \sigma^*_{C1H}$ = 2.91	CF···HC NCHB<br>-8.4	σ_{C1F18} / σ_{C2H8} = 0.53<br>σ_{C1F18} / σ_{C2H9} = 0.84
**15ax**	$\sigma_{C2H9} \rightarrow \sigma^*_{C1Cl}$ = 6.30<br>$\sigma_{C2H9} \rightarrow \sigma^*_{C1H}$ = 0.63	$C_3F_{11} \cdots Cl_{18}C_1$<br>+2.1	σ_{C1Cl18} / σ_{C2H8} = 1.92<br>σ_{C1Cl18} / σ_{C2H9} = 4.96
**15eq**	$\sigma_{C2H9} \rightarrow \sigma^*_{C1Cl}$ = 3.38<br>$\sigma_{C2H9} \rightarrow \sigma^*_{C1H}$ = <0.5	CF···HC NCHB<br>-10.6	σ_{C1Cl18} / σ_{C2H8} = 1.08<br>σ_{C1Cl18} / σ_{C2H9} = 1.28
**16ax**	$\sigma_{C2H9} \rightarrow \sigma^*_{C1Br}$ = 6.92<br>$\sigma_{C2H9} \rightarrow \sigma^*_{C1H}$ = 0.66	$C_3F_{11} \cdots Br_{18}C_1$<br>-0.6	σ_{C1Br18} / σ_{C2H8} = 2.18<br>σ_{C1Br18} / σ_{C2H9} = 5.34
**16eq**	$\sigma_{C2H9} \rightarrow \sigma^*_{C1Br}$ = <0.5<br>$\sigma_{C2H9} \rightarrow \sigma^*_{C1H}$ = 3.48	CF···HC NCHB<br>-10.7	σ_{C1Br18} / σ_{C2H8} = 1.25<br>σ_{C1Br18} / σ_{C2H9} = 1.47

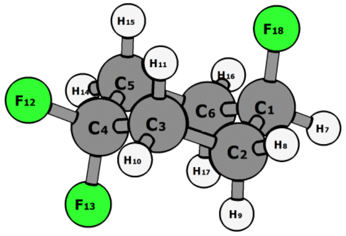

$\mathbf{7}_{ax}$

LP(F18) $\rightarrow \sigma^*_{C1H7}$ = 7.86

$\sigma_{C2H9} \rightarrow \sigma^*_{C1F18}$ = 5.81
$\sigma_{C1F18} \rightarrow \sigma^*_{C2H9}$ = 1.07

$\sigma_{C2H9} \rightarrow \sigma^*_{C1H7}$ = <0.5
$\sigma_{C1H7} \rightarrow \sigma^*_{C2H9}$ = <0.5

$\sigma_{C2C3} \rightarrow \sigma^*_{C1H7}$ = 2.01
$\sigma_{C1H7} \rightarrow \sigma^*_{C2C3}$ = 3.64

Total: 20.4

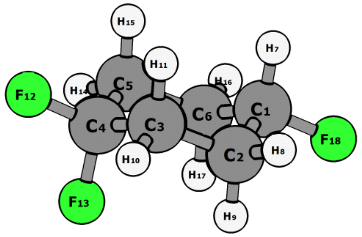

$\mathbf{7}_{eq}$

LP(F18) $\rightarrow \sigma^*_{C1H7}$ = 8.55

$\sigma_{C2H9} \rightarrow \sigma^*_{C1F18}$ = 1.02
$\sigma_{C1F18} \rightarrow \sigma^*_{C2H9}$ = <0.5

$\sigma_{C2H9} \rightarrow \sigma^*_{C1H7}$ = 3.30
$\sigma_{C1H7} \rightarrow \sigma^*_{C2H9}$ = 3.16

$\sigma_{C2C3} \rightarrow \sigma^*_{C1F18}$ = 3.64
$\sigma_{C1F18} \rightarrow \sigma^*_{C2C3}$ = 1.14

Total: 20.8

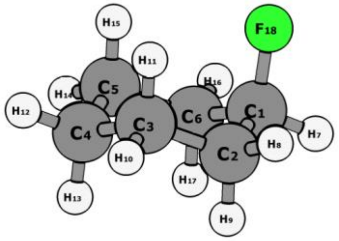

$\mathbf{8}_{ax}$

LP(F18) $\rightarrow \sigma^*_{C1H7}$ = 7.81

$\sigma_{C2H9} \rightarrow \sigma^*_{C1F18}$ = 5.88
$\sigma_{C1F18} \rightarrow \sigma^*_{C2H9}$ = 1.11

$\sigma_{C2H9} \rightarrow \sigma^*_{C1H7}$ = <0.5
$\sigma_{C1H7} \rightarrow \sigma^*_{C2H9}$ = <0.5

$\sigma_{C2C3} \rightarrow \sigma^*_{C1H7}$ = 2.04
$\sigma_{C1H7} \rightarrow \sigma^*_{C2C3}$ = 3.46

Total: 20.3

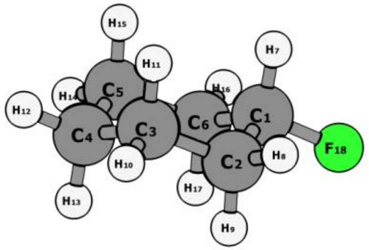

$\mathbf{8}_{eq}$

LP(F18) $\rightarrow \sigma^*_{C1H7}$ = 8.35

$\sigma_{C2H9} \rightarrow \sigma^*_{C1F18}$ = 1.02
$\sigma_{C1F18} \rightarrow \sigma^*_{C2H9}$ = <0.5

$\sigma_{C2H9} \rightarrow \sigma^*_{C1H7}$ = 3.38
$\sigma_{C1H7} \rightarrow \sigma^*_{C2H9}$ = 3.35

$\sigma_{C2C3} \rightarrow \sigma^*_{C1F18}$ = 3.84
$\sigma_{C1F18} \rightarrow \sigma^*_{C2C3}$ = 1.11

Total: 21.1

Figure S5. Hyperconjugative interactions (in kcal mol^{-1}) that lead to overall equatorial stabilization in compounds **7** and **8**. Calculated at the M06-2X/aug-cc-pVTZ theoretical level.

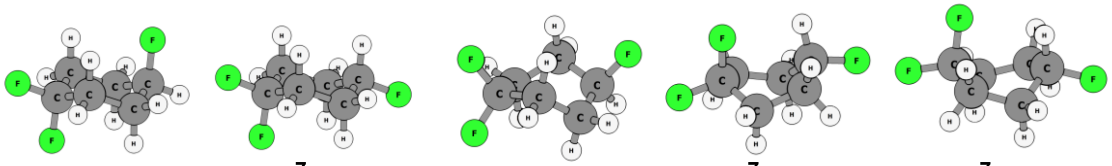

ΔG / Population	7_{ax}	7_{eq}	7_{tb1}	7_{tb2}	7_{tb3}
**Gas-Phase** (ε = **1.00)**	0.00 / 93.9%	1.06 / 6.1%	5.30 / 0.0%	6.24 / 0.0%	6.82 / 0.0%
**Hexane** (ε = **1.88)**	0.00 / 90.9%	0.89 / 9.1%	5.25 / 0.0%	6.25 / 0.0%	-
**DCM** (ε = **8.93)**	0.00 / 84.3%	0.65 / 15.7%	5.11 / 0.0%	6.15 / 0.0%	-
**Acetone** (ε = **20.49)**	0.00 / 83.6%	0.63 / 16.4%	5.05 / 0.0%	6.10 / 0.0%	-

Figure S6. Calculated relative Gibbs free energies (Δ*G*) in kcal mol^{-1} and populations in percentage for chair and twist boat conformers of **7** in gas-phase and implicit solvents (hexane, dichloromethane and acetone), at M06-2X/aug-cc-pVTZ/IEFPCM level of theory.

Table S5. Selected 3-atom angles (degrees) from the gas-phase optimized structures of unsubstituted cyclohexane and compounds **7-16**, obtained at the M06-2X/aug-cc-pVTZ theoretical level.

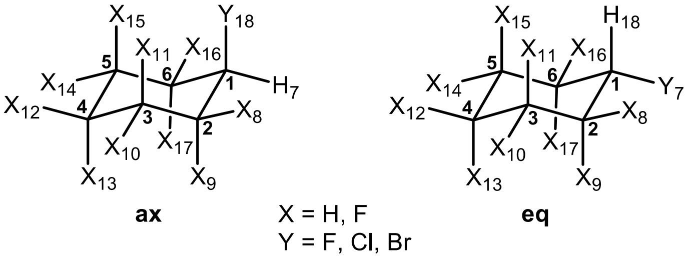

	Angle (∠)							
	**1-2-3**	**2-1-6**	**2-1-7**	**2-1-18**	**2-3-4**	**3-4-5**	**7-1-18**	**Average**
**Cyclohexane**	111.1	111.1	110.4	109	111.1	111.1	106.9	110.1
$\mathbf{7}_{ax}$	111.3	112.5	110.7	108.2	110.4	113.8	106.4	110.5
$\mathbf{7}_{eq}$	110.3	111.7	109.0	110.1	110.5	113.9	106.7	110.3
$\mathbf{8}_{ax}$	111.5	112.6	110.8	108.1	111.0	110.7	106.1	110.1
$\mathbf{8}_{eq}$	110.3	112.0	109.1	110.0	111.1	111.0	106.5	110.0
$\mathbf{9}_{ax}$	113.0	112.0	111.3	110.4	107.3	114.2	109.9	111.2
$\mathbf{9}_{eq}$	112.7	112.2	111.7	109.8	108.2	113.8	109.0	111.1
$\mathbf{10}_{ax}$	112.9	112.8	114.0	109.3	109.3	111.9	106.7	111.0
$\mathbf{10}_{eq}$	110.3	111.4	114.1	108.5	110.5	111.7	107.4	110.6
$\mathbf{11}_{ax}$	112.2	111.9	110.4	109.8	110.4	113.9	104.4	110.4
$\mathbf{11}_{eq}$	110.1	111.6	109.8	110.1	110.6	113.8	105.2	110.2
$\mathbf{12}_{ax}$	112.5	112.0	110.7	110.0	110.4	114.0	103.0	110.4
$\mathbf{12}_{eq}$	109.9	111.8	109.8	110.4	110.6	113.8	104.2	110.1
$\mathbf{13}_{ax}$	113.9	110.5	109.2	109.6	112.0	114.3	108.5	111.1
$\mathbf{13}_{eq}$	112.6	111.6	111.2	107.6	112.3	113.7	107.7	111.0
$\mathbf{14}_{ax}$	114.1	110.5	109.2	110.1	112.1	114.3	107.6	111.1
$\mathbf{14}_{eq}$	112.5	111.7	111.5	107.6	112.3	113.7	106.7	110.9
$\mathbf{15}_{ax}$	113.6	112.1	108.8	111.3	114.0	112.1	104.3	110.9
$\mathbf{15}_{eq}$	110.0	111.3	109.6	110.2	114.1	111.6	105.9	110.4
$\mathbf{16}_{ax}$	113.7	112.2	109.0	111.7	114.0	112.1	102.8	110.8
$\mathbf{16}_{eq}$	109.8	111.4	109.6	110.5	114.1	111.6	105.0	110.3

Table S6. Selected 4-atom dihedrals (degrees) from the gas-phase optimized structures of unsubstituted cyclohexane and compounds **7-16**, obtained at the M06-2X/aug-cc-pVTZ theoretical level.

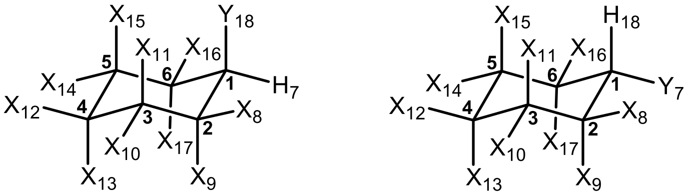

ax X = H, F **eq**
Y = F, Cl, Br

	Dihedral (ω)							
	**1-2-3-4**	**2-3-4-5**	**3-4-5-6**	**7-1-2-3**	**7-1-2-9**	**18-1-2-3**	**18-1-2-9**	**Average**
**Cyclohexane**	55.7	-55.7	55.7	-178.5	-58.3	64.4	-175.4	-41.7
**7**$_{ax}$	53.5	-55.0	55.0	-178.9	-58.0	64.9	-174.2	-41.8
**7**$_{eq}$	54.5	-53.8	53.8	-178.6	-58.1	64.7	-174.9	-41.8
**8**$_{ax}$	54.8	-56.8	56.7	-177.9	-57.2	66.3	-173.0	-41.0
**8**$_{eq}$	55.9	-55.6	55.7	-178.0	-57.8	65.5	-174.3	-41.2
**9**$_{ax}$	51.4	-50.3	50.2	-175.7	-52.5	64.4	-172.3	-40.7
**9**$_{eq}$	51.4	-50.5	50.5	-174.6	-51.1	66.4	-170.1	-39.7
**10**$_{ax}$	50.3	-49.8	49.8	-171.5	-52.0	72.1	-168.4	-38.5
**10**$_{eq}$	53.3	-48.9	48.9	-176.4	-57.1	66.1	-174.5	-41.2
**11**$_{ax}$	53.1	-54.7	54.7	-176.6	-56.7	68.9	-171.2	-40.4
**11**$_{eq}$	54.7	-53.9	53.9	-179.9	-59.7	64.5	-175.1	-42.2
**12**$_{ax}$	52.7	-54.6	54.6	-176.6	-57.0	70.1	-170.3	-40.2
**12**$_{eq}$	54.7	-54.0	54.0	-179.6	-60.1	65.2	-174.6	-42.1
**13**$_{ax}$	51.0	-49.7	49.7	-171.9	-49.5	69.4	-168.2	-38.5
**13**$_{eq}$	51.6	-50.6	50.6	-177.5	-54.3	64.9	-171.9	-41.0
**14**$_{ax}$	50.8	-49.6	49.6	-171.4	-49.2	70.7	-167.1	-38.0
**14**$_{eq}$	51.6	-50.6	50.6	-178.2	-55.0	65.2	-171.7	-41.2
**15**$_{ax}$	49.9	-49.5	49.5	-169.1	-50.7	76.6	-165.1	-36.9
**15**$_{eq}$	53.6	-49.0	49.0	-178.8	-59.5	65.0	-175.6	-42.2
**16**$_{ax}$	49.7	-49.4	49.4	-169.2	-51.2	77.9	-164.1	-36.7
**16**$_{eq}$	53.6	-49.0	49.0	-179.3	-60.1	65.4	-175.3	-42.2

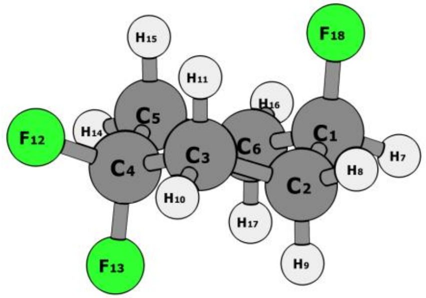

Atom Numbering

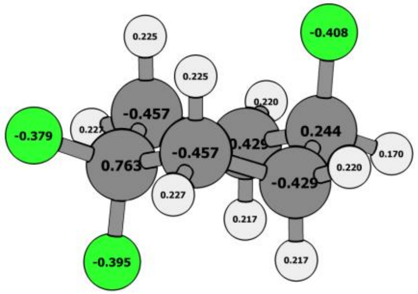

NPA charges

Table S7. Atom-atom electrostatic interactions (kcal mol^{-1}) obtained at M06-2X/aug-cc-pVTZ level using NPA charges for 7_{ax}.

	C1	C2	C3	C4	C5	C6	H7	H8	H9	H10	H11	F12	F13	H14	H15	H16	H17
**C2**	-22.84	-	-	-	-	-	-	-	-	-	-	-	-	-	-	-	-
**C3**	-14.70	42.59	-	-	-	-	-	-	-	-	-	-	-	-	-	-	-
**C4**	21.27	-43.51	-76.68	-	-	-	-	-	-	-	-	-	-	-	-	-	-
**C5**	-14.71	22.03	27.44	-76.68	-	-	-	-	-	-	-	-	-	-	-	-	-
**C6**	-22.83	24.16	22.03	-43.50	42.58	-	-	-	-	-	-	-	-	-	-	-	-
**H7**	12.59	-11.19	-7.43	11.02	-7.43	-11.19	-	-	-	-	-	-	-	-	-	-	-
**H8**	8.29	-28.69	-15.42	16.16	-8.48	-9.04	4.99	-	-	-	-	-	-	-	-	-	-
**H9**	8.26	-28.34	-15.28	19.73	-9.81	-11.05	4.99	9.00	-	-	-	-	-	-	-	-	-
**H10**	5.29	-14.81	-31.59	27.04	-9.98	-8.21	2.97	6.49	6.60	-	-	-	-	-	-	-	-
**H11**	6.49	-14.82	-31.38	27.00	-12.25	-9.55	3.34	6.63	5.31	9.60	-	-	-	-	-	-	-
**F12**	-7.38	14.61	24.47	-70.33	24.47	14.60	-4.11	-6.14	-6.78	-11.01	-11.04	-	-	-	-	-	-
**F13**	-9.23	19.34	25.54	-72.74	25.54	19.34	-5.25	-7.38	-10.76	-11.63	-9.04	22.78	-	-	-	-	-
**H14**	5.29	-8.21	-9.98	27.04	-31.59	-14.81	2.97	3.34	3.93	4.03	4.50	-11.01	-11.63	-	-	-	-
**H15**	6.49	-9.55	-12.25	27.00	-31.38	-14.82	3.34	3.95	4.03	4.50	6.46	-11.04	-9.04	9.60	-	-	-
**H16**	8.29	-9.04	-8.48	16.16	-15.42	-28.69	4.99	3.76	4.19	3.34	3.95	-6.14	-7.38	6.49	6.63	-	-
**H17**	8.26	-11.05	-9.81	19.73	-15.28	-28.34	4.99	4.19	5.96	3.93	4.03	-6.78	-10.76	6.60	5.31	9.00	-
**F18**	-23.61	24.59	21.28	-29.97	21.28	24.58	-11.49	-11.50	-8.95	-7.84	-11.54	11.59	12.19	-7.84	-11.54	-11.50	-8.95

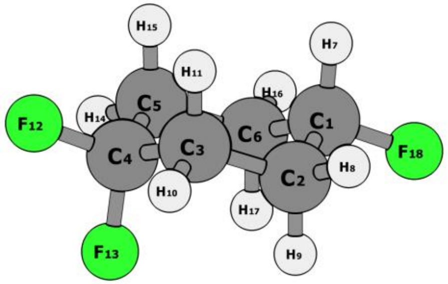

Atom Numbering

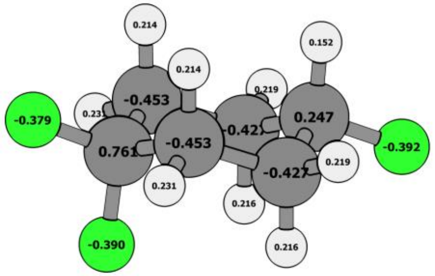

NPA charges

Table S8. Atom-atom electrostatic interactions (kcal mol^{-1}) obtained at M06-2X/aug-cc-pVTZ level using NPA charges for 7$_{eq}$.

	C1	C2	C3	C4	C5	C6	H7	H8	H9	H10	H11	F12	F13	H14	H15	H16	H17
**C2**	-23.09	-	-	-	-	-	-	-	-	-	-	-	-	-	-	-	-
**C3**	-14.87	42.03	-	-	-	-	-	-	-	-	-	-	-	-	-	-	-
**C4**	21.60	-43.22	-75.71	-	-	-	-	-	-	-	-	-	-	-	-	-	-
**C5**	-14.87	21.76	26.85	-75.69	-	-	-	-	-	-	-	-	-	-	-	-	-
**C6**	-23.08	24.09	21.76	-43.21	42.00	-	-	-	-	-	-	-	-	-	-	-	-
**H7**	11.38	-9.99	-8.22	11.65	-8.21	-9.99	-	-	-	-	-	-	-	-	-	-	-
**H8**	8.36	-28.47	-15.19	16.05	-8.35	-8.98	4.47	-	-	-	-	-	-	-	-	-	-
**H9**	8.34	-28.08	-15.05	19.67	-9.76	-11.14	3.58	8.92	-	-	-	-	-	-	-	-	-
**H10**	5.47	-15.01	-31.83	27.46	-10.04	-8.33	3.07	6.58	6.64	-	-	-	-	-	-	-	-
**H11**	6.30	-13.99	-29.49	25.63	-11.46	-9.01	4.12	6.23	5.01	9.30	-	-	-	-	-	-	-
**F12**	-7.53	14.56	24.24	-70.26	24.24	14.55	-4.43	-6.12	-6.75	-11.18	-10.57	-	-	-	-	-	-
**F13**	-9.17	18.91	24.96	-71.85	24.96	18.90	-4.68	-7.24	-10.53	-11.72	-8.49	22.50	-	-	-	-	-
**H14**	5.47	-8.33	-10.04	27.46	-31.83	-15.00	3.07	3.38	4.02	4.17	4.34	-11.17	-11.71	-	-	-	-
**H15**	6.30	-9.02	-11.47	25.63	-29.50	-13.99	4.12	3.72	3.83	4.34	5.77	-10.57	-8.49	9.30	-	-	-
**H16**	8.36	-8.98	-8.35	16.05	-15.18	-28.47	4.47	3.72	4.21	3.38	3.72	-6.12	-7.24	6.58	6.23	-	-
**H17**	8.34	-11.14	-9.76	19.66	-15.05	-28.08	3.58	4.21	6.12	4.02	3.83	-6.75	-10.53	6.64	5.01	8.92	-
**F18**	-23.18	23.48	15.88	-23.80	15.88	23.48	-9.89	-10.87	-10.87	-6.63	-6.87	9.04	11.29	-6.62	-6.87	-10.87	-10.87

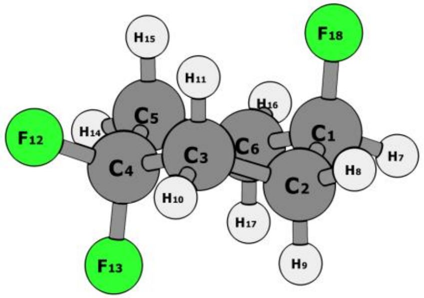

Atom Numbering

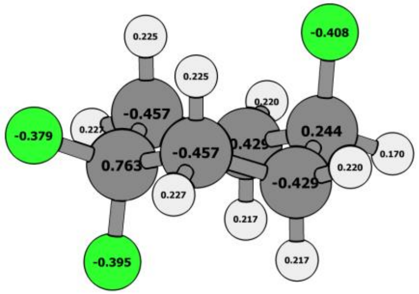

NPA charges

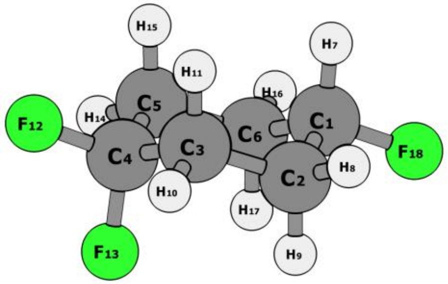

Atom Numbering

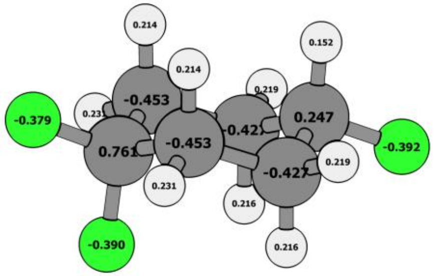

NPA charges

Table S9. $\Delta_{7ax\text{-}7eq}$ Atom-atom electrostatic interactions (kcal mol^{-1}) obtained at M06-2X/aug-cc-pVTZ level using NPA charges.

	C1	C2	C3	C4	C5	C6	H7	H8	H9	H10	H11	F12	F13	H14	H15	H16	H17
**C2**	0.25	-	-	-	-	-	-	-	-	-	-	-	-	-	-	-	-
**C3**	0.17	0.56	-	-	-	-	-	-	-	-	-	-	-	-	-	-	-
**C4**	-0.33	-0.29	-0.97	-	-	-	-	-	-	-	-	-	-	-	-	-	-
**C5**	0.16	0.27	0.59	-0.99	-	-	-	-	-	-	-	-	-	-	-	-	-
**C6**	0.25	0.07	0.27	-0.29	0.58	-	-	-	-	-	-	-	-	-	-	-	-
**H7**	1.21	-1.20	0.79	-0.63	0.78	-1.20	-	-	-	-	-	-	-	-	-	-	-
**H8**	-0.07	-0.22	-0.23	0.11	-0.13	-0.06	0.52	-	-	-	-	-	-	-	-	-	-
**H9**	-0.08	-0.26	-0.23	0.06	-0.05	0.09	1.41	0.08	-	-	-	-	-	-	-	-	-
**H10**	-0.18	0.20	0.24	-0.42	0.06	0.12	-0.10	-0.09	-0.04	-	-	-	-	-	-	-	-
**H11**	0.19	-0.83	-1.89	1.37	-0.79	-0.54	-0.78	0.40	0.30	0.30	-	-	-	-	-	-	-
**F12**	0.15	0.05	0.23	-0.07	0.23	0.05	0.32	-0.02	-0.03	0.17	-0.47	-	-	-	-	-	-
**F13**	-0.06	0.43	0.58	-0.89	0.58	0.44	-0.57	-0.14	-0.23	0.09	-0.55	0.28	-	-	-	-	-
**H14**	-0.18	0.12	0.06	-0.42	0.24	0.19	-0.10	-0.04	-0.09	-0.14	0.16	0.16	0.08	-	-	-	-
**H15**	0.19	-0.53	-0.78	1.37	-1.88	-0.83	-0.78	0.23	0.20	0.16	0.69	-0.47	-0.55	0.30	-	-	-
**H16**	-0.07	-0.06	-0.13	0.11	-0.24	-0.22	0.52	0.04	-0.02	-0.04	0.23	-0.02	-0.14	-0.09	0.40	-	-
**H17**	-0.08	0.09	-0.05	0.07	-0.23	-0.26	1.41	-0.02	-0.16	-0.09	0.20	-0.03	-0.23	-0.04	0.30	0.08	-
**F18**	-0.43	1.11	5.40	-6.17	5.40	1.10	-1.60	-0.63	1.92	-1.21	-4.67	2.55	0.90	-1.22	-4.67	-0.63	1.92

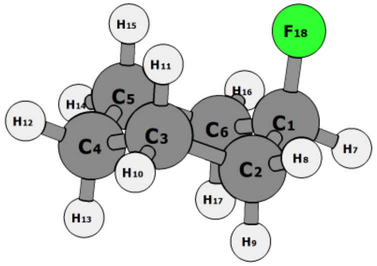

Atom Numbering

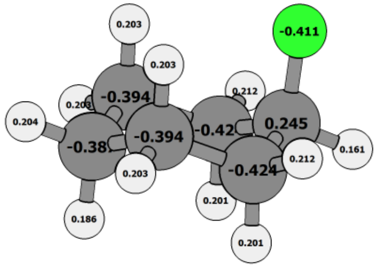

NPA charges

Table S10. Atom-atom electrostatic interactions (kcal mol^{-1}) obtained at M06-2X/aug-cc-pVTZ level using NPA charges for 8_{ax}.

	C1	C2	C3	C4	C5	C6	H7	H8	H9	H10	H11	F12	F13	H14	H15	H16	H17
**C2**	-22.68	-	-	-	-	-	-	-	-	-	-	-	-	-	-	-	-
**C3**	-12.70	36.28	-	-	-	-	-	-	-	-	-	-	-	-	-	-	-
**C4**	-10.71	21.66	33.16	-	-	-	-	-	-	-	-	-	-	-	-	-	-
**C5**	-12.70	18.80	20.48	33.16	-	-	-	-	-	-	-	-	-	-	-	-	-
**C6**	-22.68	23.62	18.80	21.66	36.26	-	-	-	-	-	-	-	-	-	-	-	-
**H7**	12.00	-10.50	-6.08	-5.28	-6.08	-10.51	-	-	-	-	-	-	-	-	-	-	-
**H8**	8.07	-27.41	-12.77	-7.87	-7.07	-8.65	4.59	-	-	-	-	-	-	-	-	-	-
**H9**	7.67	-25.82	-12.14	-9.23	-7.80	-10.07	4.39	8.04	-	-	-	-	-	-	-	-	-
**H10**	4.76	-13.23	-24.31	-12.04	-7.65	-7.24	2.53	5.69	5.45	-	-	-	-	-	-	-	-
**H11**	5.90	-13.30	-24.27	-12.14	-9.57	-8.56	2.87	5.79	4.43	7.78	-	-	-	-	-	-	-
**F12**	4.21	-8.29	-12.34	-24.09	-12.34	-8.29	2.20	3.34	3.59	5.50	5.54	-	-	-	-	-	-
**F13**	4.55	-9.41	-11.29	-21.84	-11.29	-9.41	2.41	3.46	4.74	5.04	4.10	7.18	-	-	-	-	-
**H14**	4.76	-7.24	-7.65	-12.04	-24.31	-13.23	2.53	2.88	3.23	3.17	3.63	5.50	5.04	-	-	-	-
**H15**	5.90	-8.56	-9.57	-12.14	-24.27	-13.29	2.87	3.46	3.37	3.63	5.30	5.54	4.10	7.78	-	-	-
**H16**	8.07	-8.65	-7.07	-7.87	-12.77	-27.41	4.59	3.52	3.74	2.88	3.46	3.34	3.46	5.69	5.79	-	-
**H17**	7.67	-10.07	-7.80	-9.23	-12.14	-25.82	4.39	3.74	5.07	3.23	3.37	3.59	4.74	5.45	4.43	8.04	-
**F18**	-23.84	24.48	18.34	15.05	18.33	24.47	-11.01	-11.23	-8.32	-7.08	-10.46	-6.49	-6.01	-7.07	-10.46	-11.23	-8.32

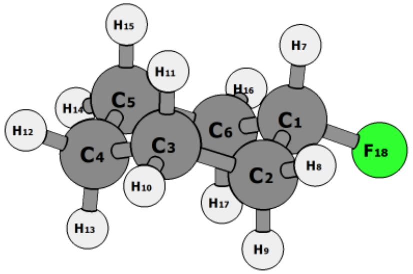

Atom Numbering

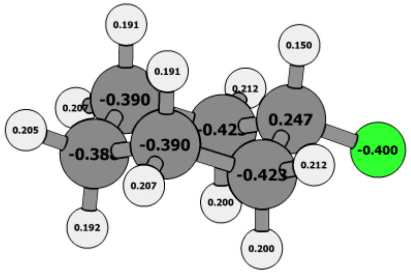

NPA charges

Table S11. Atom-atom electrostatic interactions (kcal mol^{-1}) obtained at M06-2X/aug-cc-pVTZ level using NPA charges for 8_{eq}.

	C1	C2	C3	C4	C5	C6	H7	H8	H9	H10	H11	F12	F13	H14	H15	H16	H17
**C2**	-22.89	-	-	-	-	-	-	-	-	-	-	-	-	-	-	-	-
**C3**	-12.80	35.82	-	-	-	-	-	-	-	-	-	-	-	-	-	-	-
**C4**	-10.89	21.64	32.93	-	-	-	-	-	-	-	-	-	-	-	-	-	-
**C5**	-12.80	18.61	20.06	32.93	-	-	-	-	-	-	-	-	-	-	-	-	-
**C6**	-22.88	23.63	18.60	21.64	35.81	-	-	-	-	-	-	-	-	-	-	-	-
**H7**	11.23	-9.79	-6.98	-5.79	-6.98	-9.79	-	-	-	-	-	-	-	-	-	-	-
**H8**	8.10	-27.25	-12.59	-7.86	-6.97	-8.61	4.28	-	-	-	-	-	-	-	-	-	-
**H9**	7.73	-25.71	-11.99	-9.26	-7.79	-10.21	3.28	7.99	-	-	-	-	-	-	-	-	-
**H10**	4.93	-13.47	-24.61	-12.33	-7.74	-7.39	2.73	5.80	5.53	-	-	-	-	-	-	-	-
**H11**	5.67	-12.47	-22.63	-11.47	-8.89	-8.03	3.66	5.40	4.16	7.51	-	-	-	-	-	-	-
**F12**	4.29	-8.28	-12.25	-24.20	-12.25	-8.28	2.45	3.34	3.59	5.62	5.26	-	-	-	-	-	-
**F13**	4.72	-9.65	-11.54	-22.59	-11.54	-9.65	2.38	3.56	4.88	5.32	3.99	7.42	-	-	-	-	-
**H14**	4.93	-7.39	-7.74	-12.33	-24.61	-13.47	2.74	2.93	3.32	3.31	3.48	5.62	5.32	-	-	-	-
**H15**	5.67	-8.03	-8.89	-11.47	-22.63	-12.47	3.66	3.23	3.18	3.48	4.66	5.26	3.99	7.51	-	-	-
**H16**	8.10	-8.61	-6.97	-7.85	-12.59	-27.25	4.29	3.49	3.77	2.93	3.23	3.33	3.55	5.80	5.40	-	-
**H17**	7.73	-10.20	-7.79	-9.26	-11.99	-25.70	3.28	3.77	5.23	3.32	3.18	3.59	4.88	5.53	4.16	7.99	-
**F18**	-23.54	23.70	13.94	12.30	13.94	23.69	-9.96	-10.74	-10.29	-6.09	-6.28	-5.19	-5.81	-6.09	-6.28	-10.73	-10.28

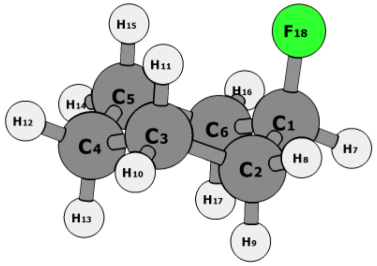

Atom Numbering

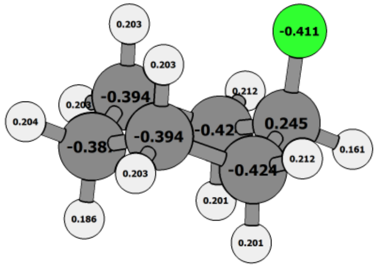

NPA charges

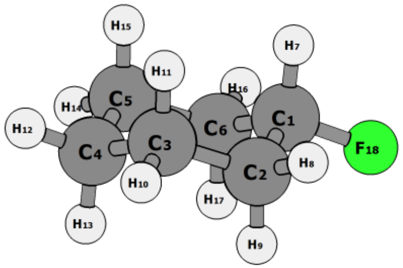

Atom Numbering

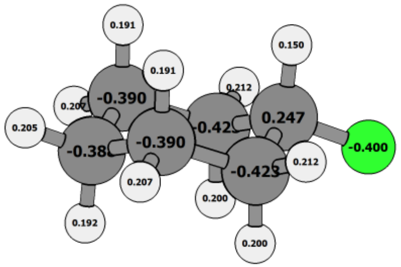

NPA charges

Table S12. $\Delta_{8ax\text{-}8eq}$ **Atom-atom electrostatic interactions (kcal mol^{-1}) obtained at M06-2X/aug-cc-pVTZ level using NPA charges.**

	C1	C2	C3	C4	C5	C6	H7	H8	H9	H10	H11	F12	F13	H14	H15	H16	H17
**C2**	0.21	-	-	-	-	-	-	-	-	-	-	-	-	-	-	-	-
**C3**	0.10	0.46	-	-	-	-	-	-	-	-	-	-	-	-	-	-	-
**C4**	0.18	0.02	0.23	-	-	-	-	-	-	-	-	-	-	-	-	-	-
**C5**	0.10	0.19	0.42	0.23	-	-	-	-	-	-	-	-	-	-	-	-	-
**C6**	0.20	-0.01	0.20	0.02	0.45	-	-	-	-	-	-	-	-	-	-	-	-
**H7**	0.77	-0.71	0.90	0.51	0.90	-0.72	-	-	-	-	-	-	-	-	-	-	-
**H8**	-0.03	-0.16	-0.18	-0.01	-0.10	-0.04	0.31	-	-	-	-	-	-	-	-	-	-
**H9**	-0.06	-0.11	-0.15	0.03	-0.01	0.14	1.11	0.05	-	-	-	-	-	-	-	-	-
**H10**	-0.17	0.24	0.30	0.29	0.09	0.15	-0.20	-0.11	-0.08	-	-	-	-	-	-	-	-
**H11**	0.23	-0.83	-1.64	-0.67	-0.68	-0.53	-0.79	0.39	0.27	0.27	-	-	-	-	-	-	-
**F12**	-0.08	-0.01	-0.09	0.11	-0.09	-0.01	-0.25	0.00	0.00	-0.12	0.28	-	-	-	-	-	-
**F13**	-0.17	0.24	0.25	0.75	0.25	0.24	0.03	-0.10	-0.14	-0.28	0.11	-0.24	-	-	-	-	-
**H14**	-0.17	0.15	0.09	0.29	0.30	0.24	-0.21	-0.05	-0.09	-0.14	0.15	-0.12	-0.28	-	-	-	-
**H15**	0.23	-0.53	-0.68	-0.67	-1.64	-0.82	-0.79	0.23	0.19	0.15	0.64	0.28	0.11	0.27	-	-	-
**H16**	-0.03	-0.04	-0.10	-0.02	-0.18	-0.16	0.30	0.03	-0.03	-0.05	0.23	0.01	-0.09	-0.11	0.39	-	-
**H17**	-0.06	0.13	-0.01	0.03	-0.15	-0.12	1.11	-0.03	-0.16	-0.09	0.19	0.00	-0.14	-0.08	0.27	0.05	-
**F18**	-0.30	0.78	4.40	2.75	4.39	0.78	-1.05	-0.49	1.97	-0.99	-4.18	-1.30	-0.20	-0.98	-4.18	-0.50	1.96

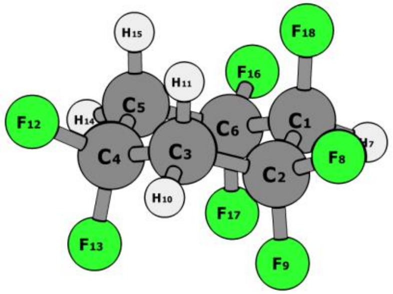

Atom Numbering

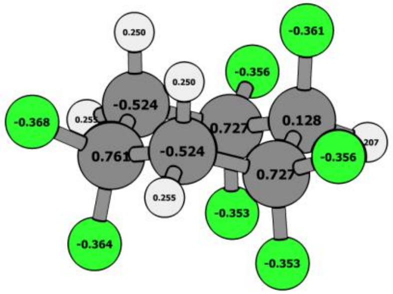

NPA charges

Table S13. Atom-atom electrostatic interactions (kcal mol^{-1}) obtained at M06-2X/aug-cc-pVTZ level using NPA charges for 9_{ax}.

	C1	C2	C3	C4	C5	C6	H7	F8	F9	H10	H11	F12	F13	H14	H15	F16	F17
**C2**	20.21	-	-	-	-	-	-	-	-	-	-	-	-	-	-	-	-
**C3**	-8.77	-83.54	-	-	-	-	-	-	-	-	-	-	-	-	-	-	-
**C4**	10.98	73.13	-87.22	-	-	-	-	-	-	-	-	-	-	-	-	-	-
**C5**	-8.77	-42.82	35.75	-87.22	-	-	-	-	-	-	-	-	-	-	-	-	-
**C6**	20.21	69.57	-42.82	73.13	-83.54	-	-	-	-	-	-	-	-	-	-	-	-
**H7**	8.06	23.08	-10.36	13.31	-10.36	23.09	-	-	-	-	-	-	-	-	-	-	-
**F8**	-6.47	-63.43	26.44	-24.41	14.85	-23.29	-9.28	-	-	-	-	-	-	-	-	-	-
**F9**	-6.42	-63.06	26.01	-29.84	17.34	-28.86	-9.50	19.19	-	-	-	-	-	-	-	-	-
**H10**	3.12	28.75	-40.67	30.01	-12.75	15.70	4.09	-11.34	-11.64	-	-	-	-	-	-	-	-
**H11**	3.76	28.44	-39.90	29.65	-15.25	17.89	4.51	-11.69	-8.97	11.88	-	-	-	-	-	-	-
**F12**	-3.76	-24.12	27.36	-68.24	27.36	-24.12	-4.87	9.32	10.16	-11.71	-12.10	-	-	-	-	-	-
**F13**	-4.31	-29.25	26.90	-67.70	26.90	-29.25	-5.73	10.14	14.99	-12.15	-9.24	20.41	-	-	-	-	-
**H14**	3.12	15.70	-12.75	30.01	-40.67	28.75	4.09	-5.80	-6.99	5.05	5.50	-11.71	-12.15	-	-	-	-
**H15**	3.76	17.89	-15.25	29.65	-39.91	28.45	4.51	-6.76	-6.88	5.50	7.61	-12.10	-9.24	11.88	-	-	-
**F16**	-6.47	-23.30	14.85	-24.41	26.44	-63.44	-9.28	9.01	9.98	-5.80	-6.76	9.32	10.14	-11.34	-11.69	-	-
**F17**	-6.42	-28.85	17.34	-29.83	26.01	-63.06	-9.50	9.98	15.14	-6.99	-6.88	10.16	14.99	-11.64	-8.96	19.19	-
**F18**	-11.19	-37.31	21.59	-26.53	21.59	-37.31	-12.28	16.04	12.10	-7.81	-11.32	10.16	9.83	-7.81	-11.32	16.04	12.10

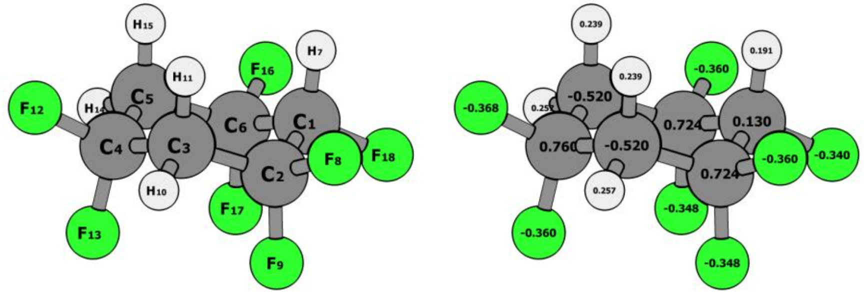

Atom Numbering **NPA charges**

Table S14. Atom-atom electrostatic interactions (kcal mol^{-1}) obtained at M06-2X/aug-cc-pVTZ level using NPA charges for 9_{eq}.

	C1	C2	C3	C4	C5	C6	H7	F8	F9	H10	H11	F12	F13	H14	H15	F16	F17
**C2**	20.45	-	-	-	-	-	-	-	-	-	-	-	-	-	-	-	-
**C3**	-8.84	-82.49	-	-	-	-	-	-	-	-	-	-	-	-	-	-	-
**C4**	11.13	72.66	-86.50	-	-	-	-	-	-	-	-	-	-	-	-	-	-
**C5**	-8.84	-42.34	35.30	-86.51	-	-	-	-	-	-	-	-	-	-	-	-	-
**C6**	20.45	68.95	-42.34	72.65	-82.50	-	-	-	-	-	-	-	-	-	-	-	-
**H7**	7.53	21.50	-11.74	14.47	-11.74	21.50	-	-	-	-	-	-	-	-	-	-	-
**F8**	-6.63	-63.75	26.55	-24.65	14.92	-23.45	-9.01	-	-	-	-	-	-	-	-	-	-
**F9**	-6.38	-62.04	25.49	-29.28	16.87	-28.05	-6.76	19.14	-	-	-	-	-	-	-	-	-
**H10**	3.20	29.00	-40.79	30.29	-12.81	15.81	4.29	-11.61	-11.68	-	-	-	-	-	-	-	-
**H11**	3.65	27.03	-37.92	28.38	-14.51	17.04	5.70	-11.33	-8.47	11.53	-	-	-	-	-	-	-
**F12**	-3.82	-24.01	27.16	-68.20	27.16	-24.01	-5.42	9.43	10.01	-11.83	-11.62	-	-	-	-	-	-
**F13**	-4.33	-28.81	26.42	-67.03	26.43	-28.81	-5.35	10.13	14.56	-12.14	-8.77	20.21	-	-	-	-	-
**H14**	3.20	15.81	-12.81	30.30	-40.79	29.00	4.29	-5.93	-6.93	5.16	5.33	-11.83	-12.14	-	-	-	-
**H15**	3.65	17.04	-14.51	28.38	-37.93	27.04	5.70	-6.55	-6.46	5.33	6.99	-11.63	-8.77	11.53	-	-	-
**F16**	-6.63	-23.45	14.92	-24.65	26.55	-63.75	-9.01	9.22	9.86	-5.93	-6.55	9.43	10.13	-11.61	-11.34	-	-
**F17**	-6.38	-28.04	16.87	-29.27	25.49	-62.04	-6.76	9.86	14.34	-6.92	-6.46	10.00	14.56	-11.69	-8.47	19.14	-
**F18**	-10.78	-34.60	15.81	-20.56	15.82	-34.61	-10.77	14.63	14.69	-6.48	-6.64	7.62	8.92	-6.48	-6.64	14.63	14.69

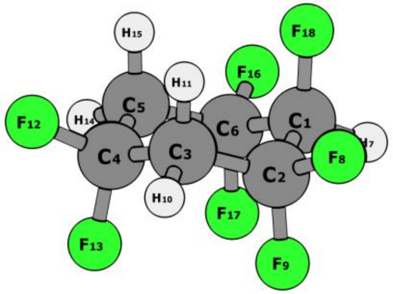

Atom Numbering

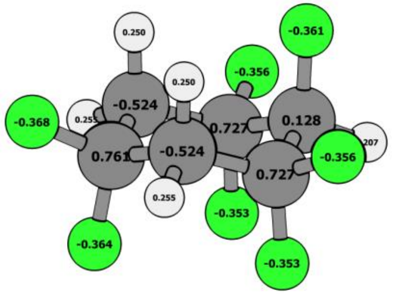

NPA charges

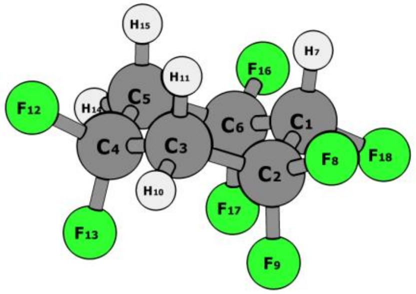

Atom Numbering

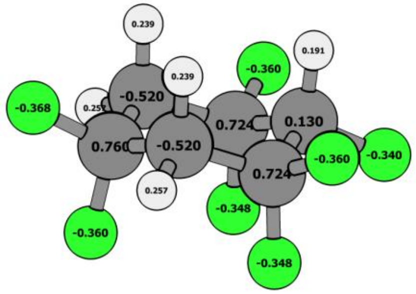

NPA charges

Table S15. $\Delta_{9ax\text{-}9eq}$ **Atom-atom electrostatic interactions (kcal mol^{-1}) obtained at M06-2X/aug-cc-pVTZ level using NPA charges.**

	C1	C2	C3	C4	C5	C6	H7	F8	F9	H10	H11	F12	F13	H14	H15	F16	F17
**C2**	-0.24	-	-	-	-	-	-	-	-	-	-	-	-	-	-	-	-
**C3**	0.07	-1.05	-	-	-	-	-	-	-	-	-	-	-	-	-	-	-
**C4**	-0.15	0.47	-0.72	-	-	-	-	-	-	-	-	-	-	-	-	-	-
**C5**	0.07	-0.48	0.45	-0.71	-	-	-	-	-	-	-	-	-	-	-	-	-
**C6**	-0.24	0.62	-0.48	0.48	-1.04	-	-	-	-	-	-	-	-	-	-	-	-
**H7**	0.53	1.58	1.38	-1.16	1.38	1.59	-	-	-	-	-	-	-	-	-	-	-
**F8**	0.16	0.32	-0.11	0.24	-0.07	0.16	-0.27	-	-	-	-	-	-	-	-	-	-
**F9**	-0.04	-1.02	0.52	-0.56	0.47	-0.81	-2.74	0.05	-	-	-	-	-	-	-	-	-
**H10**	-0.08	-0.25	0.12	-0.28	0.06	-0.11	-0.20	0.27	0.04	-	-	-	-	-	-	-	-
**H11**	0.11	1.41	-1.98	1.27	-0.74	0.85	-1.19	-0.36	-0.50	0.35	-	-	-	-	-	-	-
**F12**	0.06	-0.11	0.20	-0.04	0.20	-0.11	0.55	-0.11	0.15	0.12	-0.48	-	-	-	-	-	-
**F13**	0.02	-0.44	0.48	-0.67	0.47	-0.44	-0.38	0.01	0.43	-0.01	-0.47	0.20	-	-	-	-	-
**H14**	-0.08	-0.11	0.06	-0.29	0.12	-0.25	-0.20	0.13	-0.06	-0.11	0.17	0.12	-0.01	-	-	-	-
**H15**	0.11	0.85	-0.74	1.27	-1.98	1.41	-1.19	-0.21	-0.42	0.17	0.62	-0.47	-0.47	0.35	-	-	-
**F16**	0.16	0.15	-0.07	0.24	-0.11	0.31	-0.27	-0.21	0.12	0.13	-0.21	-0.11	0.01	0.27	-0.35	-	-
**F17**	-0.04	-0.81	0.47	-0.56	0.52	-1.02	-2.74	0.12	0.80	-0.07	-0.42	0.16	0.43	0.05	-0.49	0.05	-
**F18**	-0.41	-2.71	5.78	-5.97	5.77	-2.70	-1.51	1.41	-2.59	-1.33	-4.68	2.54	0.91	-1.33	-4.68	1.41	-2.59

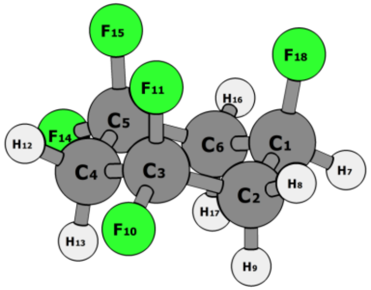

Atom Numbering

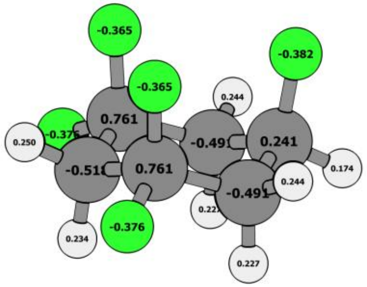

NPA charges

Table S16. Atom-atom electrostatic interactions (kcal mol^{-1}) obtained at M06-2X/aug-cc-pVTZ level using NPA charges for 10_{ax}.

	C1	C2	C3	C4	C5	C6	H7	H8	H9	F10	F11	H12	H13	F14	F15	H16	H17
**C2**	-25.85	-	-	-	-	-	-	-	-	-	-	-	-	-	-	-	-
**C3**	24.10	-82.01	-	-	-	-	-	-	-	-	-	-	-	-	-	-	-
**C4**	-13.97	33.25	-86.44	-	-	-	-	-	-	-	-	-	-	-	-	-	-
**C5**	24.10	-42.22	76.67	-86.44	-	-	-	-	-	-	-	-	-	-	-	-	-
**C6**	-25.85	31.63	-42.22	33.25	-82.00	-	-	-	-	-	-	-	-	-	-	-	-
**H7**	12.78	-13.24	12.74	-7.61	12.74	-13.24	-	-	-	-	-	-	-	-	-	-	-
**H8**	9.07	-36.58	29.00	-12.15	15.80	-11.49	5.59	-	-	-	-	-	-	-	-	-	-
**H9**	8.50	-33.98	27.13	-13.76	17.07	-13.02	5.45	10.42	-	-	-	-	-	-	-	-	-
**F10**	-8.14	26.21	-69.65	27.70	-25.85	14.77	-4.87	-11.57	-11.34	-	-	-	-	-	-	-	-
**F11**	-9.66	25.31	-67.98	26.71	-30.68	16.64	-5.26	-11.73	-8.46	20.98	-	-	-	-	-	-	-
**H12**	5.09	-11.76	29.50	-39.48	29.50	-11.76	2.92	4.77	4.92	-11.77	-11.98	-	-	-	-	-	-
**H13**	5.54	-13.44	27.88	-36.99	27.88	-13.43	3.25	4.96	6.52	-11.65	-8.71	10.95	-	-	-	-	-
**F14**	-8.14	14.77	-25.84	27.70	-69.65	26.21	-4.87	-5.89	-6.52	10.10	10.73	-11.77	-11.65	-	-	-	-
**F15**	-9.66	16.65	-30.69	26.71	-67.99	25.31	-5.26	-6.86	-6.42	10.73	15.42	-11.98	-8.71	20.98	-	-	-
**H16**	9.07	-11.49	15.80	-12.15	29.00	-36.58	5.59	4.63	4.79	-5.89	-6.86	4.77	4.96	-11.57	-11.73	-	-
**H17**	8.50	-13.02	17.06	-13.76	27.13	-33.97	5.45	4.79	6.28	-6.52	-6.41	4.92	6.52	-11.34	-8.46	10.42	-
**F18**	-22.08	26.28	-31.97	18.27	-31.97	26.28	-11.07	-12.09	-8.75	11.18	16.14	-7.33	-6.89	11.18	16.14	-12.09	-8.75

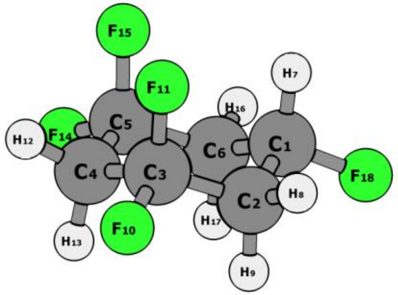

Atom Numbering

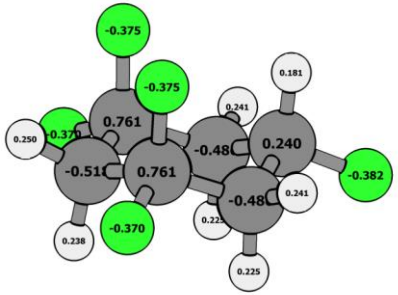

NPA charges

Table S17. Atom-atom electrostatic interactions (kcal mol^{-1}) obtained at M06-2X/aug-cc-pVTZ level using NPA charges for 10_{eq}.

	C1	C2	C3	C4	C5	C6	H7	H8	H9	F10	F11	H12	H13	F14	F15	H16	H17
**C2**	-25.50	-	-	-	-	-	-	-	-	-	-	-	-	-	-	-	-
**C3**	24.38	-81.10	-	-	-	-	-	-	-	-	-	-	-	-	-	-	-
**C4**	-14.04	32.87	-86.31	-	-	-	-	-	-	-	-	-	-	-	-	-	-
**C5**	24.38	-41.88	76.62	-86.32	-	-	-	-	-	-	-	-	-	-	-	-	-
**C6**	-25.50	31.21	-41.88	32.87	-81.10	-	-	-	-	-	-	-	-	-	-	-	-
**H7**	13.23	-13.53	16.42	-9.32	16.42	-13.53	-	-	-	-	-	-	-	-	-	-	-
**H8**	8.88	-35.69	28.59	-11.97	15.55	-11.23	5.85	-	-	-	-	-	-	-	-	-	-
**H9**	8.37	-33.23	26.67	-13.68	17.15	-13.13	4.43	10.14	-	-	-	-	-	-	-	-	-
**F10**	-8.02	25.43	-68.73	27.23	-25.42	14.40	-5.51	-11.31	-10.78	-	-	-	-	-	-	-	-
**F11**	-10.22	25.81	-69.39	27.41	-31.59	17.02	-8.41	-11.82	-8.56	21.16	-	-	-	-	-	-	-
**H12**	5.13	-11.63	29.44	-39.46	29.44	-11.63	3.68	4.70	4.87	-11.57	-12.28	-	-	-	-	-	-
**H13**	5.58	-13.46	28.30	-37.60	28.30	-13.46	3.53	4.98	6.57	-11.61	-9.08	11.12	-	-	-	-	-
**F14**	-8.02	14.40	-25.42	27.23	-68.73	25.43	-5.51	-5.71	-6.42	9.77	10.87	-11.57	-11.61	-	-	-	-
**F15**	-10.22	17.02	-31.59	27.41	-69.39	25.81	-8.41	-6.94	-6.60	10.87	16.43	-12.28	-9.08	21.16	-	-	-
**H16**	8.88	-11.23	15.55	-11.97	28.59	-35.69	5.85	4.48	4.78	-5.71	-6.94	4.70	4.98	-11.31	-11.82	-	-
**H17**	8.37	-13.13	17.15	-13.68	26.67	-33.23	4.43	4.78	6.55	-6.42	-6.60	4.87	6.57	-10.78	-8.56	10.14	-
**F18**	-22.02	26.16	-26.18	15.64	-26.18	26.16	-11.48	-11.51	-11.04	10.01	11.40	-6.09	-6.78	10.01	11.40	-11.51	-11.04

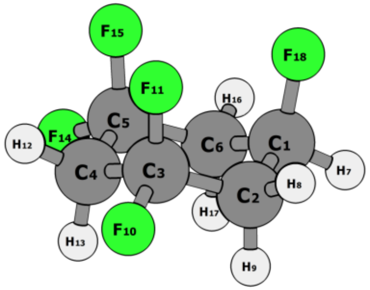

Atom Numbering

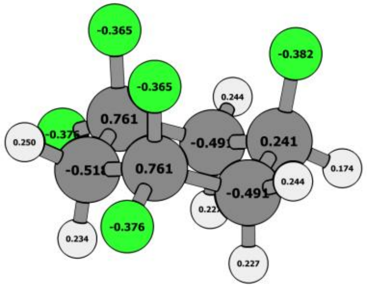

NPA charges

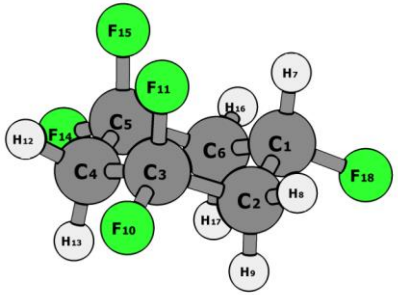

Atom Numbering

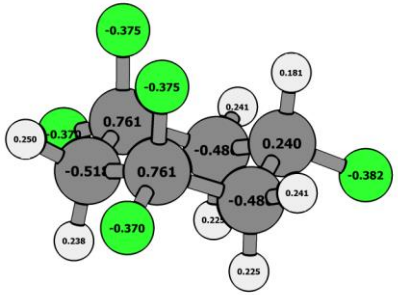

NPA charges

Table S18. $\Delta_{10ax\text{-}10eq}$ **Atom-atom electrostatic interactions (kcal mol^{-1}) obtained at M06-2X/aug-cc-pVTZ level using NPA charges.**

	C1	C2	C3	C4	C5	C6	H7	H8	H9	F10	F11	H12	H13	F14	F15	H16	H17
**C2**	-0.35	-	-	-	-	-	-	-	-	-	-	-	-	-	-	-	-
**C3**	-0.28	-0.91	-	-	-	-	-	-	-	-	-	-	-	-	-	-	-
**C4**	0.07	0.38	-0.13	-	-	-	-	-	-	-	-	-	-	-	-	-	-
**C5**	-0.28	-0.34	0.05	-0.12	-	-	-	-	-	-	-	-	-	-	-	-	-
**C6**	-0.35	0.42	-0.34	0.38	-0.90	-	-	-	-	-	-	-	-	-	-	-	-
**H7**	-0.45	0.29	-3.68	1.71	-3.68	0.29	-	-	-	-	-	-	-	-	-	-	-
**H8**	0.19	-0.89	0.41	-0.18	0.25	-0.26	-0.26	-	-	-	-	-	-	-	-	-	-
**H9**	0.13	-0.75	0.46	-0.08	-0.08	0.11	1.02	0.28	-	-	-	-	-	-	-	-	-
**F10**	-0.12	0.78	-0.92	0.47	-0.43	0.37	0.64	-0.26	-0.56	-	-	-	-	-	-	-	-
**F11**	0.56	-0.50	1.41	-0.70	0.91	-0.38	3.15	0.09	0.10	-0.18	-	-	-	-	-	-	-
**H12**	-0.04	-0.13	0.06	-0.02	0.06	-0.13	-0.76	0.07	0.05	-0.20	0.30	-	-	-	-	-	-
**H13**	-0.04	0.02	-0.42	0.61	-0.42	0.03	-0.28	-0.02	-0.05	-0.04	0.37	-0.17	-	-	-	-	-
**F14**	-0.12	0.37	-0.42	0.47	-0.92	0.78	0.64	-0.18	-0.10	0.33	-0.14	-0.20	-0.04	-	-	-	-
**F15**	0.56	-0.37	0.90	-0.70	1.40	-0.50	3.15	0.08	0.18	-0.14	-1.01	0.30	0.37	-0.18	-	-	-
**H16**	0.19	-0.26	0.25	-0.18	0.41	-0.89	-0.26	0.15	0.01	-0.18	0.08	0.07	-0.02	-0.26	0.09	-	-
**H17**	0.13	0.11	-0.09	-0.08	0.46	-0.74	1.02	0.01	-0.27	-0.10	0.19	0.05	-0.05	-0.56	0.10	0.28	-
**F18**	-0.06	0.12	-5.79	2.63	-5.79	0.12	0.41	-0.58	2.29	1.17	4.74	-1.24	-0.11	1.17	4.74	-0.58	2.29

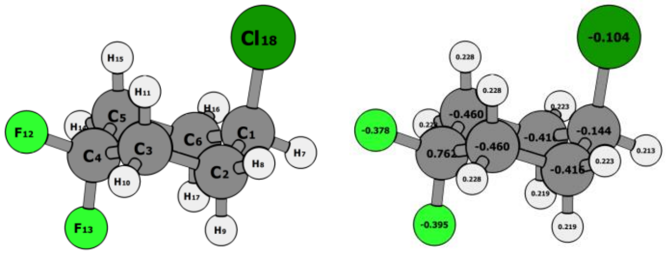

Atom Numbering NPA charges

Table S19. Atom-atom electrostatic interactions (kcal mol^{-1}) obtained at M06-2X/aug-cc-pVTZ level using NPA charges for 11$_{ax}$.

	C1	C2	C3	C4	C5	C6	H7	H8	H9	H10	H11	F12	F13	H14	H15	H16	H17
**C2**	13.10	-	-	-	-	-	-	-	-	-	-	-	-	-	-	-	-
**C3**	8.71	41.56	-	-	-	-	-	-	-	-	-	-	-	-	-	-	-
**C4**	-12.54	-42.19	-77.02	-	-	-	-	-	-	-	-	-	-	-	-	-	-
**C5**	8.71	21.49	27.70	-77.02	-	-	-	-	-	-	-	-	-	-	-	-	-
**C6**	13.10	22.75	21.49	-42.19	41.56	-	-	-	-	-	-	-	-	-	-	-	-
**H7**	-9.42	-13.66	-9.38	13.84	-9.38	-13.66	-	-	-	-	-	-	-	-	-	-	-
**H8**	-4.97	-28.29	-15.75	16.40	-8.64	-8.89	6.34	-	-	-	-	-	-	-	-	-	-
**H9**	-4.96	-27.68	-15.54	20.00	-10.00	-10.88	6.39	9.22	-	-	-	-	-	-	-	-	-
**H10**	-3.15	-14.50	-31.97	27.22	-10.10	-8.03	3.76	6.66	6.73	-	-	-	-	-	-	-	-
**H11**	-3.86	-14.55	-31.94	27.30	-12.42	-9.36	4.22	6.79	5.43	9.78	-	-	-	-	-	-	-
**F12**	4.35	14.14	24.53	-70.15	24.53	14.14	-5.15	-6.22	-6.84	-11.04	-11.16	-	-	-	-	-	-
**F13**	5.46	18.76	25.68	-72.68	25.68	18.75	-6.62	-7.50	-10.91	-11.74	-9.16	22.73	-	-	-	-	-
**H14**	-3.15	-8.03	-10.10	27.22	-31.97	-14.50	3.76	3.41	4.02	4.09	4.58	-11.04	-11.74	-	-	-	-
**H15**	-3.86	-9.36	-12.42	27.30	-31.94	-14.55	4.22	4.04	4.13	4.58	6.56	-11.16	-9.16	9.78	-	-	-
**H16**	-4.97	-8.89	-8.64	16.40	-15.75	-28.29	6.34	3.86	4.31	3.41	4.04	-6.22	-7.50	6.66	6.79	-	-
**H17**	-4.96	-10.88	-10.00	20.00	-15.54	-27.67	6.39	4.31	6.15	4.02	4.13	-6.84	-10.91	6.73	5.43	9.22	-
**Cl18**	2.76	5.27	4.90	-6.93	4.90	5.26	-3.17	-2.69	-2.06	-1.87	-2.75	2.77	2.85	-1.87	-2.75	-2.69	-2.06

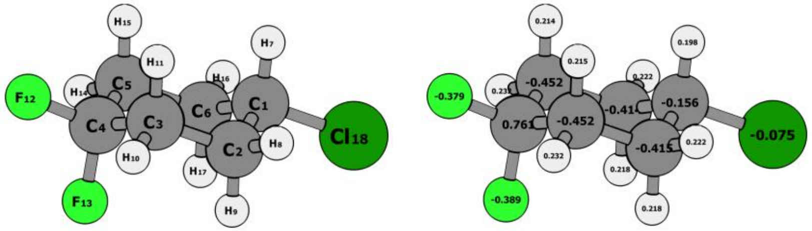

Atom Numbering **NPA charges**

Table S20. Atom-atom electrostatic interactions (kcal mol^{-1}) obtained at M06-2X/aug-cc-pVTZ level using NPA charges for 11_{eq}.

	C1	C2	C3	C4	C5	C6	H7	H8	H9	H10	H11	F12	F13	H14	H15	H16	H17
**C2**	14.10	-	-	-	-	-	-	-	-	-	-	-	-	-	-	-	-
**C3**	9.35	40.71	-	-	-	-	-	-	-	-	-	-	-	-	-	-	-
**C4**	-13.60	-41.92	-75.59	-	-	-	-	-	-	-	-	-	-	-	-	-	-
**C5**	9.35	21.09	26.78	-75.57	-	-	-	-	-	-	-	-	-	-	-	-	-
**C6**	14.10	22.69	21.08	-41.91	40.69	-	-	-	-	-	-	-	-	-	-	-	-
**H7**	-9.42	-12.68	-10.73	15.23	-10.73	-12.68	-	-	-	-	-	-	-	-	-	-	-
**H8**	-5.33	-28.05	-15.39	16.27	-8.45	-8.83	5.91	-	-	-	-	-	-	-	-	-	-
**H9**	-5.28	-27.52	-15.17	19.82	-9.82	-10.89	4.72	9.14	-	-	-	-	-	-	-	-	-
**H10**	-3.46	-14.65	-31.90	27.57	-10.07	-8.12	4.03	6.72	6.75	-	-	-	-	-	-	-	-
**H11**	-3.98	-13.60	-29.49	25.65	-11.46	-8.76	5.39	6.33	5.07	9.34	-	-	-	-	-	-	-
**F12**	4.74	14.11	24.17	-70.14	24.16	14.10	-5.78	-6.20	-6.80	-11.20	-10.56	-	-	-	-	-	-
**F13**	5.77	18.34	24.90	-71.74	24.89	18.34	-6.11	-7.33	-10.62	-11.76	-8.49	22.44	-	-	-	-	-
**H14**	-3.46	-8.13	-10.07	27.56	-31.89	-14.64	4.03	3.44	4.07	4.20	4.36	-11.20	-11.75	-	-	-	-
**H15**	-3.98	-8.76	-11.47	25.65	-29.49	-13.60	5.39	3.77	3.87	4.36	5.78	-10.57	-8.49	9.34	-	-	-
**H16**	-5.33	-8.83	-8.45	16.26	-15.38	-28.04	5.91	3.81	4.29	3.44	3.77	-6.20	-7.33	6.72	6.33	-	-
**H17**	-5.28	-10.89	-9.82	19.82	-15.16	-27.52	4.72	4.29	6.19	4.07	3.87	-6.80	-10.61	6.75	5.06	9.14	-
**Cl18**	2.16	3.79	2.74	-4.14	2.74	3.79	-2.12	-1.91	-1.88	-1.18	-1.20	1.60	1.99	-1.18	-1.20	-1.91	-1.88

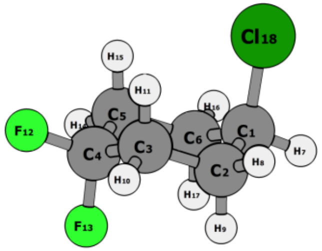

Atom Numbering

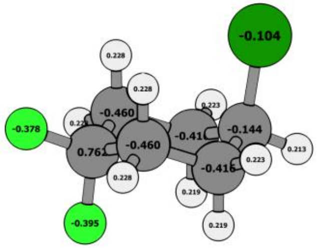

NPA charges

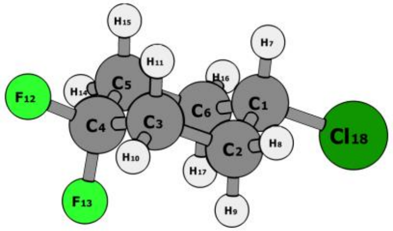

Atom Numbering

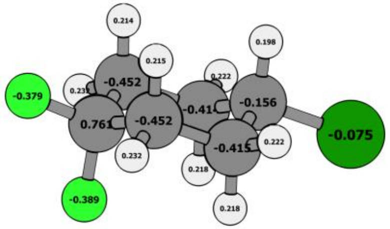

NPA charges

Table S21. $\Delta_{11ax-11eq}$ **Atom-atom electrostatic interactions (kcal mol^{-1}) obtained at M06-2X/aug-cc-pVTZ level using NPA charges.**

	C1	C2	C3	C4	C5	C6	H7	H8	H9	H10	H11	F12	F13	H14	H15	H16	H17
**C2**	-1.00	-	-	-	-	-	-	-	-	-	-	-	-	-	-	-	-
**C3**	-0.64	0.85	-	-	-	-	-	-	-	-	-	-	-	-	-	-	-
**C4**	1.06	-0.27	-1.43	-	-	-	-	-	-	-	-	-	-	-	-	-	-
**C5**	-0.64	0.40	0.92	-1.45	-	-	-	-	-	-	-	-	-	-	-	-	-
**C6**	-1.00	0.06	0.41	-0.28	0.87	-	-	-	-	-	-	-	-	-	-	-	-
**H7**	0.00	-0.98	1.35	-1.39	1.35	-0.98	-	-	-	-	-	-	-	-	-	-	-
**H8**	0.36	-0.24	-0.36	0.13	-0.19	-0.06	0.43	-	-	-	-	-	-	-	-	-	-
**H9**	0.32	-0.16	-0.37	0.18	-0.18	0.01	1.67	0.08	-	-	-	-	-	-	-	-	-
**H10**	0.31	0.15	-0.07	-0.35	-0.03	0.09	-0.27	-0.06	-0.02	-	-	-	-	-	-	-	-
**H11**	0.12	-0.95	-2.45	1.65	-0.96	-0.60	-1.17	0.46	0.36	0.44	-	-	-	-	-	-	-
**F12**	-0.39	0.03	0.36	-0.01	0.37	0.04	0.63	-0.02	-0.04	0.16	-0.60	-	-	-	-	-	-
**F13**	-0.31	0.42	0.78	-0.94	0.79	0.41	-0.51	-0.17	-0.29	0.02	-0.67	0.29	-	-	-	-	-
**H14**	0.31	0.10	-0.03	-0.34	-0.08	0.14	-0.27	-0.03	-0.05	-0.11	0.22	0.16	0.01	-	-	-	-
**H15**	0.12	-0.60	-0.95	1.65	-2.45	-0.95	-1.17	0.27	0.26	0.22	0.78	-0.59	-0.67	0.44	-	-	-
**H16**	0.36	-0.06	-0.19	0.14	-0.37	-0.25	0.43	0.05	0.02	-0.03	0.27	-0.02	-0.17	-0.06	0.46	-	-
**H17**	0.32	0.01	-0.18	0.18	-0.38	-0.15	1.67	0.02	-0.04	-0.05	0.26	-0.04	-0.30	-0.02	0.37	0.08	-
**Cl18**	0.60	1.48	2.16	-2.79	2.16	1.47	-1.05	-0.78	-0.18	-0.69	-1.55	1.17	0.86	-0.69	-1.55	-0.78	-0.18

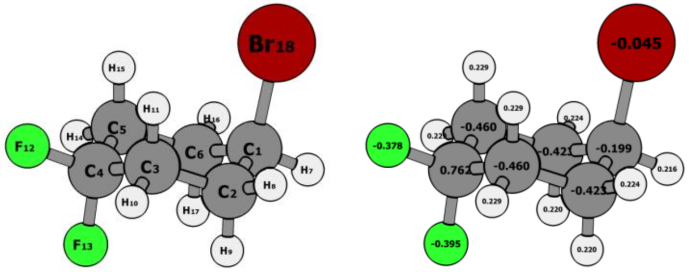

Atom Numbering **NPA charges**

Table S22. Atom-atom electrostatic interactions (kcal mol^{-1}) obtained at M06-2X/aug-cc-pVTZ level using NPA charges for 12$_{ax}$.

	C1	C2	C3	C4	C5	C6	H7	H8	H9	H10	H11	F12	F13	H14	H15	H16	H17
**C2**	18.33	-	-	-	-	-	-	-	-	-	-	-	-	-	-	-	-
**C3**	11.96	42.32	-	-	-	-	-	-	-	-	-	-	-	-	-	-	-
**C4**	-17.21	-42.90	-77.01	-	-	-	-	-	-	-	-	-	-	-	-	-	-
**C5**	11.96	21.88	27.73	-77.01	-	-	-	-	-	-	-	-	-	-	-	-	-
**C6**	18.33	23.56	21.88	-42.90	42.32	-	-	-	-	-	-	-	-	-	-	-	-
**H7**	-13.15	-14.09	-9.51	14.03	-9.51	-14.09	-	-	-	-	-	-	-	-	-	-	-
**H8**	-6.85	-28.95	-15.84	16.48	-8.69	-9.09	6.45	-	-	-	-	-	-	-	-	-	-
**H9**	-6.87	-28.30	-15.66	20.11	-10.06	-11.13	6.52	9.32	-	-	-	-	-	-	-	-	-
**H10**	-4.33	-14.79	-32.05	27.26	-10.12	-8.19	3.82	6.72	6.79	-	-	-	-	-	-	-	-
**H11**	-5.30	-14.83	-32.02	27.32	-12.44	-9.53	4.29	6.84	5.47	9.82	-	-	-	-	-	-	-
**F12**	5.96	14.37	24.51	-70.03	24.51	14.37	-5.21	-6.25	-6.87	-11.04	-11.16	-	-	-	-	-	-
**F13**	7.50	19.08	25.69	-72.60	25.69	19.08	-6.71	-7.54	-10.99	-11.77	-9.17	22.68	-	-	-	-	-
**H14**	-4.33	-8.19	-10.12	27.26	-32.05	-14.79	3.82	3.43	4.05	4.10	4.59	-11.04	-11.77	-	-	-	-
**H15**	-5.30	-9.53	-12.44	27.32	-32.02	-14.83	4.29	4.07	4.16	4.59	6.57	-11.16	-9.17	9.82	-	-	-
**H16**	-6.85	-9.09	-8.69	16.48	-15.84	-28.95	6.45	3.89	4.35	3.43	4.07	-6.25	-7.54	6.72	6.84	-	-
**H17**	-6.87	-11.13	-10.06	20.11	-15.66	-28.30	6.52	4.35	6.22	4.05	4.16	-6.87	-10.99	6.79	5.47	9.32	-
**Br18**	1.50	2.19	2.03	-2.88	2.03	2.19	-1.31	-1.12	-0.86	-0.78	-1.15	1.16	1.19	-0.78	-1.15	-1.12	-0.86

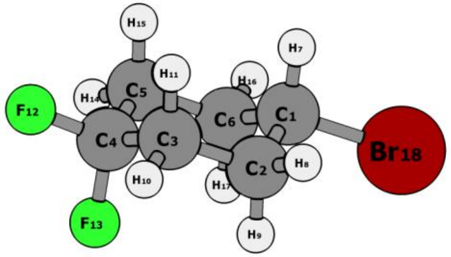

Atom Numbering

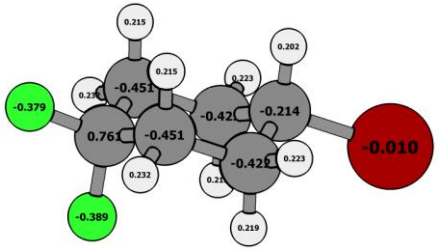

NPA charges

Table S23. Atom-atom electrostatic interactions (kcal mol^{-1}) obtained at M06-2X/aug-cc-pVTZ level using NPA charges for 12$_{eq}$.

	C1	C2	C3	C4	C5	C6	H7	H8	H9	H10	H11	F12	F13	H14	H15	H16	H17
**C2**	19.75	-	-	-	-	-	-	-	-	-	-	-	-	-	-	-	-
**C3**	12.85	41.32	-	-	-	-	-	-	-	-	-	-	-	-	-	-	-
**C4**	-18.70	-42.63	-75.38	-	-	-	-	-	-	-	-	-	-	-	-	-	-
**C5**	12.85	21.41	26.68	-75.38	-	-	-	-	-	-	-	-	-	-	-	-	-
**C6**	19.75	23.49	21.41	-42.62	41.32	-	-	-	-	-	-	-	-	-	-	-	-
**H7**	-13.22	-13.15	-10.90	15.49	-10.90	-13.15	-	-	-	-	-	-	-	-	-	-	-
**H8**	-7.34	-28.65	-15.41	16.31	-8.45	-9.01	6.04	-	-	-	-	-	-	-	-	-	-
**H9**	-7.27	-28.08	-15.17	19.85	-9.81	-11.09	4.82	9.19	-	-	-	-	-	-	-	-	-
**H10**	-4.77	-14.93	-31.87	27.58	-10.06	-8.28	4.11	6.76	6.78	-	-	-	-	-	-	-	-
**H11**	-5.48	-13.85	-29.45	25.63	-11.45	-8.92	5.49	6.36	5.08	9.36	-	-	-	-	-	-	-
**F12**	6.51	14.35	24.10	-70.04	24.10	14.35	-5.88	-6.21	-6.81	-11.20	-10.56	-	-	-	-	-	-
**F13**	7.94	18.67	24.84	-71.67	24.84	18.67	-6.22	-7.36	-10.64	-11.76	-8.50	22.41	-	-	-	-	-
**H14**	-4.77	-8.28	-10.06	27.57	-31.87	-14.93	4.11	3.45	4.08	4.21	4.37	-11.20	-11.76	-	-	-	-
**H15**	-5.48	-8.92	-11.45	25.63	-29.45	-13.85	5.49	3.78	3.87	4.37	5.79	-10.56	-8.50	9.36	-	-	-
**H16**	-7.34	-9.01	-8.45	16.31	-15.41	-28.65	6.04	3.83	4.31	3.45	3.78	-6.21	-7.36	6.76	6.36	-	-
**H17**	-7.27	-11.09	-9.81	19.85	-15.17	-28.08	4.82	4.31	6.20	4.08	3.87	-6.81	-10.64	6.78	5.08	9.19	-
**Br18**	0.38	0.51	0.36	-0.55	0.36	0.51	-0.28	-0.25	-0.25	-0.16	-0.16	0.22	0.27	-0.16	-0.16	-0.25	-0.25

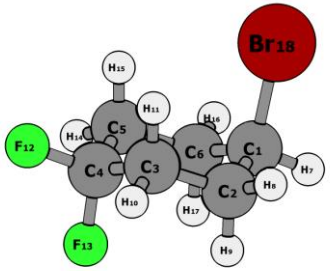

Atom Numbering

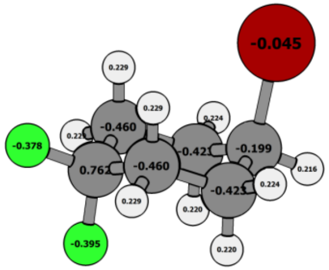

NPA charges

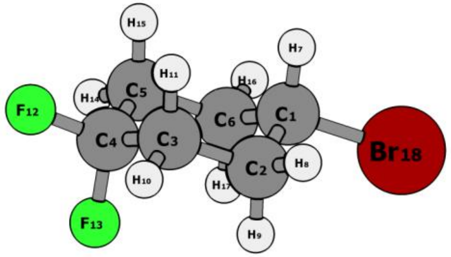

Atom Numbering

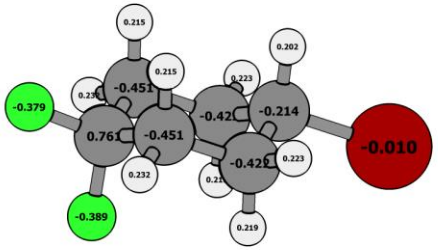

NPA charges

Table S24. $\Delta_{12ax-12eq}$ **Atom-atom electrostatic interactions (kcal mol^{-1}) obtained at M06-2X/aug-cc-pVTZ level using NPA charges.**

	C1	C2	C3	C4	C5	C6	H7	H8	H9	H10	H11	F12	F13	H14	H15	H16	H17
**C2**	-1.42	-	-	-	-	-	-	-	-	-	-	-	-	-	-	-	-
**C3**	-0.89	1.00	-	-	-	-	-	-	-	-	-	-	-	-	-	-	-
**C4**	1.49	-0.27	-1.63	-	-	-	-	-	-	-	-	-	-	-	-	-	-
**C5**	-0.89	0.47	1.05	-1.63	-	-	-	-	-	-	-	-	-	-	-	-	-
**C6**	-1.42	0.07	0.47	-0.28	1.00	-	-	-	-	-	-	-	-	-	-	-	-
**H7**	0.07	-0.94	1.39	-1.46	1.39	-0.94	-	-	-	-	-	-	-	-	-	-	-
**H8**	0.49	-0.30	-0.43	0.17	-0.24	-0.08	0.41	-	-	-	-	-	-	-	-	-	-
**H9**	0.40	-0.22	-0.49	0.26	-0.25	-0.04	1.70	0.13	-	-	-	-	-	-	-	-	-
**H10**	0.44	0.14	-0.18	-0.32	-0.06	0.09	-0.29	-0.04	0.01	-	-	-	-	-	-	-	-
**H11**	0.18	-0.98	-2.57	1.69	-0.99	-0.61	-1.20	0.48	0.39	0.46	-	-	-	-	-	-	-
**F12**	-0.55	0.02	0.41	0.01	0.41	0.02	0.67	-0.04	-0.06	0.16	-0.60	-	-	-	-	-	-
**F13**	-0.44	0.41	0.85	-0.93	0.85	0.41	-0.49	-0.18	-0.35	-0.01	-0.67	0.27	-	-	-	-	-
**H14**	0.44	0.09	-0.06	-0.31	-0.18	0.14	-0.29	-0.02	-0.03	-0.11	0.22	0.16	-0.01	-	-	-	-
**H15**	0.18	-0.61	-0.99	1.69	-2.57	-0.98	-1.20	0.29	0.29	0.22	0.78	-0.60	-0.67	0.46	-	-	-
**H16**	0.49	-0.08	-0.24	0.17	-0.43	-0.30	0.41	0.06	0.04	-0.02	0.29	-0.04	-0.18	-0.04	0.48	-	-
**H17**	0.40	-0.04	-0.25	0.26	-0.49	-0.22	1.70	0.04	0.02	-0.03	0.29	-0.06	-0.35	0.01	0.39	0.13	-
**Br18**	1.12	1.68	1.67	-2.33	1.67	1.68	-1.03	-0.87	-0.61	-0.62	-0.99	0.94	0.92	-0.62	-0.99	-0.87	-0.61

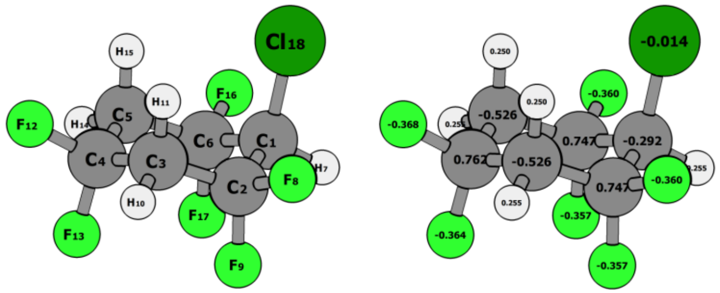

Atom Numbering **NPA charges**

Table S25. Atom-atom electrostatic interactions (kcal mol^{-1}) obtained at M06-2X/aug-cc-pVTZ level using NPA charges for 13_{ax}.

	C1	C2	C3	C4	C5	C6	H7	F8	F9	H10	H11	F12	F13	H14	H15	F16	F17
**C2**	-47.16	-	-	-	-	-	-	-	-	-	-	-	-	-	-	-	-
**C3**	19.93	-86.16	-	-	-	-	-	-	-	-	-	-	-	-	-	-	-
**C4**	-24.89	75.17	-87.65	-	-	-	-	-	-	-	-	-	-	-	-	-	-
**C5**	19.93	-44.18	36.01	-87.65	-	-	-	-	-	-	-	-	-	-	-	-	-
**C6**	-47.16	73.54	-44.18	75.17	-86.16	-	-	-	-	-	-	-	-	-	-	-	-
**H7**	-22.67	29.36	-12.81	16.43	-12.81	29.36	-	-	-	-	-	-	-	-	-	-	-
**F8**	14.77	-65.90	26.81	-24.67	15.02	-24.11	-11.44	-	-	-	-	-	-	-	-	-	-
**F9**	14.82	-65.31	26.45	-30.29	17.67	-30.10	-12.04	19.65	-	-	-	-	-	-	-	-	-
**H10**	-7.10	29.64	-40.89	30.12	-12.83	16.18	5.06	-11.54	-11.85	-	-	-	-	-	-	-	-
**H11**	-8.50	29.23	-40.16	29.72	-15.27	18.36	5.52	-11.77	-9.08	11.93	-	-	-	-	-	-	-
**F12**	8.55	-24.79	27.49	-68.33	27.49	-24.79	-6.00	9.42	10.29	-11.71	-12.16	-	-	-	-	-	-
**F13**	9.76	-29.98	27.01	-67.73	27.01	-29.98	-7.09	10.25	15.20	-12.21	-9.27	20.43	-	-	-	-	-
**H14**	-7.10	16.18	-12.83	30.12	-40.89	29.64	5.06	-5.86	-7.12	5.07	5.50	-11.71	-12.21	-	-	-	-
**H15**	-8.50	18.36	-15.27	29.72	-40.16	29.23	5.52	-6.79	-6.97	5.50	7.57	-12.16	-9.27	11.93	-	-	-
**F16**	14.77	-24.11	15.02	-24.67	26.81	-65.90	-11.44	9.13	10.21	-5.86	-6.79	9.42	10.25	-11.54	-11.77	-	-
**F17**	14.82	-30.10	17.67	-30.29	26.45	-65.31	-12.04	10.21	15.64	-7.12	-6.97	10.29	15.20	-11.85	-9.08	19.65	-
**Cl18**	0.74	-1.24	0.73	-0.90	0.73	-1.24	-0.49	0.56	0.41	-0.27	-0.39	0.36	0.34	-0.27	-0.39	0.56	0.41

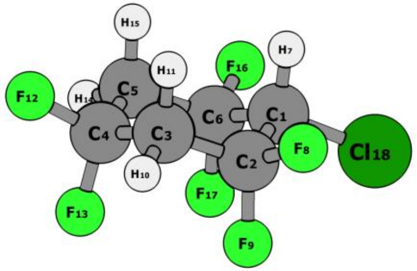

Atom Numbering

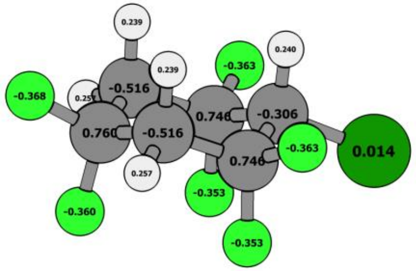

NPA charges

Table S26. Atom-atom electrostatic interactions (kcal mol^{-1}) obtained at M06-2X/aug-cc-pVTZ level using NPA charges for 13$_{eq}$.

	C1	C2	C3	C4	C5	C6	H7	F8	F9	H10	H11	F12	F13	H14	H15	F16	F17
**C2**	-49.57	-	-	-	-	-	-	-	-	-	-	-	-	-	-	-	-
**C3**	20.69	-84.35	-	-	-	-	-	-	-	-	-	-	-	-	-	-	-
**C4**	-26.19	74.78	-86.01	-	-	-	-	-	-	-	-	-	-	-	-	-	-
**C5**	20.69	-43.35	34.90	-86.01	-	-	-	-	-	-	-	-	-	-	-	-	-
**C6**	-49.57	73.17	-43.35	74.78	-84.36	-	-	-	-	-	-	-	-	-	-	-	-
**H7**	-22.42	28.02	-14.81	18.35	-14.81	28.02	-	-	-	-	-	-	-	-	-	-	-
**F8**	15.71	-66.25	26.66	-24.87	14.95	-24.32	-11.42	-	-	-	-	-	-	-	-	-	-
**F9**	15.18	-64.91	25.77	-29.74	17.01	-29.25	-8.63	19.57	-	-	-	-	-	-	-	-	-
**H10**	-7.54	29.83	-40.45	30.26	-12.72	16.26	5.42	-11.73	-11.90	-	-	-	-	-	-	-	-
**H11**	-8.58	27.79	-37.61	28.33	-14.42	17.53	7.23	-11.44	-8.59	11.49	-	-	-	-	-	-	-
**F12**	9.00	-24.73	27.00	-68.18	27.00	-24.73	-6.87	9.52	10.17	-11.81	-11.60	-	-	-	-	-	-
**F13**	10.19	-29.69	26.28	-67.04	26.28	-29.69	-6.77	10.22	14.81	-12.13	-8.76	20.22	-	-	-	-	-
**H14**	-7.54	16.26	-12.72	30.26	-40.45	29.83	5.42	-5.97	-7.01	5.14	5.31	-11.81	-12.13	-	-	-	-
**H15**	-8.58	17.53	-14.42	28.33	-37.61	27.79	7.23	-6.60	-6.55	5.31	6.98	-11.60	-8.76	11.49	-	-	-
**F16**	15.71	-24.32	14.95	-24.87	26.66	-66.25	-11.42	9.34	10.05	-5.97	-6.60	9.52	10.22	-11.73	-11.44	-	-
**F17**	15.18	-29.25	17.00	-29.74	25.77	-64.91	-8.63	10.05	14.67	-7.01	-6.55	10.17	14.81	-11.90	-8.59	19.57	-
**Cl18**	-0.79	1.25	-0.57	0.75	-0.57	1.25	0.47	-0.55	-0.54	0.24	0.24	-0.29	-0.33	0.24	0.24	-0.55	-0.54

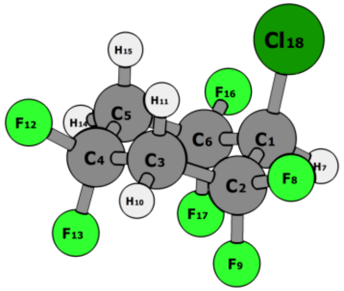

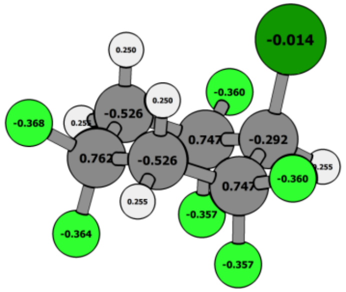

Atom Numbering NPA charges

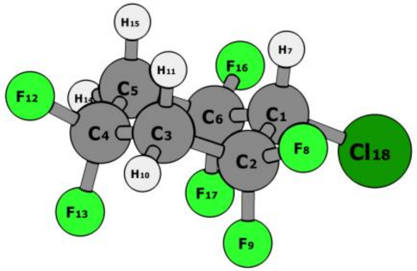

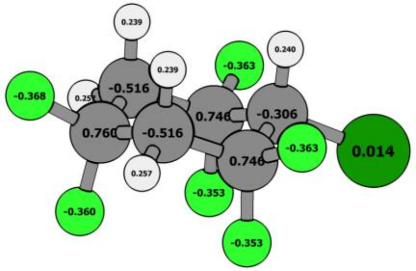

Atom Numbering NPA charges

Table S27. $\Delta_{13ax-13eq}$ **Atom-atom electrostatic interactions (kcal mol^{-1}) obtained at M06-2X/aug-cc-pVTZ level using NPA charges.**

	C1	C2	C3	C4	C5	C6	H7	F8	F9	H10	H11	F12	F13	H14	H15	F16	F17
C2	2.41	-	-	-	-	-	-	-	-	-	-	-	-	-	-	-	-
C3	-0.76	-1.81	-	-	-	-	-	-	-	-	-	-	-	-	-	-	-
C4	1.30	0.39	-1.64	-	-	-	-	-	-	-	-	-	-	-	-	-	-
C5	-0.76	-0.83	1.11	-1.64	-	-	-	-	-	-	-	-	-	-	-	-	-
C6	2.41	0.37	-0.83	0.39	-1.80	-	-	-	-	-	-	-	-	-	-	-	-
H7	-0.25	1.34	2.00	-1.92	2.00	1.34	-	-	-	-	-	-	-	-	-	-	-
F8	-0.94	0.35	0.15	0.20	0.07	0.21	-0.02	-	-	-	-	-	-	-	-	-	-
F9	-0.36	-0.40	0.68	-0.55	0.66	-0.85	-3.41	0.08	-	-	-	-	-	-	-	-	-
H10	0.44	-0.19	-0.44	-0.14	-0.11	-0.08	-0.36	0.19	0.05	-	-	-	-	-	-	-	-
H11	0.08	1.44	-2.55	1.39	-0.85	0.83	-1.71	-0.33	-0.49	0.44	-	-	-	-	-	-	-
F12	-0.45	-0.06	0.49	-0.15	0.49	-0.06	0.87	-0.10	0.12	0.10	-0.56	-	-	-	-	-	-
F13	-0.43	-0.29	0.73	-0.69	0.73	-0.29	-0.32	0.03	0.39	-0.08	-0.51	0.21	-	-	-	-	-
H14	0.44	-0.08	-0.11	-0.14	-0.44	-0.19	-0.36	0.11	-0.11	-0.07	0.19	0.10	-0.08	-	-	-	-
H15	0.08	0.83	-0.85	1.39	-2.55	1.44	-1.71	-0.19	-0.42	0.19	0.59	-0.56	-0.51	0.44	-	-	-
F16	-0.94	0.21	0.07	0.20	0.15	0.35	-0.02	-0.21	0.16	0.11	-0.19	-0.10	0.03	0.19	-0.33	-	-
F17	-0.36	-0.85	0.67	-0.55	0.68	-0.40	-3.41	0.16	0.97	-0.11	-0.42	0.12	0.39	0.05	-0.49	0.08	-
Cl18	1.53	-2.49	1.30	-1.65	1.30	-2.49	-0.96	1.11	0.95	-0.51	-0.63	0.65	0.67	-0.51	-0.63	1.11	0.95

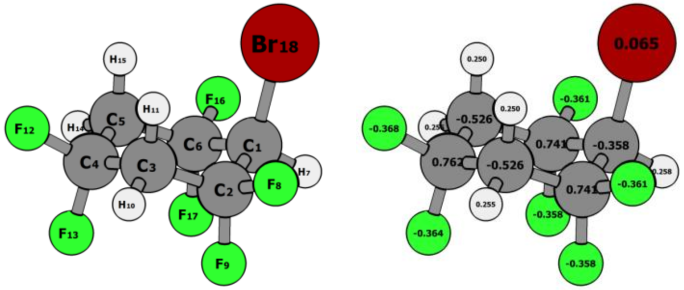

Atom Numbering **NPA charges**

Table S28. Atom-atom electrostatic interactions (kcal mol^{-1}) obtained at M06-2X/aug-cc-pVTZ level using NPA charges for 14$_{ax}$.

	C1	C2	C3	C4	C5	C6	H7	F8	F9	H10	H11	F12	F13	H14	H15	F16	F17
**C2**	-57.42	-	-	-	-	-	-	-	-	-	-	-	-	-	-	-	-
**C3**	24.41	-85.49	-	-	-	-	-	-	-	-	-	-	-	-	-	-	-
**C4**	-30.49	74.59	-87.68	-	-	-	-	-	-	-	-	-	-	-	-	-	-
**C5**	24.41	-43.85	36.02	-87.67	-	-	-	-	-	-	-	-	-	-	-	-	-
**C6**	-57.42	72.46	-43.86	74.59	-85.49	-	-	-	-	-	-	-	-	-	-	-	-
**H7**	-28.23	29.57	-12.99	16.68	-12.99	29.57	-	-	-	-	-	-	-	-	-	-	-
**F8**	18.15	-65.64	26.92	-24.76	15.07	-24.00	-11.62	-	-	-	-	-	-	-	-	-	-
**F9**	18.23	-64.90	26.54	-30.39	17.72	-29.95	-12.27	19.79	-	-	-	-	-	-	-	-	-
**H10**	-8.71	29.43	-40.90	30.13	-12.84	16.07	5.14	-11.60	-11.90	-	-	-	-	-	-	-	-
**H11**	-10.39	28.95	-40.11	29.68	-15.25	18.19	5.58	-11.78	-9.09	11.92	-	-	-	-	-	-	-
**F12**	10.47	-24.61	27.50	-68.35	27.50	-24.61	-6.09	9.45	10.33	-11.71	-12.16	-	-	-	-	-	-
**F13**	11.96	-29.73	27.00	-67.72	27.00	-29.73	-7.20	10.29	15.24	-12.22	-9.25	20.43	-	-	-	-	-
**H14**	-8.71	16.07	-12.84	30.13	-40.90	29.43	5.14	-5.88	-7.14	5.07	5.49	-11.71	-12.22	-	-	-	-
**H15**	-10.39	18.19	-15.25	29.68	-40.11	28.95	5.58	-6.80	-6.98	5.49	7.54	-12.16	-9.25	11.92	-	-	-
**F16**	18.15	-24.00	15.07	-24.76	26.91	-65.64	-11.62	9.18	10.27	-5.88	-6.80	9.45	10.29	-11.60	-11.78	-	-
**F17**	18.23	-29.95	17.72	-30.39	26.53	-64.90	-12.27	10.27	15.73	-7.14	-6.98	10.33	15.24	-11.90	-9.09	19.79	-
**Br18**	-3.98	5.60	-3.34	4.15	-3.34	5.60	2.24	-2.57	-1.91	1.26	1.80	-1.67	-1.56	1.26	1.80	-2.57	-1.91

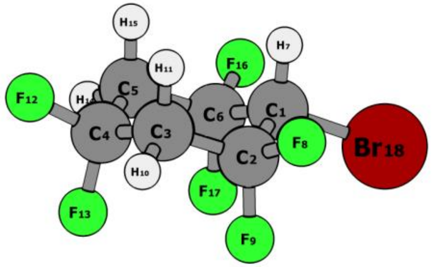

Atom Numbering

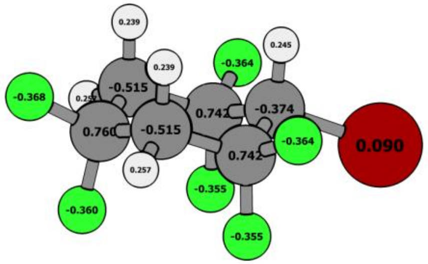

NPA charges

Table S29. Atom-atom electrostatic interactions (kcal mol^{-1}) obtained at M06-2X/aug-cc-pVTZ level using NPA charges for 14_{eq}.

	C1	C2	C3	C4	C5	C6	H7	F8	F9	H10	H11	F12	F13	H14	H15	F16	F17
**C2**	-60.30	-	-	-	-	-	-	-	-	-	-	-	-	-	-	-	-
**C3**	25.25	-83.47	-	-	-	-	-	-	-	-	-	-	-	-	-	-	-
**C4**	-32.03	74.22	-85.71	-	-	-	-	-	-	-	-	-	-	-	-	-	-
**C5**	25.25	-42.93	34.69	-85.71	-	-	-	-	-	-	-	-	-	-	-	-	-
**C6**	-60.30	72.24	-42.93	74.22	-83.47	-	-	-	-	-	-	-	-	-	-	-	-
**H7**	-27.99	28.39	-15.04	18.69	-15.04	28.39	-	-	-	-	-	-	-	-	-	-	-
**F8**	19.25	-65.98	26.66	-24.92	14.94	-24.21	-11.67	-	-	-	-	-	-	-	-	-	-
**F9**	18.64	-64.77	25.82	-29.86	17.02	-29.17	-8.84	19.71	-	-	-	-	-	-	-	-	-
**H10**	-9.22	29.61	-40.30	30.23	-12.67	16.15	5.52	-11.77	-11.96	-	-	-	-	-	-	-	-
**H11**	-10.50	27.59	-37.47	28.30	-14.37	17.41	7.36	-11.47	-8.63	11.47	-	-	-	-	-	-	-
**F12**	11.01	-24.57	26.92	-68.15	26.92	-24.57	-7.00	9.55	10.22	-11.81	-11.60	-	-	-	-	-	-
**F13**	12.47	-29.49	26.20	-67.02	26.20	-29.49	-6.90	10.25	14.88	-12.13	-8.75	20.23	-	-	-	-	-
**H14**	-9.22	16.15	-12.67	30.23	-40.30	29.62	5.52	-5.98	-7.04	5.14	5.31	-11.81	-12.13	-	-	-	-
**H15**	-10.50	17.41	-14.37	28.30	-37.47	27.59	7.36	-6.61	-6.57	5.31	6.97	-11.60	-8.75	11.47	-	-	-
**F16**	19.25	-24.21	14.94	-24.92	26.66	-65.98	-11.67	9.38	10.11	-5.98	-6.61	9.55	10.25	-11.77	-11.47	-	-
**F17**	18.64	-29.17	17.02	-29.86	25.82	-64.77	-8.84	10.11	14.77	-7.04	-6.57	10.22	14.88	-11.96	-8.63	19.71	-
**Br18**	-5.79	7.73	-3.61	4.77	-3.61	7.73	2.96	-3.46	-3.43	1.53	1.55	-1.82	-2.11	1.53	1.55	-3.46	-3.43

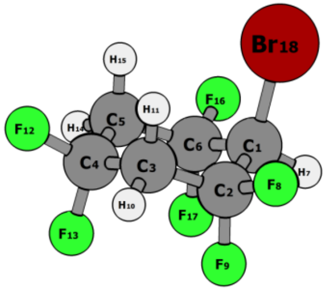

Atom Numbering

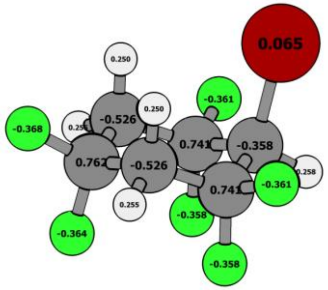

NPA charges

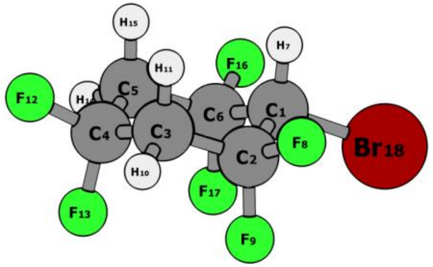

Atom Numbering

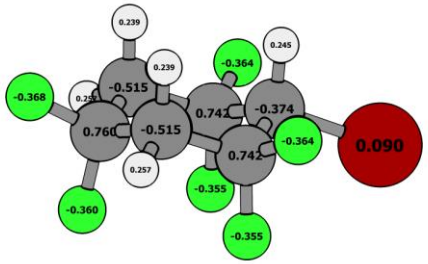

NPA charges

Table S30. $\Delta_{14ax-14eq}$ **Atom-atom electrostatic interactions (kcal mol^{-1}) obtained at M06-2X/aug-cc-pVTZ level using NPA charges.**

	C1	C2	C3	C4	C5	C6	H7	F8	F9	H10	H11	F12	F13	H14	H15	F16	F17
**C2**	2.88	-	-	-	-	-	-	-	-	-	-	-	-	-	-	-	-
**C3**	-0.84	-2.02	-	-	-	-	-	-	-	-	-	-	-	-	-	-	-
**C4**	1.54	0.37	-1.97	-	-	-	-	-	-	-	-	-	-	-	-	-	-
**C5**	-0.84	-0.92	1.33	-1.96	-	-	-	-	-	-	-	-	-	-	-	-	-
**C6**	2.88	0.22	-0.93	0.37	-2.02	-	-	-	-	-	-	-	-	-	-	-	-
**H7**	-0.24	1.18	2.05	-2.01	2.05	1.18	-	-	-	-	-	-	-	-	-	-	-
**F8**	-1.10	0.34	0.26	0.16	0.13	0.21	0.05	-	-	-	-	-	-	-	-	-	-
**F9**	-0.41	-0.13	0.72	-0.53	0.70	-0.78	-3.43	0.08	-	-	-	-	-	-	-	-	-
**H10**	0.51	-0.18	-0.60	-0.10	-0.17	-0.08	-0.38	0.17	0.06	-	-	-	-	-	-	-	-
**H11**	0.11	1.36	-2.64	1.38	-0.88	0.78	-1.78	-0.31	-0.46	0.45	-	-	-	-	-	-	-
**F12**	-0.54	-0.04	0.58	-0.20	0.58	-0.04	0.91	-0.10	0.11	0.10	-0.56	-	-	-	-	-	-
**F13**	-0.51	-0.24	0.80	-0.70	0.80	-0.24	-0.30	0.04	0.36	-0.09	-0.50	0.20	-	-	-	-	-
**H14**	0.51	-0.08	-0.17	-0.10	-0.60	-0.19	-0.38	0.10	-0.10	-0.07	0.18	0.10	-0.09	-	-	-	-
**H15**	0.11	0.78	-0.88	1.38	-2.64	1.36	-1.78	-0.19	-0.41	0.18	0.57	-0.56	-0.50	0.45	-	-	-
**F16**	-1.10	0.21	0.13	0.16	0.25	0.34	0.05	-0.20	0.16	0.10	-0.19	-0.10	0.04	0.17	-0.31	-	-
**F17**	-0.41	-0.78	0.70	-0.53	0.71	-0.13	-3.43	0.16	0.96	-0.10	-0.41	0.11	0.36	0.06	-0.46	0.08	-
**Br18**	1.81	-2.13	0.27	-0.62	0.27	-2.13	-0.72	0.89	1.52	-0.27	0.25	0.15	0.55	-0.27	0.25	0.89	1.52

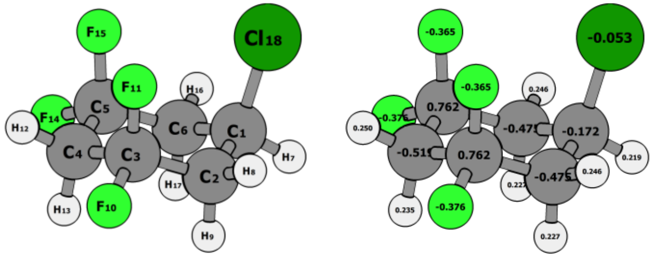

Atom Numbering **NPA charges**

Table S31. Atom-atom electrostatic interactions (kcal mol^{-1}) obtained at M06-2X/aug-cc-pVTZ level using NPA charges for 15$_{ax}$.

	C1	C2	C3	C4	C5	C6	H7	H8	H9	F10	F11	H12	H13	F14	F15	H16	H17
**C2**	17.79	-	-	-	-	-	-	-	-	-	-	-	-	-	-	-	-
**C3**	-17.12	-79.34	-	-	-	-	-	-	-	-	-	-	-	-	-	-	-
**C4**	9.93	32.18	-86.64	-	-	-	-	-	-	-	-	-	-	-	-	-	-
**C5**	-17.12	-40.87	76.79	-86.64	-	-	-	-	-	-	-	-	-	-	-	-	-
**C6**	17.79	29.60	-40.87	32.18	-79.34	-	-	-	-	-	-	-	-	-	-	-	-
**H7**	-11.52	-16.15	16.04	-9.60	16.04	-16.15	-	-	-	-	-	-	-	-	-	-	-
**H8**	-6.49	-35.70	29.29	-12.26	15.92	-11.19	7.06	-	-	-	-	-	-	-	-	-	-
**H9**	-6.09	-32.83	27.31	-13.88	17.20	-12.68	6.93	10.53	-	-	-	-	-	-	-	-	-
**F10**	5.80	25.37	-69.70	27.72	-25.86	14.29	-6.14	-11.73	-11.42	-	-	-	-	-	-	-	-
**F11**	6.83	24.44	-68.17	26.76	-30.62	16.05	-6.58	-11.77	-8.48	21.00	-	-	-	-	-	-	-
**H12**	-3.62	-11.39	29.59	-39.61	29.59	-11.39	3.68	4.82	4.96	-11.77	-12.03	-	-	-	-	-	-
**H13**	-3.95	-13.03	28.02	-37.15	28.02	-13.03	4.12	5.03	6.60	-11.72	-8.75	11.01	-	-	-	-	-
**F14**	5.80	14.29	-25.86	27.72	-69.70	25.37	-6.14	-5.93	-6.57	10.10	10.70	-11.77	-11.72	-	-	-	-
**F15**	6.83	16.05	-30.62	26.76	-68.17	24.44	-6.58	-6.87	-6.44	10.70	15.30	-12.03	-8.75	21.00	-	-	-
**H16**	-6.49	-11.19	15.92	-12.26	29.28	-35.70	7.06	4.69	4.85	-5.93	-6.87	4.82	5.03	-11.73	-11.77	-	-
**H17**	-6.09	-12.68	17.20	-13.87	27.31	-32.83	6.93	4.85	6.38	-6.57	-6.43	4.96	6.60	-11.42	-8.48	10.53	-
**Cl18**	1.70	3.07	-4.01	2.32	-4.01	3.07	-1.68	-1.54	-1.10	1.45	2.09	-0.96	-0.88	1.45	2.09	-1.54	-1.10

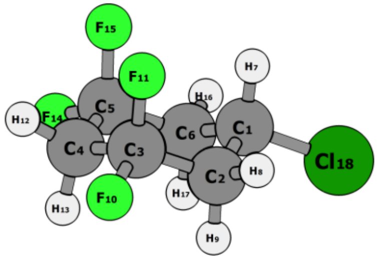

Atom Numbering

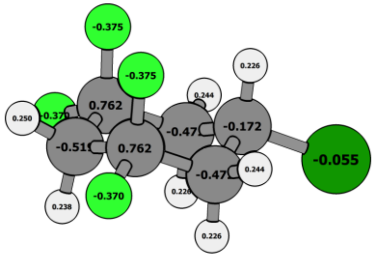

NPA charges

Table S32. Atom-atom electrostatic interactions (kcal mol^{-1}) obtained at M06-2X/aug-cc-pVTZ level using NPA charges for 15$_{eq}$.

	C1	C2	C3	C4	C5	C6	H7	H8	H9	F10	F11	H12	H13	F14	F15	H16	H17
**C2**	17.74	-	-	-	-	-	-	-	-	-	-	-	-	-	-	-	-
**C3**	-17.52	-78.88	-	-	-	-	-	-	-	-	-	-	-	-	-	-	-
**C4**	10.08	31.97	-86.66	-	-	-	-	-	-	-	-	-	-	-	-	-	-
**C5**	-17.52	-40.78	77.00	-86.66	-	-	-	-	-	-	-	-	-	-	-	-	-
**C6**	17.74	29.47	-40.78	31.97	-78.88	-	-	-	-	-	-	-	-	-	-	-	-
**H7**	-11.88	-16.45	20.65	-11.71	20.65	-16.45	-	-	-	-	-	-	-	-	-	-	-
**H8**	-6.43	-35.11	29.01	-12.14	15.77	-11.03	7.38	-	-	-	-	-	-	-	-	-	-
**H9**	-6.01	-32.52	26.91	-13.78	17.29	-12.83	5.56	10.35	-	-	-	-	-	-	-	-	-
**F10**	5.75	24.71	-68.83	27.25	-25.47	13.98	-6.89	-11.47	-10.87	-	-	-	-	-	-	-	-
**F11**	7.34	25.06	-69.55	27.45	-31.66	16.53	-10.57	-11.97	-8.62	21.14	-	-	-	-	-	-	-
**H12**	-3.68	-11.30	29.53	-39.56	29.53	-11.30	4.62	4.76	4.91	-11.57	-12.28	-	-	-	-	-	-
**H13**	-4.00	-13.10	28.39	-37.69	28.39	-13.10	4.42	5.05	6.62	-11.62	-9.08	11.14	-	-	-	-	-
**F14**	5.75	13.98	-25.47	27.25	-68.83	24.71	-6.89	-5.78	-6.46	9.76	10.86	-11.57	-11.62	-	-	-	-
**F15**	7.34	16.53	-31.66	27.45	-69.55	25.06	-10.57	-7.02	-6.64	10.86	16.42	-12.28	-9.08	21.14	-	-	-
**H16**	-6.43	-11.03	15.77	-12.14	29.01	-35.11	7.38	4.58	4.86	-5.78	-7.02	4.76	5.05	-11.47	-11.97	-	-
**H17**	-6.01	-12.83	17.29	-13.78	26.91	-32.52	5.56	4.86	6.61	-6.46	-6.64	4.91	6.62	-10.87	-8.62	10.35	-
**Cl18**	1.75	3.17	-3.40	2.04	-3.40	3.17	-1.76	-1.52	-1.43	1.33	1.50	-0.81	-0.89	1.33	1.50	-1.52	-1.43

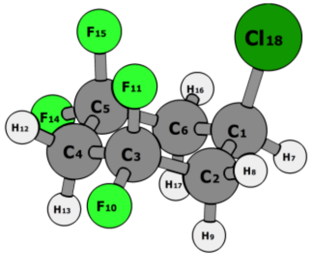

Atom Numbering

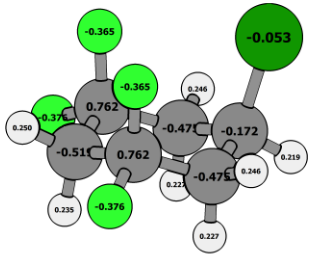

NPA charges

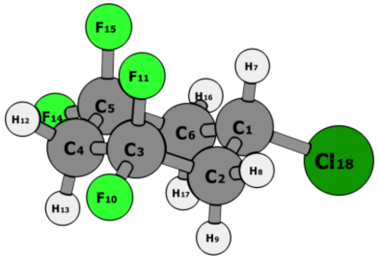

Atom Numbering

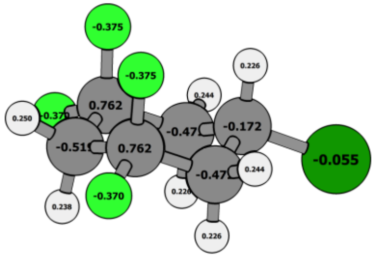

NPA charges

Table S33. $\Delta_{15ax\text{-}15eq}$ **Atom-atom electrostatic interactions (kcal mol^{-1}) obtained at M06-2X/aug-cc-pVTZ level using NPA charges.**

	C1	C2	C3	C4	C5	C6	H7	H8	H9	F10	F11	H12	H13	F14	F15	H16	H17
**C2**	0.05	-	-	-	-	-	-	-	-	-	-	-	-	-	-	-	-
**C3**	0.40	-0.46	-	-	-	-	-	-	-	-	-	-	-	-	-	-	-
**C4**	-0.15	0.21	0.02	-	-	-	-	-	-	-	-	-	-	-	-	-	-
**C5**	0.40	-0.09	-0.21	0.02	-	-	-	-	-	-	-	-	-	-	-	-	-
**C6**	0.05	0.13	-0.09	0.21	-0.46	-	-	-	-	-	-	-	-	-	-	-	-
**H7**	0.36	0.30	-4.61	2.11	-4.61	0.30	-	-	-	-	-	-	-	-	-	-	-
**H8**	-0.06	-0.59	0.28	-0.12	0.15	-0.16	-0.32	-	-	-	-	-	-	-	-	-	-
**H9**	-0.08	-0.31	0.40	-0.10	-0.09	0.15	1.37	0.18	-	-	-	-	-	-	-	-	-
**F10**	0.05	0.66	-0.87	0.47	-0.39	0.31	0.75	-0.26	-0.55	-	-	-	-	-	-	-	-
**F11**	-0.51	-0.62	1.38	-0.69	1.04	-0.48	3.99	0.20	0.14	-0.14	-	-	-	-	-	-	-
**H12**	0.06	-0.09	0.06	-0.05	0.06	-0.09	-0.94	0.06	0.05	-0.20	0.25	-	-	-	-	-	-
**H13**	0.05	0.07	-0.37	0.54	-0.37	0.07	-0.30	-0.02	-0.02	-0.10	0.33	-0.13	-	-	-	-	-
**F14**	0.05	0.31	-0.39	0.47	-0.87	0.66	0.75	-0.15	-0.11	0.34	-0.16	-0.20	-0.10	-	-	-	-
**F15**	-0.51	-0.48	1.04	-0.69	1.38	-0.62	3.99	0.15	0.20	-0.16	-1.12	0.25	0.33	-0.14	-	-	-
**H16**	-0.06	-0.16	0.15	-0.12	0.27	-0.59	-0.32	0.11	-0.01	-0.15	0.15	0.06	-0.02	-0.26	0.20	-	-
**H17**	-0.08	0.15	-0.09	-0.09	0.40	-0.31	1.37	-0.01	-0.23	-0.11	0.21	0.05	-0.02	-0.55	0.14	0.18	-
**Cl18**	-0.05	-0.10	-0.61	0.28	-0.61	-0.10	0.08	-0.02	0.33	0.12	0.59	-0.15	0.01	0.12	0.59	-0.02	0.33

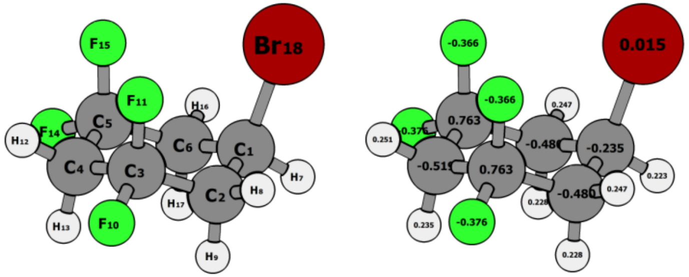

Atom Numbering **NPA charges**

Table S34. Atom-atom electrostatic interactions (kcal mol^{-1}) obtained at M06-2X/aug-cc-pVTZ level using NPA charges for 16_{ax}.

	C1	C2	C3	C4	C5	C6	H7	H8	H9	F10	F11	H12	H13	F14	F15	H16	H17
**C2**	24.64	-	-	-	-	-	-	-	-	-	-	-	-	-	-	-	-
**C3**	-23.42	-80.27	-	-	-	-	-	-	-	-	-	-	-	-	-	-	-
**C4**	13.59	32.56	-86.70	-	-	-	-	-	-	-	-	-	-	-	-	-	-
**C5**	-23.42	-41.35	76.82	-86.70	-	-	-	-	-	-	-	-	-	-	-	-	-
**C6**	24.64	30.29	-41.35	32.56	-80.27	-	-	-	-	-	-	-	-	-	-	-	-
**H7**	-16.06	-16.63	16.33	-9.77	16.33	-16.63	-	-	-	-	-	-	-	-	-	-	-
**H8**	-8.88	-36.21	29.36	-12.30	15.95	-11.33	7.19	-	-	-	-	-	-	-	-	-	-
**H9**	-8.35	-33.24	27.39	-13.92	17.25	-12.86	7.06	10.57	-	-	-	-	-	-	-	-	-
**F10**	7.93	25.65	-69.67	27.71	-25.85	14.44	-6.25	-11.76	-11.44	-	-	-	-	-	-	-	-
**F11**	9.35	24.74	-68.25	26.80	-30.64	16.24	-6.69	-11.80	-8.50	21.01	-	-	-	-	-	-	-
**H12**	-4.96	-11.53	29.62	-39.64	29.62	-11.53	3.75	4.83	4.97	-11.76	-12.06	-	-	-	-	-	-
**H13**	-5.41	-13.19	28.07	-37.22	28.07	-13.19	4.20	5.05	6.63	-11.74	-8.78	11.04	-	-	-	-	-
**F14**	7.93	14.44	-25.85	27.71	-69.67	25.65	-6.25	-5.94	-6.58	10.09	10.69	-11.76	-11.74	-	-	-	-
**F15**	9.35	16.24	-30.64	26.80	-68.25	24.74	-6.69	-6.89	-6.45	10.69	15.31	-12.06	-8.78	21.01	-	-	-
**H16**	-8.88	-11.33	15.95	-12.30	29.36	-36.21	7.19	4.70	4.88	-5.94	-6.89	4.83	5.05	-11.76	-11.80	-	-
**H17**	-8.35	-12.86	17.25	-13.92	27.39	-33.24	7.06	4.88	6.42	-6.58	-6.45	4.97	6.63	-11.44	-8.50	10.57	-
**Br18**	-0.58	-0.80	1.05	-0.61	1.05	-0.80	0.44	0.40	0.29	-0.38	-0.55	0.25	0.23	-0.38	-0.55	0.40	0.29

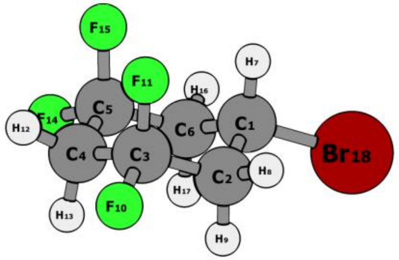

Atom Numbering

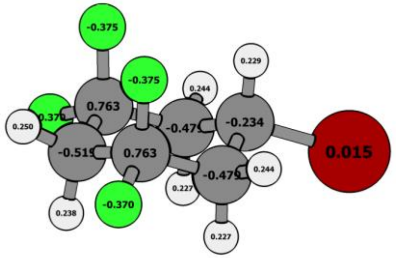

NPA charges

Table S35. Atom-atom electrostatic interactions (kcal mol^{-1}) obtained at M06-2X/aug-cc-pVTZ level using NPA charges for 16$_{eq}$.

	C1	C2	C3	C4	C5	C6	H7	H8	H9	F10	F11	H12	H13	F14	F15	H16	H17
**C2**	24.52	-	-	-	-	-	-	-	-	-	-	-	-	-	-	-	-
**C3**	-23.92	-80.03	-	-	-	-	-	-	-	-	-	-	-	-	-	-	-
**C4**	13.75	32.44	-86.86	-	-	-	-	-	-	-	-	-	-	-	-	-	-
**C5**	-23.92	-41.39	77.20	-86.86	-	-	-	-	-	-	-	-	-	-	-	-	-
**C6**	24.52	30.30	-41.39	32.44	-80.03	-	-	-	-	-	-	-	-	-	-	-	-
**H7**	-16.41	-16.89	20.92	-11.87	20.92	-16.89	-	-	-	-	-	-	-	-	-	-	-
**H8**	-8.77	-35.69	29.12	-12.18	15.82	-11.21	7.49	-	-	-	-	-	-	-	-	-	-
**H9**	-8.19	-33.01	26.97	-13.81	17.32	-13.00	5.64	10.39	-	-	-	-	-	-	-	-	-
**F10**	7.84	25.06	-68.92	27.27	-25.50	14.18	-6.98	-11.51	-10.89	-	-	-	-	-	-	-	-
**F11**	10.01	25.40	-69.63	27.46	-31.70	16.76	-10.70	-12.00	-8.63	21.14	-	-	-	-	-	-	-
**H12**	-5.02	-11.46	29.57	-39.60	29.57	-11.46	4.68	4.77	4.91	-11.57	-12.28	-	-	-	-	-	-
**H13**	-5.45	-13.28	28.43	-37.73	28.43	-13.28	4.48	5.06	6.63	-11.62	-9.08	11.15	-	-	-	-	-
**F14**	7.84	14.18	-25.50	27.27	-68.92	25.06	-6.98	-5.79	-6.46	9.76	10.86	-11.57	-11.62	-	-	-	-
**F15**	10.01	16.76	-31.70	27.46	-69.63	25.40	-10.70	-7.04	-6.64	10.86	16.42	-12.28	-9.08	21.14	-	-	-
**H16**	-8.77	-11.21	15.82	-12.18	29.12	-35.69	7.49	4.60	4.87	-5.79	-7.04	4.77	5.06	-11.51	-12.00	-	-
**H17**	-8.19	-13.00	17.32	-13.81	26.97	-33.01	5.64	4.87	6.61	-6.46	-6.64	4.91	6.63	-10.89	-8.63	10.39	-
**Br18**	-0.59	-0.82	0.88	-0.53	0.88	-0.82	0.45	0.39	0.37	-0.35	-0.39	0.21	0.23	-0.35	-0.39	0.39	0.37

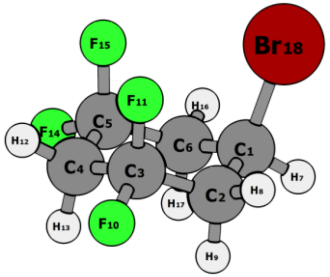

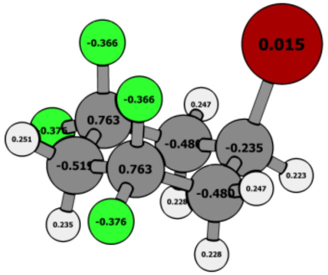

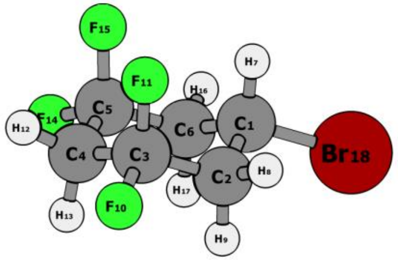

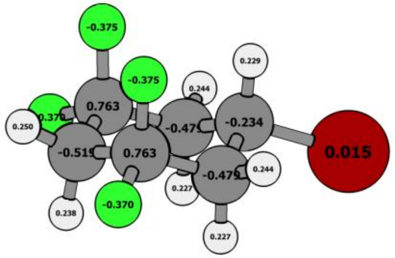

Atom Numbering **NPA charges** **Atom Numbering** **NPA charges**

Table S36. $\Delta_{16ax\text{-}16eq}$ **Atom-atom electrostatic interactions (kcal mol^{-1}) obtained at M06-2X/aug-cc-pVTZ level using NPA charges.**

	C1	C2	C3	C4	C5	C6	H7	H8	H9	F10	F11	H12	H13	F14	F15	H16	H17
**C2**	0.12	-	-	-	-	-	-	-	-	-	-	-	-	-	-	-	-
**C3**	0.50	-0.24	-	-	-	-	-	-	-	-	-	-	-	-	-	-	-
**C4**	-0.16	0.12	0.16	-	-	-	-	-	-	-	-	-	-	-	-	-	-
**C5**	0.50	0.04	-0.38	0.16	-	-	-	-	-	-	-	-	-	-	-	-	-
**C6**	0.12	-0.01	0.04	0.12	-0.24	-	-	-	-	-	-	-	-	-	-	-	-
**H7**	0.35	0.26	-4.59	2.10	-4.59	0.26	-	-	-	-	-	-	-	-	-	-	-
**H8**	-0.11	-0.52	0.24	-0.12	0.13	-0.12	-0.30	-	-	-	-	-	-	-	-	-	-
**H9**	-0.16	-0.23	0.42	-0.11	-0.07	0.14	1.42	0.18	-	-	-	-	-	-	-	-	-
**F10**	0.09	0.59	-0.75	0.44	-0.35	0.26	0.73	-0.25	-0.55	-	-	-	-	-	-	-	-
**F11**	-0.66	-0.66	1.38	-0.66	1.06	-0.52	4.01	0.20	0.13	-0.13	-	-	-	-	-	-	-
**H12**	0.06	-0.07	0.05	-0.04	0.05	-0.07	-0.93	0.06	0.06	-0.19	0.22	-	-	-	-	-	-
**H13**	0.04	0.09	-0.36	0.51	-0.36	0.09	-0.28	-0.01	0.00	-0.12	0.30	-0.11	-	-	-	-	-
**F14**	0.09	0.26	-0.35	0.44	-0.75	0.59	0.73	-0.15	-0.12	0.33	-0.17	-0.19	-0.12	-	-	-	-
**F15**	-0.66	-0.52	1.06	-0.66	1.38	-0.66	4.01	0.15	0.19	-0.17	-1.11	0.22	0.30	-0.13	-	-	-
**H16**	-0.11	-0.12	0.13	-0.12	0.24	-0.52	-0.30	0.10	0.01	-0.15	0.15	0.06	-0.01	-0.25	0.20	-	-
**H17**	-0.16	0.14	-0.07	-0.11	0.42	-0.23	1.42	0.01	-0.19	-0.12	0.19	0.06	0.00	-0.55	0.13	0.18	-
**Br18**	0.01	0.02	0.17	-0.08	0.17	0.02	-0.01	0.01	-0.08	-0.03	-0.16	0.04	0.00	-0.03	-0.16	0.01	-0.08

Table S37. Cartesian coordinates of the optimized geometries for the axial and equatorial geometries of compounds **7-16** obtained at the M06-2X/aug-cc-pVTZ level in the gas phase.

7_{ax} Energy (Hartree) = -533.623015 LHVF (cm^{-1}) = 110.02				7_{eq} Energy (Hartree) = -533.621262 LHVF (cm^{-1}) = 102.58			
C	-1.72318	-0.00006	0.35438	C	-1.61102	-0.00001	-0.36040
C	-0.90570	-1.26222	0.56049	C	-0.89571	-1.25545	0.10189
C	0.34348	-1.26454	-0.31905	C	0.54082	-1.26736	-0.41580
C	1.14016	0.00001	-0.10519	C	1.26025	0.00001	-0.01258
C	0.34341	1.26451	-0.31899	C	0.54079	1.26753	-0.41572
C	-0.90577	1.26222	0.56068	C	-0.89581	1.25551	0.10218
H	-2.60116	-0.00016	1.00131	H	-1.70446	-0.00016	-1.45117
H	-1.52384	-2.13190	0.34151	H	-1.43507	-2.13648	-0.24386
H	-0.62008	-1.31545	1.61266	H	-0.89973	-1.26929	1.19370
H	0.98526	-2.11677	-0.09987	H	1.10077	-2.11742	-0.02794
H	0.06493	-1.30561	-1.37204	H	0.56004	-1.32081	-1.50628
F	2.23279	0.00008	-0.92314	F	2.51927	0.00020	-0.54089
F	1.63822	-0.00002	1.17657	F	1.42838	-0.00024	1.34809
H	0.98522	2.11673	-0.09982	H	1.10065	2.11704	-0.02767
H	0.06479	1.30568	-1.37194	H	0.56016	1.32094	-1.50568
H	-1.52388	2.13177	0.34151	H	-1.43516	2.13600	-0.24358
H	-0.61997	1.31522	1.61270	H	-0.90004	1.26893	1.19342
F	-2.20052	0.00007	-0.96010	F	-2.90132	0.00004	0.15076

8_{ax} Energy (Hartree) = -335.097604 LHVF (cm^{-1}) = 152.78				8_{eq} Energy (Hartree) = -335.097737 LHVF (cm^{-1}) = 163.90			
C	-1.163568	0.000329	0.398866	C	1.021002	0.000027	0.322858
C	-0.343850	-1.262535	0.588277	C	0.342429	-1.255339	-0.183616
C	0.904749	-1.256561	-0.291127	C	-1.132870	-1.258534	0.215760
C	1.738790	-0.000588	-0.049279	C	-1.839472	0.000013	-0.283308
C	0.905904	1.256206	-0.291278	C	-1.132759	1.258567	0.216148
C	-0.343259	1.263089	0.587817	C	0.342513	1.255463	-0.184063
H	-2.032369	0.001130	1.059624	H	1.037434	0.000840	1.417740
H	-0.971133	-2.128484	0.373904	H	0.860095	-2.132542	0.205431
H	-0.056500	-1.319771	1.642238	H	0.434683	-1.273318	-1.273330
H	1.495546	-2.153198	-0.101931	H	-1.621272	-2.152305	-0.172425
H	0.599783	-1.289264	-1.339513	H	-1.211889	-1.305382	1.306175
H	2.618526	-0.001225	-0.693711	H	-2.882270	-0.000262	0.035749
H	2.101353	-0.000648	0.983989	H	-1.840563	0.000210	-1.377417
H	1.496882	2.152569	-0.101595	H	-1.620942	2.152815	-0.171029
H	0.601444	1.289153	-1.339905	H	-1.211193	1.304619	1.306773
H	-0.970088	2.129027	0.372957	H	0.860471	2.132573	0.204343
H	-0.056603	1.320883	1.641789	H	0.434348	1.273723	-1.273695
F	-1.668826	0.000022	-0.906389	F	2.350671	-0.000239	-0.092332

9_{ax} Energy (Hartree) = -930.639495 LHVF (cm^{-1}) = 95.08				9_{eq} Energy (Hartree) = -930.635012 LHVF (cm^{-1}) = 82.97			
C	1.39165	0.00078	-0.14437	C	1.24520	0.00018	-0.53154
C	0.58539	1.26185	0.16312	C	0.51134	1.26358	-0.08840
C	-0.77285	1.27357	-0.50504	C	-0.94602	1.27079	-0.50486
C	-1.55037	-0.00084	-0.23230	C	-1.66529	-0.00022	-0.09446
C	-0.77158	-1.27463	-0.50445	C	-0.94572	-1.27120	-0.50441
C	0.58682	-1.26117	0.16327	C	0.51175	-1.26345	-0.08836
H	2.32694	0.00117	0.41557	H	1.29650	0.00015	-1.62359
F	1.29952	2.33347	-0.25652	F	1.13440	2.33280	-0.64766
F	0.46377	1.36972	1.50621	F	0.62213	1.40279	1.24913
H	-1.34227	2.13043	-0.14948	H	-1.43808	2.13119	-0.05441
H	-0.61924	1.36073	-1.57972	H	-0.99677	1.36049	-1.58965
F	-2.66376	-0.00150	-1.01554	F	-2.90070	-0.00045	-0.66706
F	-1.98968	-0.00076	1.05248	F	-1.86538	0.00000	1.24609
H	-1.34004	-2.13179	-0.14807	H	-1.43735	-2.13149	-0.05329
H	-0.61815	-1.36247	-1.57910	H	-0.99679	-1.36156	-1.58914
F	1.30223	-2.33191	-0.25684	F	1.13508	-2.33242	-0.64783
F	0.46584	-1.36938	1.50630	F	0.62288	-1.40267	1.24917
F	1.65300	0.00085	-1.49171	F	2.50769	0.00030	-0.02824

10_{ax} Energy (Hartree) = -732.132773 LHVF (cm^{-1}) = 98.05	10_{eq} Energy (Hartree) = -732.136210 LHVF (cm^{-1}) = 112.63

C	-0.00009	1.62078	-0.59169	C	-0.00001	1.55152	0.25787
C	1.26634	0.80478	-0.79844	C	1.25461	0.92234	-0.32198
C	1.25561	-0.50207	-0.03282	C	1.25440	-0.57075	-0.07813
C	0.00010	-1.32547	-0.23664	C	0.00000	-1.27517	-0.55656
C	-1.25551	-0.50224	-0.03287	C	-1.25439	-0.57076	-0.07814
C	-1.26649	0.80462	-0.79857	C	-1.25462	0.92233	-0.32199
H	-0.00008	2.46787	-1.27860	H	-0.00001	1.47041	1.34567
H	2.13843	1.38031	-0.49212	H	2.14955	1.34972	0.12698
H	1.36468	0.56636	-1.85859	H	1.28127	1.09970	-1.39830
F	2.32897	-1.25005	-0.42811	F	2.33170	-1.13226	-0.69312
F	1.43470	-0.26163	1.29143	F	1.41958	-0.79937	1.25686
H	0.00010	-2.16790	0.45297	H	0.00001	-2.30453	-0.20220
H	0.00014	-1.70030	-1.25972	H	0.00001	-1.27060	-1.64590
F	-2.32882	-1.25037	-0.42800	F	-2.33170	-1.13228	-0.69311
F	-1.43457	-0.26166	1.29137	F	-1.41958	-0.79937	1.25686
H	-2.13865	1.38005	-0.49206	H	-2.14956	1.34971	0.12697
H	-1.36455	0.56607	-1.85868	H	-1.28127	1.09968	-1.39831
F	-0.00026	2.14650	0.68808	F	-0.00001	2.89982	-0.05652
**11_{ax}**<br>**Energy (Hartree) = -893.982398**<br>**LHVF (cm^{-1}) = 97.26**				**11_{eq}**<br>**Energy (Hartree) = -893.980918**<br>**LHVF (cm^{-1}) = 86.40**			
C	-1.33048	-0.00019	0.79669	C	-1.22379	0.00004	-0.44033
C	-0.47834	-1.26117	0.82686	C	-0.52245	-1.25761	0.04903
C	0.58394	-1.26580	-0.26937	C	0.93166	-1.26612	-0.42159
C	1.40546	0.00004	-0.21242	C	1.63724	0.00000	0.00618
C	0.58385	1.26582	-0.26902	C	0.93163	1.26627	-0.42152
C	-0.47838	1.26094	0.82738	C	-0.52252	1.25771	0.04934
H	-2.03554	-0.00043	1.62285	H	-1.27654	-0.00007	-1.52896
H	-1.11211	-2.14202	0.74437	H	-1.04370	-2.14215	-0.31323
H	0.01054	-1.29624	1.80379	H	-0.55966	-1.27638	1.13976
H	1.25662	-2.11581	-0.16352	H	1.47609	-2.11697	-0.01326
H	0.11768	-1.31611	-1.25340	H	0.98650	-1.32086	-1.51059
F	2.32247	0.00023	-1.22276	F	2.91357	0.00017	-0.47842
F	2.13789	-0.00011	0.95164	F	1.75778	-0.00025	1.37173
H	1.25653	2.11584	-0.16307	H	1.47606	2.11666	-0.01306
H	0.11748	1.31639	-1.25296	H	0.98656	1.32094	-1.51009
H	-1.11220	2.14170	0.74492	H	-1.04378	2.14189	-0.31287
H	0.01069	1.29550	1.80413	H	-0.55987	1.27607	1.13963
Cl	-2.37467	0.00013	-0.68517	Cl	-2.93379	-0.00001	0.11507
**12_{ax}**<br>**Energy (Hartree) = -3008.016772**<br>**LHVF (cm^{-1}) = 86.03**				**12_{eq}**<br>**Energy (Hartree) = -3008.015270**<br>**LHVF (cm^{-1}) = 78.47**			
C	-0.71836	0.00434	1.06604	C	-0.61698	-0.00000	-0.48849
C	0.12756	-1.25764	0.98396	C	0.07029	-1.25881	0.01607
C	1.04339	-1.26721	-0.23711	C	1.53495	-1.26600	-0.42497
C	1.86513	-0.00118	-0.29016	C	2.23095	-0.00002	0.01801
C	1.04350	1.26530	-0.24727	C	1.53495	1.26602	-0.42495
C	0.12762	1.26559	0.97380	C	0.07027	1.25883	0.01609
H	-1.31376	0.00800	1.97328	H	-0.65922	-0.00001	-1.57690
H	-0.50756	-2.14108	0.98899	H	-0.43977	-2.14466	-0.35735
H	0.73963	-1.28767	1.88982	H	0.01299	-1.28035	1.10561
H	1.72485	-2.11656	-0.21106	H	2.06992	-2.11700	-0.00497
H	0.45781	-1.32456	-1.15487	H	1.61162	-1.32149	-1.51233
F	2.64650	-0.00569	-1.40775	F	3.51708	0.00003	-0.43889
F	2.73836	0.00303	0.77214	F	2.32157	-0.00002	1.38602
H	1.72503	2.11478	-0.22801	H	2.06995	2.11695	-0.00489
H	0.45795	1.31533	-1.16548	H	1.61159	1.32146	-1.51228
H	-0.50747	2.14909	0.97171	H	-0.43981	2.14464	-0.35736
H	0.73969	1.30288	1.87942	H	0.01300	1.28026	1.10561
Br	-2.08322	-0.00147	-0.36340	Br	-2.49556	0.00000	0.06629
**13_{ax}**<br>**Energy (Hartree) = -1290.999323**<br>**LHVF (cm^{-1}) = 82.29**				**13_{eq}**<br>**Energy (Hartree) = -1290.996675**<br>**LHVF (cm^{-1}) = 66.86**			
C	1.20675	0.00000	0.35015	C	1.00582	0.00001	-0.54822
C	0.33564	1.26006	0.41837	C	0.27470	1.26424	-0.09562
C	-0.80156	1.27476	-0.58112	C	-1.18714	1.26883	-0.50309
C	-1.62260	-0.00000	-0.52808	C	-1.90424	0.00000	-0.08770
C	-0.80156	-1.27477	-0.58112	C	-1.18714	-1.26885	-0.50304
C	0.33564	-1.26006	0.41837	C	0.27471	-1.26424	-0.09562
H	1.90308	0.00000	1.18546	H	1.01687	0.00000	-1.63749
F	1.11324	2.35145	0.22889	F	0.88039	2.33830	-0.66208
F	-0.14901	1.35174	1.68109	F	0.38230	1.41247	1.24099
H	-1.44527	2.12760	-0.37327	H	-1.67539	2.12965	-0.04924
H	-0.37961	1.37494	-1.57970	H	-1.24387	1.35815	-1.58744
F	-2.48543	-0.00000	-1.58117	F	-3.14377	-0.00002	-0.65173
F	-2.39114	-0.00000	0.59135	F	-2.09575	0.00002	1.25437
H	-1.44527	-2.12760	-0.37327	H	-1.67536	-2.12965	-0.04911
H	-0.37960	-1.37494	-1.57970	H	-1.24391	-1.35825	-1.58738
F	1.11324	-2.35144	0.22889	F	0.88041	-2.33829	-0.66210
F	-0.14900	-1.35175	1.68109	F	0.38235	-1.41249	1.24099
Cl	2.13916	0.00000	-1.16060	Cl	2.68165	0.00001	0.00391
**14_{ax}**<br>**Energy (Hartree) = -3405.033515**<br>**LHVF (cm^{-1}) = 72.16**				**14_{eq}**<br>**Energy (Hartree) = -3405.031562**<br>**LHVF (cm^{-1}) = 58.41**			

C	-0.72525	0.00000	0.73703	C	0.58752	0.00000	-0.55975
C	0.13606	-1.25954	0.59050	C	-0.13828	1.26404	-0.10276
C	1.00528	-1.27465	-0.64949	C	-1.60399	1.26828	-0.50065
C	1.81528	-0.00000	-0.79310	C	-2.31860	-0.00000	-0.08070
C	1.00528	1.27465	-0.64949	C	-1.60399	-1.26828	-0.50063
C	0.13606	1.25953	0.59050	C	-0.13828	-1.26404	-0.10276
H	-1.19582	-0.00000	1.71676	H	0.59653	-0.00000	-1.64804
F	-0.65780	-2.35602	0.59453	F	0.45929	2.33991	-0.67438
F	0.91000	-1.35022	1.70129	F	-0.02531	1.41603	1.23348
H	1.68032	-2.12701	-0.59762	H	-2.08866	2.12939	-0.04346
H	0.36077	-1.37641	-1.52094	H	-1.66740	1.35755	-1.58457
F	2.40310	-0.00000	-2.02122	F	-3.56177	-0.00000	-0.63696
F	2.82806	0.00000	0.11131	F	-2.50173	0.00001	1.26267
H	1.68032	2.12701	-0.59764	H	-2.08865	-2.12938	-0.04342
H	0.36076	1.37640	-1.52094	H	-1.66741	-1.35757	-1.58455
F	-0.65780	2.35602	0.59453	F	0.45929	-2.33991	-0.67439
F	0.91000	1.35022	1.70128	F	-0.02530	-1.41603	1.23348
Br	-2.13550	0.00000	-0.58774	Br	2.42769	0.00000	0.00835
**15_{ax}**				**15_{eq}**			
**Energy (Hartree) = -1092.490066**				**Energy (Hartree) = -1092.495873**			
**LHVF (cm^{-1}) = 99.73**				**LHVF (cm^{-1}) = 107.94**			
C	-1.21759	0.00191	0.99323	C	-1.23239	0.00000	0.28104
C	-0.36855	-1.26420	1.00674	C	-0.61209	1.25541	-0.31187
C	0.74615	-1.25795	-0.01976	C	0.88517	1.25335	-0.07981
C	1.59415	-0.00248	0.00010	C	1.58551	0.00000	-0.56501
C	0.75009	1.25563	-0.01978	C	0.88517	-1.25335	-0.07982
C	-0.36460	1.26539	1.00672	C	-0.61209	-1.25541	-0.31187
H	-1.85749	0.00291	1.87026	H	-1.12085	0.00000	1.36263
H	-0.98209	-2.14912	0.84980	H	-1.03028	2.15357	0.13875
H	0.10612	-1.34545	1.98729	H	-0.79580	1.28671	-1.38684
F	1.56003	-2.32851	0.22249	F	1.43817	2.33159	-0.70071
F	0.23955	-1.44993	-1.26427	F	1.12510	1.41940	1.25262
H	2.26928	-0.00355	-0.85396	H	2.61840	0.00000	-0.22091
H	2.18013	-0.00338	0.91863	H	1.57006	0.00000	-1.65431
F	1.56730	2.32365	0.22250	F	1.43817	-2.33159	-0.70070
F	0.24411	1.44918	-1.26430	F	1.12509	-1.41940	1.25262
H	-0.97538	2.15220	0.84974	H	-1.03028	-2.15357	0.13874
H	0.11029	1.34517	1.98725	H	-0.79580	-1.28671	-1.38685
Cl	-2.36398	0.00365	-0.39178	Cl	-2.99706	0.00000	-0.03068
**16_{ax}**				**16_{eq}**			
**Energy (Hartree) = -3206.524022**				**Energy (Hartree) = -3206.530195**			
**LHVF (cm^{-1}) = 92.82**				**LHVF (cm^{-1}) = 100.46**			
C	0.69059	-0.00001	1.16091	C	-0.70625	0.00000	0.29375
C	-0.15639	1.26462	1.08587	C	-0.09335	1.25607	-0.30410
C	-1.16461	1.25711	-0.04557	C	1.40642	1.25323	-0.08075
C	-2.00787	0.00003	-0.11048	C	2.10346	0.00000	-0.57060
C	-1.16467	-1.25708	-0.04556	C	1.40642	-1.25323	-0.08075
C	-0.15644	-1.26462	1.08587	C	-0.09335	-1.25607	-0.30411
H	1.24190	-0.00002	2.09479	H	-0.59557	0.00000	1.37461
H	0.46293	2.15453	0.99513	H	-0.50573	2.15593	0.14841
H	-0.72990	1.34140	2.01331	H	-0.28100	1.28967	-1.37831
F	-2.00030	2.32563	0.11711	F	1.95501	2.33177	-0.70514
F	-0.53909	1.45197	-1.23446	F	1.65404	1.41941	1.25000
H	-2.59413	0.00004	-1.02786	H	3.13871	0.00000	-0.23360
H	-2.68300	0.00004	0.74465	H	2.08068	0.00000	-1.65980
F	-2.00041	-2.32556	0.11711	F	1.95501	-2.33177	-0.70513
F	-0.53917	-1.45196	-1.23446	F	1.65403	-1.41941	1.25000
H	0.46285	-2.15454	0.99511	H	-0.50573	-2.15594	0.14839
H	-0.72995	-1.34138	2.01333	H	-0.28100	-1.28966	-1.37832
Br	2.11532	-0.00003	-0.18579	Br	-2.63295	0.00000	-0.01570

References

(1) Zhao, Y.; Truhlar, D. G. The M06 Suite of Density Functionals for Main Group Thermochemistry, Thermochemical Kinetics, Noncovalent Interactions, Excited States, and Transition Elements: Two New Functionals and Systematic Testing of Four M06-Class Functionals and 12 Other Functionals. *Theor. Chem. Acc.* **2007**, *120*, 215-241.

(2) Kendall, R. A.; Dunning Jr., T. H.; Harrison, R. J. *J. Chem. Phys.* **1992**, *96*, 6796-6806.

(3) (a) Piscelli, B. A.; O'Hagan, D.; Cormanich, R. A. Contribution of Hyperconjugation and Inductive Effects to the Pseudo-anomeric Effect in 4-Substituted Methoxycyclohexanes. *J. Phys. Chem. A.* **2023**, *127*, 724-728; (b) Piscelli, B. A.; O'Hagan, D.; Cormanich, R. A. The Contribution of Non-classical CH_{ax}···OC Hydrogen Bonds to the Anomeric Effect in Fluoro and Oxa-methoxycyclohexanes. *Phys. Chem. Chem. Phys*. **2021**, *23*, 5845-5851.

(4) NBO 7.0, E. D. Glendening, J. K. Badenhoop, A. E. Reed, J. E. Carpenter, J. A. Bohmann, C. M. Morales, P. Karafiloglou, C. R. Landis, and F. Weinhold, Theoretical Chemistry Institute, University of Wisconsin, Madison, **2018**.

(5) Weinhold, F.; Landis, C. R. *Discovering Chemistry with Natural Bond Orbitals*, Wiley-VCH, **2012**; pp 145ff.

(6) (a) Badenhoop, J. K.; Weinhold, F. Natural Bond Orbital Analysis of Steric Interactions. *J. Chem. Phys.* **1997**, *107*, 5406-5421; (b) Badenhoop, J. K.; Weinhold, F. Natural Steric Analysis of Internal Rotation Barriers. *Int. J. Quantum Chem.* **1999**, *72*, 269-280.

(7) Menucci, B.; Cammi, R.; Tomasi, J. Excited states and solvatochromic shifts within a nonequilibrium solvation approach: A new formulation of the integral equation formalism method at the self-consistent field, configuration interaction, and multiconfiguration self-consistent field level. *J. Chem. Phys.* **1998**, *109*, 2798.

(8) Gaussian 16, Revision C.01, M. J. Frisch, G. W. Trucks, H. B. Schlegel, G. E. Scuseria, M. A. Robb, J. R. Cheeseman, G. Scalmani, V. Barone, G. A. Petersson, H. Nakatsuji, X. Li, M. Caricato, A. V. Marenich, J. Bloino, B. G. Janesko, R. Gomperts, B. Mennucci, H. P. Hratchian, J. V. Ortiz, A. F. Izmaylov, J. L. Sonnenberg, D. Williams-Young, F. Ding, F. Lipparini, F. Egidi, J. Goings, B. Peng, A. Petrone, T. Henderson, D. Ranasinghe, V. G. Zakrzewski, J. Gao, N. Rega, G. Zheng, W. Liang, M. Hada, M. Ehara, K. Toyota, R. Fukuda, J. Hasegawa, M. Ishida, T. Nakajima, Y. Honda, O. Kitao, H. Nakai, T. Vreven, K. Throssell, J. A. Montgomery, Jr., J. E. Peralta, F. Ogliaro, M. J. Bearpark, J. J. Heyd, E. N. Brothers, K. N. Kudin, V. N. Staroverov, T. A. Keith, R. Kobayashi, J. Normand, K. Raghavachari, A. P. Rendell, J. C. Burant, S. S. Iyengar, J. Tomasi, M. Cossi, J. M. Millam, M. Klene, C. Adamo, R. Cammi, J. W. Ochterski, R. L. Martin, K. Morokuma, O. Farkas, J. B. Foresman, and D. J. Fox, Gaussian, Inc., Wallingford CT, 2016.
